# Development of MDM2‐Targeting PROTAC for Advancing Bone Regeneration

**DOI:** 10.1002/advs.202415626

**Published:** 2025-03-24

**Authors:** Sol Jeong, Jae‐Kook Cha, Wasim Ahmed, Jaewan Kim, Minsup Kim, Kyung Tae Hong, Wonji Choi, Sunjoo Choi, Tae Hyeon Yoo, Hyun‑Ju An, Seung Chan An, Jaemin Lee, Jimin Choi, Sun‐Young Kim, Jun‐Seok Lee, Soonchul Lee, Junwon Choi, Jin Man Kim

**Affiliations:** ^1^ Department of Oral Microbiology and Immunology School of Dentistry and Dental Research Institute Seoul National University Seoul 08826 Republic of Korea; ^2^ Department of Periodontology Research Institute of Periodontal Regeneration College of Dentistry Yonsei University Seoul 03722 Republic of Korea; ^3^ Department of Oral Medicine Infection and Immunity Harvard School of Dental Medicine Boston 02115 USA; ^4^ Department of Molecular Science and Technology Ajou University Gyeonggi‐do 16499 Republic of Korea; ^5^ TARS Scientific Seoul 01717 Republic of Korea; ^6^ Department of Pharmacology Korea University College of Medicine Korea University Seoul 02841 Republic of Korea; ^7^ Department of Orthopaedic Surgery CHA Bundang Medical Center CHA University School of Medicine Gyeonggi‐do 13488 Republic of Korea; ^8^ Department of Conservative Dentistry and Dental Research Institute School of Dentistry Seoul National University Seoul 08826 Republic of Korea; ^9^ Advanced College of Bio‐convergence Engineering Ajou University Gyeonggi‐do 16499 Republic of Korea; ^10^ Dental Multiomics Center School of Dentistry and Dental Research Institute Seoul National University Gwanak‐ro 1, Gwanak‐gu Seoul 08826 Republic of Korea; ^11^ Innovative Pharmaceutical Sciences Program College of Transdisciplinary Innovations Seoul National University Gwanak‐ro 1, Gwanak‐gu Seoul 08826 Republic of Korea

**Keywords:** bone, MDM2, osteoporosis, PROTAC, regenerative medicine

## Abstract

Proteolysis‐targeting chimeras (PROTACs) degrade target proteins through the ubiquitin‐proteasome system. To date, PROTACs are primarily used to treat various diseases; however, they have not been applied in regenerative therapy. Herein, this work introduces MDM2‐targeting PROTACs customized for application in bone regeneration. An MDM2‐PROTAC library is constructed by combining Nutlin‐3 and CRBN ligands with various linker designs. Through a multistep validation process, this work develops MDM2‐PROTACs (CL144 and CL174) that presented potent degradation efficiency and a robust inductive effect on the biomineralization. Next, this work performs whole‐transcriptome analysis to dissect the biological effects of the CL144, and reveals the upregulation of osteogenic marker genes. Furthermore, CL144 effectively induced bone regeneration in bone graft and ovariectomy (OVX) models after local and systemic administration, respectively. In the OVX model, the combination treatment with CL144 and alendronate induced a synergistic effect. Overall, this study demonstrates the promising role of MDM2‐PROTAC in promoting bone regeneration, marking the first step toward expanding the application of the PROTAC technology.

## Graphical abstract

This work presents a creative pipeline for developing MDM2‐targeting PROTACs (MDM2‐PROTACs) for application in bone regeneration. The developed PROTACs (CL144 and CL174) are validated for their degradation efficiency and osteogenic effects in human BMSCs. The MDM2‐PROTACs demonstrate regenerative potency in bone defect animal models, showing a particularly synergistic effect with bisphosphonates.

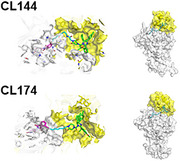

## 1 Introduction

Bone regeneration is a multifaceted process driven by the interplay between bone‐forming cells (osteoblasts) and bone‐resorbing cells (osteoclasts). Under physiological conditions, a delicate balance known as osteoblast‐osteoclast homeostasis is maintained. This homeostatic mechanism allows for efficient bone remodeling, compensating for microdamage and mechanical stresses induced by regular physical activity.^[^ ^1^ ^]^ The sustainability of bone remodeling can be disrupted by various pathogenic conditions, including traumatic fractures or metabolic disorders, such as osteoporosis, leading to bone destruction. In response to damage, monocyte‐derived osteoclasts replace the damaged bone structures, while neovascularization is initiated from the surrounding blood vessel networks, which supply crucial cellular sources for the regeneration process.^[^ ^2^ ^]^ Mesenchymal stromal cells (MSCs), a specific subpopulation of nonhematopoietic cells derived from bone marrow niches, play a pivotal role in bone regeneration. MSCs serve as precursors to pre‐osteoblasts, are multipotent, and are capable of differentiating into adipose tissue, cartilage, neural tissue, and bone.^[^ ^3^ ^]^ Owing to their cellular properties and regenerative potential, MSCs have emerged as promising targets for therapeutic interventions to promote bone regeneration.

Considering recent advances in cell biology, numerous technologies targeting MSCs for bone regeneration are emerging. However, significant progress in osseous tissue engineering remains elusive. Bone morphogenetic protein‐2 (BMP‐2) is a well‐established osteogenic factor known for its ability to induce MSC differentiation, and is one of the few reagents approved by the Food and Drug Administration (FDA) for various indications in bone regeneration. Despite its validated osteoinductive properties and widespread use, BMP‐2 is associated with adverse effects, such as life‐threatening inflammation, adipogenesis, and cystic bone formation, which limit its clinical application.^[^ ^4^ ^]^ Stem cell‐based therapies offer a promising alternative for replenishing osteogenic cells with appropriate scaffolds, representing a more integrated approach to tissue engineering triad.^[^ ^5^ ^]^ Nevertheless, standardization of isolation and characterization procedures to ensure the production of readily available regenerative cells poses a major challenge.^[^ ^6^ ^]^ Consequently, there is an urgent need within the scientific community to explore and develop novel therapeutic approaches for bone regeneration.

Proteolysis‐targeting chimeras (PROTACs) have emerged as a novel therapeutic approach, offering the potential to expand the scope of protein targets whose functions are difficult to modulate using conventional small molecules. PROTACs are bifunctional molecules in which a ligand for a protein of interest (POI) is connected to an E3 ligase ligand using a linker. This architecture promotes the formation of a ternary complex between the POI and an E3 ligase, inducing ubiquitination and subsequent degradation of the target protein by the ubiquitin–proteasome system (UPS).^[^ ^7^ ^]^ In contrast to traditional small‐molecule inhibitors that disrupt protein functions through an “occupancy‐driven” mechanism, PROTACs employing an “event‐driven” degradation mechanism have demonstrated advantages in improving potency and selectivity.^[^ ^8^ ^]^ Recent advancements in PROTAC technology have highlighted its potential as a versatile therapeutic option for various diseases, especially cancer.^[^ ^8c^ ^]^ This approach has also achieved success in the targeted degradation of “undruggable” oncogenic proteins, which have been challenging to modulate with traditional small molecules such as Kirsten rat sarcoma virus (KRAS), B‐cell lymphoma‐extra large (Bcl‐xL), signal transducer and activator of transcription (STAT3), and mouse double minute 2 homolog (MDM2).^[^ ^9^ ^]^ In addition to oncology, the application of PROTAC has been extended to other areas over the past few years, including neurodegenerative disorders, inflammatory diseases, and viral infection.^[^ ^10^ ^]^ Although this technology has a wide‐ranging impact, its application in tissue regeneration remains limited. A recent study has shown that targeting senescent bone marrow stromal cells can enhance osteoprogenitor functions.^[^ ^11^ ^]^ However, the use of PROTACs specifically for promoting hard tissue regeneration remains largely unexplored.

In a previous study, we reported that MDM2–p53 signaling is a key transcriptional regulatory axis in the biomineralization process, and p53 serves as a core transcription factor that induces multiple osteogenic marker genes in MSCs.^[^ ^12^ ^]^ MDM2 is an E3 ubiquitin ligase that is widely recognized as a negative regulator of p53 expression. It binds to the N‐terminal transactivation domain of p53, directly repressing its transcriptional function via p53 degradation.^[^ ^13^ ^]^ Building upon this background, we targeted MDM2 using a small‐molecule protein–protein interaction (PPI) inhibitor and observed that disrupting the MDM2–p53 interaction induced the transcriptional activation of p53, ultimately leading to hard tissue regeneration in various preclinical models.^[^ ^12^ ^]^ However, it is worth noting that elevated levels of p53 are known to stimulate the expression of MDM2 through the p53–MDM2 autoregulatory feedback loop, consequently attenuating the effects of the small molecule inhibitors.^[^ ^14^ ^]^ To address this, we propose employing the PROTAC approach, which not only disrupts the MDM2–p53 interaction but also sustains the effect of p53 through PROTAC‐induced MDM2 degradation. Hence, we hypothesize that the MDM2‐targeting PROTAC could significantly enhance the efficacy of the osteogenic differentiation of MSCs and promote bone regeneration.

In this study, we systematically engineer a PROTAC platform for the targeted degradation of MDM2 and have successfully developed MDM2‐PROTACs by combining the MDM2 ligand (Nutlin‐3) with the cereblon ligand (thalidomide). Furthermore, we demonstrate that MDM2‐PROTAC effectively activates the osteogenic differentiation program in MSCs, resulting in significant enhancement of bone regeneration.

## 2 Results

### 2.1 Development of MDM2‐Targeting PROTACs

**Figure 1:**
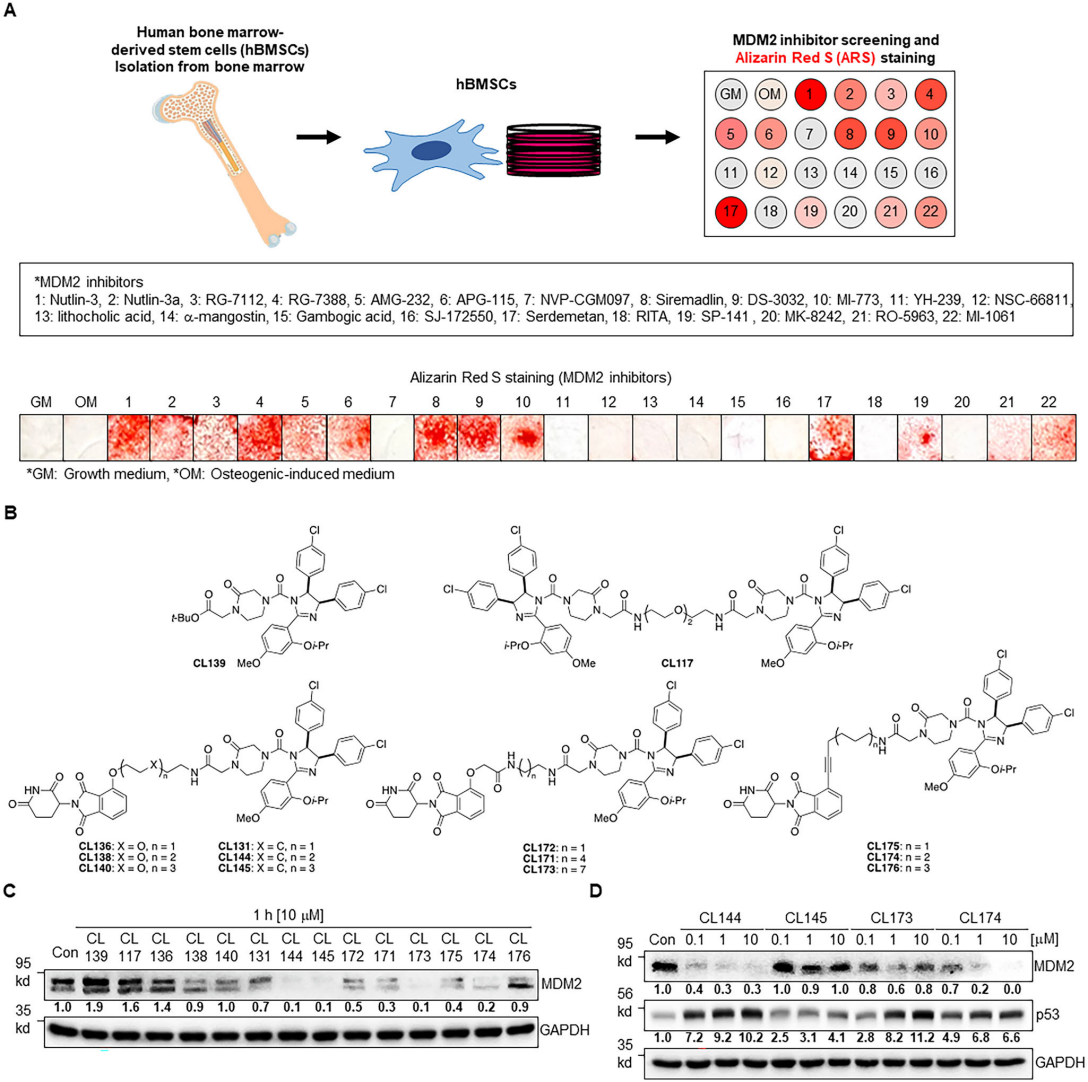
Development of MDM2‐targeting PROTAC compounds. A) Schematic representation of osteogenic differentiation using human bone marrow‐derived stem cells (hBMSCs), along with a list of MDM2 inhibitors, and their screening through Alizarin Red S (ARS) staining in hBMSCs. B) Structural schematic diagram of MDM2‐PROTAC compounds synthesized based on CL139 (Nutlin‐3). C) Degradation efficiency test of synthesized MDM2‐PROTAC compounds in hBMSCs; the bold numbers relative quantitative values, and GAPDH was used as the loading control. D) Degradation efficiency test of the initially selected four compounds (CL144, CL145, CL173, and CL174) with different concentration in hBMSCs; the bold numbers represent relative quantitative values, and GAPDH was used as the loading control.

To design an MDM2‐targeting PROTAC, we initially explored candidate MDM2 ligands with intrinsic osteogenic potency. We evaluated previously reported MDM2 inhibitors that disrupt MDM2–p53 protein–protein interaction using Alizarin Red S (ARS) staining assay in cultured human bone marrow‐derived mesenchymal stromal cells (hBMSCs) (**Figure** **1**A). Through this screening process, several inhibitors exhibited significant effects on biomineralization (Figure 1A), supporting the relevance of MDM2 inhibition in inducing osteogenic differentiation of MSCs. We selected Nutlin‐3 based on multiple lines of evidence supporting its osteogenic potential. First, in our screening using hBMSCs, Nutlin‐3 demonstrated superior osteogenic potency without adversely affecting cell viability (Figure 1A and Figures S1 and S2, Supporting Information). Second, our previous study reported that Nutlin‐3 effectively induced osteogenic differentiation in another type of MSC, human dental pulp stromal cells (hDPSC), further supporting its potential as a strong osteogenic agent.^[^ ^12^ ^]^ Using the method in a previous study,^[^ ^12^ ^]^ we quantified the Alizarin Red S (ARS) staining results from Figure 1A to evaluate the osteogenic potency of MDM2 inhibitors (Figure S1A, Supporting Information). To further investigate the relationship between the binding affinity of the inhibitors and their osteogenic potency, we applied the values of binding affinity of MDM2 inhibitors (*K_d_*) and performed a Pearson correlation analysis. While *K_d_* values were not available for all compounds, the analysis showed a Pearson correlation coefficient (*r*) of −0.11, indicating no significant correlation (Figure S1B, Supporting Information). This suggests that the binding affinity might not be the only major factor inducing osteogenic activity of the inhibitors as reflected by ARS staining results.

Thalidomide was utilized as an E3 ligase ligand, and a panel of MDM2‐PROTACs was synthesized by combining Nutlin‐3 (MDM2 ligand) and thalidomide (E3 ligase ligand) with various linkers with different properties, such as length, rigidity, and hydrophobicity (Figure 1B, Supplementary materials). Additionally, considering that MDM2 serves as an E3 ubiquitin ligase, the Nutlin‐3 dimer, CL117, was prepared.^[^ ^15^ ^]^ Next, we evaluated the degradation efficacy of the MDM2‐PROTAC library using cultured hBMSCs (Figure 1C). The library compounds were treated on hBMSCs at 10 μΜ for 1 h, and the level of MDM2 was evaluated by the quantified results of western blot. While the Nutlin‐3 analog, CL139, and the Nutlin‐3 dimer, CL117, did not induce the degradation of MDM2, MDM2‐PROTACs utilizing thalidomide as an E3 ligase ligand efficiently promoted MDM2 degradation. The MDM2‐PROTACs with hydrocarbon linkers showed better degradation efficiencies than those with PEG linkers (CL136, CL138, and CL140 versus CL131, CL144, and CL145). Notably, PROTACs in which thalidomide and Nutlin‐3 were connected by a 12‐ or 15‐atom distance (CL144 and CL145, respectively) tended to be more potent than those with a 9‐atom distance (CL131). When thalidomide was conjugated to a hydrocarbon linker via oxyacetamide, PROTAC exhibited reduced MDM2 degradation efficiency compared to PROTACs possessing a phenyl ether connection (CL171 and CL173 versus CL144 and CL145). MDM2‐PROTAC, with a rigid alkyne bond between the thalidomide and the linker, also effectively decreased MDM2 levels (CL174).

We further evaluated the dose‐dependent effect (0.1 µM to 10 µM) and the change in downstream p53 levels with four compounds (CL144, CL145, CL173, and CL174), which showed a remarkable decrease in MDM2 levels by over 80% compared to the basal level (Figure 1D). CL144 and CL174 were more potent than CL145 and CL173. Both the compounds decreased MDM2 levels and increased p53 expression over the entire concentration range tested. CL144 induced robust MDM2 degradation and elevated p53 levels in a dose‐dependent manner. In contrast, CL173 showed a potent capability to enhance p53 levels, but a less potent degradation effect on MDM2 (Figure 1D).

### 2.2 Validation of MDM2‐PROTAC Binding for the Formation of an MDM2–PROTAC–CRBN Ternary Complex

**Figure 2:**
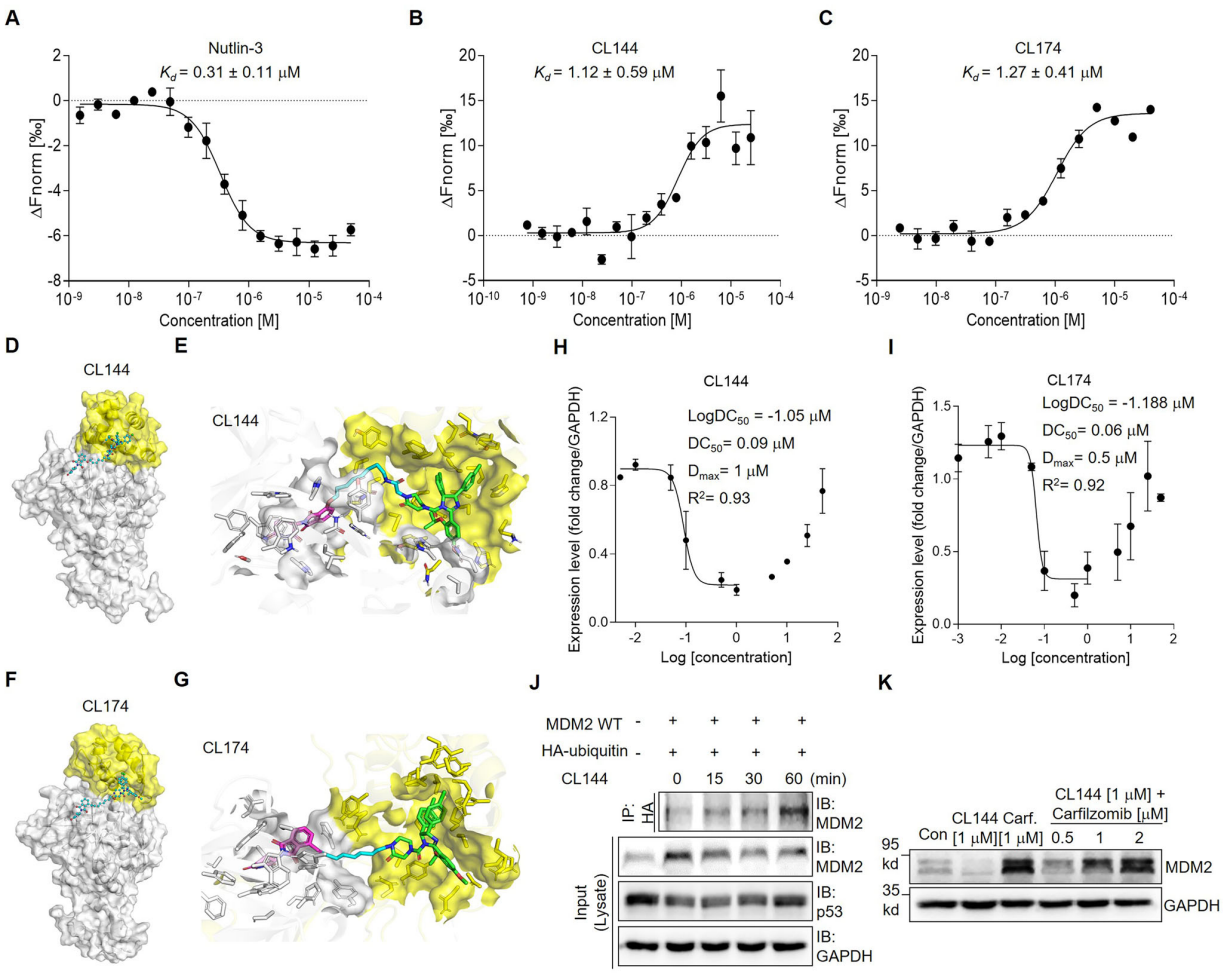
Validation of the synthesized MDM2‐targeting PROTAC compounds. A–C) Evaluation of dissociation constant (*K_d_*) values of MDM2 ligand (Nutlin‐3) and MDM2‐PROTACs (CL144, CL174) though microscale thermophoresis (MST) assay; mean with SEM; n = 3. D,E) Predicted ternary structures of MDM2–CL144–CRBN. F,G) Predicted ternary structures of MDM2–CL174–CRBN; CRBN is shown as white illustrations and surfaces. MDM2 is shown as yellow illustrations and surfaces; Thalidomide, Nutlin‐3, and the linker components in the PROTAC are shown in magenta, green, and cyan, respectively. H,I) Evaluation of the maximal degradation concentration (*D_max_*) and the half of maximal degradation concentration (*DC_50_*) values according to the concentration of the MDM2‐PROTACs (CL144, CL174); n = 2. J) Immunoprecipitation assay of MDM2 wild type and HA‐tagged ubiquitin was confirmed after treatment with 10 µM of CL144. K) Immunoblot assay under the condition of blocking the intracellular proteasome system using the proteasome inhibitor (Carfilzomib, Carf).

To evaluate the formation of the MDM2–PROTAC–CRBN ternary complex with the selected PROTAC compounds, CL144 and CL174, the binding affinity of these compounds toward MDM2 was first examined. Compared to Nutlin‐3 (*K_d_* = 0.31 µM), both PROTACs, CL144 and CL174, exhibited slightly reduced binding affinities with *K_d_* values of 1.12 and 1.27 µM, respectively. This indicated that the attachment of the linker–CRBN ligand to Nutlin‐3 did not significantly impair the binding affinity of Nutlin‐3 toward MDM2 (**Figure** **2**A–C). To gain insight into the formation of the ternary complex between MDM2 and CRBN induced by CL144 or CL174, molecular dynamics (MD) simulations were conducted using the X‐ray co‐crystal structures of MDM2 with Nutlin‐3a and CRBN with (*S*)‐thalidomide. The computational studies predicted that both compounds, CL144 and CL174, successfully bind to MDM2 and CRBN and induce the formation of stabilized MDM2–PROTAC–CRBN ternary complexes with binding energies of −86.04 and −76.02 kcal mol^−1^, respectively, relative to the Nutlin‐3a‐bound MDM2 and (*S*)‐thalidomide‐bound CRBN complexes (Figure 2D–G). Additionally, in the predicted ternary structures, the Nutlin‐3 and thalidomide components in the synthesized PROTAC molecules showed similar binding conformations to their target proteins compared with the co‐crystal structures of each ligand and its target protein, which was consistent with the binding affinities of CL144 and CL174 to MDM2. The heavy‐atom root‐mean‐square deviation (RMSD) values for Nultin‐3 and thalidomide were ≈1.35 and 3.14 Å, respectively. Notably, the differences in the linkers, particularly in their rigidity, between CL144 and CL174 resulted in the formation of distinct binding poses for MDM2–PROTAC–CRBN.

### 2.3 Validation of the Degradation Characteristics of MDM2‐PROTACs

To evaluate the degradation characteristics of the selected compounds, we evaluated MDM2 level upon treatment with either CL144 or 174 with an expanded concentration range (1 nM to 50 µM). Based on the quantified western blot results, we estimated the half‐maximal degradation concentrations (*DC_50_*, CL144 = 0.09 μΜ; CL174 = 0.06 μΜ) and maximal degradation concentrations (*D_max_*, CL144 = 1 μΜ; CL174 = 0.5 μΜ) (Figure 2H,I and Figure S3A,B, Supporting Information). The quantified graphs indicated an optimal window of degradation efficiency ranging from 0.1 to 10 µM, and the efficiency decreased over the concentration range (Figure 2H,I). This phenomenon indicates the “hook effect,” which refers to the inability to form a ternary complex when the concentration of PROTAC compound exceeds a certain threshold.^[^ ^16^ ^]^ Next, we examined PROTAC‐mediated ubiquitination of MDM2 in HeLa cells overexpressing MDM2 and HA‐tagged ubiquitin. CL144 increased the ubiquitination level of MDM2 by 2.65‐fold within 60 min (Figure 2J). Since PROTAC operates based on the intracellular proteasome degradation system, we investigated MDM2 proteolysis following treatment with the proteasome inhibitor carfilzomib. Treatment with varying concentrations (0.5–2 μΜ) of carfilzomib blocked the efficacy of PROTAC in a dose‐dependent manner. These results demonstrated that MDM2‐PROTAC induced target degradation by utilizing UPS (Figure 2K).

To further assess the specificity of CL144, we evaluated its off‐target degradation activity on neo‐substrates. Western blot analysis was performed after treating cells with CL144 at 10 µM. Under conditions where MDM2 degradation was observed at ≈70%, IKZF1 showed a reduction of about 35% among the evaluated neo‐substrates (IKZF1, GSPT1, CK1α, and IKZF3/Aiolos). These findings suggest that CL144 exhibits minimal off‐target degradation activity (Figure S3C,D, Supporting Information).

Next, we expanded the selectivity profiling of CL144 at the whole proteome level through global proteomics analysis in two cell models: hBMSCs and MDM2‐overexpressing HeLa cells. Despite employing multi‐dimensional peptide fractionation to maximize proteome coverage, we encountered challenges in detecting MDM2 in hBMSCs. Among the identified 6388 proteins in hBMSCs, 75 downregulated proteins were extracted (|Log_2_(Fold Change)| < 0.35 and *p*‐value < 0.001; Figure S4, Supporting Information). Notably, MDM4, known for its heterodimer formation with MDM2, and USP42, a ubiquitin ligase that directly interacts with MDM2, showed significant downregulation.^[^ ^17^ ^]^ In addition, we observed the downregulation of transcription regulators (DDX1, ELF2) and other ubiquitin‐processing proteins (USP10, SH3RF1, USP42). These findings indirectly support the target engagement of CL144 with MDM2.

In the MDM2‐overexpressing HeLa cell model, we successfully detected MDM2 and observed its selective downregulation following CL144 treatment (Figure S5A, Supporting Information). A total of 8353 proteins were identified, and the volcano plot revealed MDM2 as one of 35 significantly downregulated proteins (|Log_2_(Fold Change)| < 0.35 and *p*‐value < 0.001; Figure S5A,B, Supporting Information). Notably, CL144 treatment resulted in significantly fewer downregulated proteins compared to Nutlin‐3, with 35 downregulated proteins for CL144 versus 267 for Nutlin‐3 (Figure S5C, Supporting Information). This selective effect highlights the potential advantage of PROTAC degraders over conventional inhibitors, as PROTACs can provide more targeted degradation, minimizing off‐target effects and reducing the perturbation of the proteome.

While CL144 demonstrated strong selectivity for MDM2 across the proteome, we also observed downregulation of additional ubiquitin ligases and processing proteins, such as USP29, USP48, RBX1, and ZNF598. These findings suggest that, although CL144 effectively targets MDM2, further optimization could be considered to refine its specificity in future studies. It will be crucial to carefully examine the downregulated proteins, as some of these may result directly from PROTAC‐induced degradation, while others could reflect indirect changes in protein expression mediated by other signaling pathways.

### 2.4 Functional Dynamics of the Developed MDM2‐PROTACs

**Figure 3:**
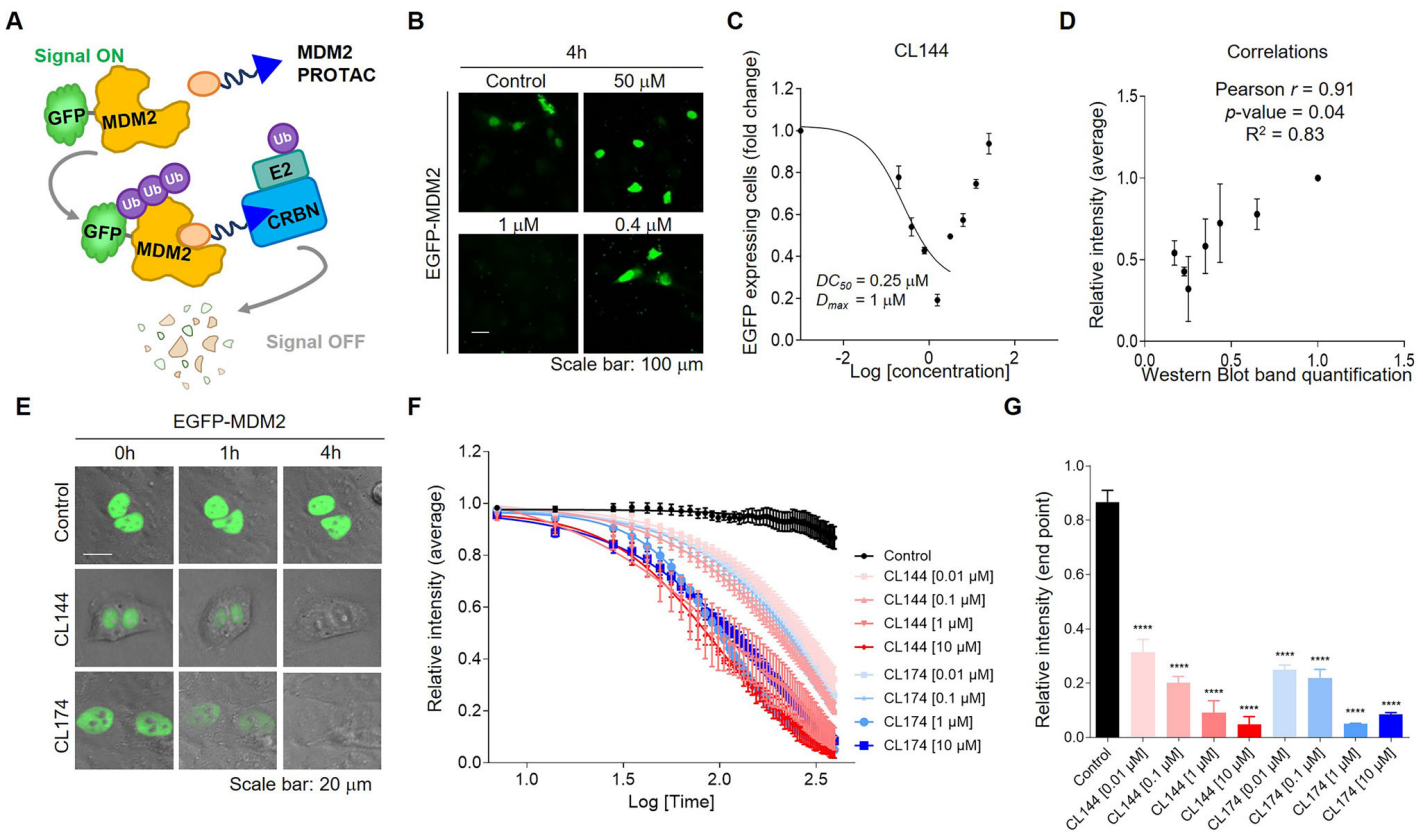
MDM2 proteolysis functional evaluation of MDM2‐PROTAC using EGFP‐MDM2 expressing cells. A) Experimental design of the PROTAC dynamics using EGFP‐MDM2 construct. B) luorescence images of EGFP‐MDM2 expressing cells treated with different concentrations of CL144; scale bar: 100 µm. C) Quantification of the number of EGFP‐MDM2 expressing cells and evaluation of *D_max_* and *DC_50_* values; mean with SEM; n = 3. D) Pearson correlation analysis between quantitative results of western blot and EGFP‐MDM2 fluorescence intensity; Pearson *r* = 0.91; *p* < 0.05; mean with SEM; n = 3. E) Still images from live imaging of EGFP‐MDM2 expressing cells treated with MDM2‐PROTACs (CL144 and CL174); scale bar = 20 µm. F) Quantification results of live imaging of EGFP‐MDM2 expressing cells treated with newly developed MDM2‐PROTACs (CL144, CL174); Quantification values were calculated from the EGFP signals; mean with SEM; n = 3 to 8. G) Maximal degradation efficiency of MDM2‐PROTACs at 0.01 to 10 µM concentrations; ANOVA Bonferroni test: ****p < 0.0001; mean with SEM; n = 3 to 8.

To investigate the degradation dynamics of MDM2‐PROTAC, we used the EGFP‐MDM2 construct, which enables real‐time monitoring of MDM2 proteolysis by detecting the fused EGFP signal (**Figure** **3**A). As a supporting model to evaluate the performance of MDM2‐PROTAC, we first applied this model to determine *DC_50_* and *D_max_* and compared the resultant values with the western blot data (Figure 2H,I). EGFP‐MDM2 expressing cells were treated with CL144 in a concentration 1 nM to 50 µM, and the changes in EGFP signal intensity were used to calculate *DC_50_* and *D_max_* values (*DC_50_* = 0.25 μΜ, *D_max_* = 1 μΜ). In this model, CL144 showed 84% EGFP‐MDM2 maximal degradation efficacy at 1 µM treatment condition (Figure 3B,C). These results showed a strong correlation with the previously presented western blot results, demonstrating the relevance of our imaging‐based model (Pearson's *r* = 0.91, *p‐*value = 0.04) (Figure 3D). To evaluate the degradation kinetics of MDM2‐PROTAC, we performed live‐imaging of EGFP‐MDM2 expressing cells upon after PROTAC treatment. Treatment with CL144 or CL174 for 4 h almost completely turned off the nuclear EGFP signal (Figure 3E). We quantified the time‐course changes in EGFP signal dynamics following treatment with CL144 and CL174 at concentrations ranging from 0.01 to 10 µM. Both PROTACs exhibited distinct degradation dynamics depending on the concentration, with more rapid degradation observed at higher concentrations (Figure 3F). The maximum degradation efficiency was achieved at 10 µM for CL144 and at 1 µM for CL174 (Figure 3G). Additionally, we conducted a comparative assessment of our compounds with a previously reported for MDM2‐PROTAC (MD224)^[^ ^18^ ^]^ under identical experimental conditions. At low concentrations (0.01–0.1 µM), MD224 showed greater degradation efficiency of EGFP‐MDM2 compared to higher concentrations (1–10 µM), indicating the presence of a hook effect (Figure S6, Supporting Information). CL144 and CL174 demonstrated superior performance at higher concentrations compared to MD224 while exhibiting similar effects at lower concentrations (Figure 3F,G). These findings underscore the importance of identifying optimal concentration windows for different PROTAC systems.

### 2.5 Comparative Analysis of MDM2‐PROTAC versus MDM2 Inhibitor

**Figure 4:**
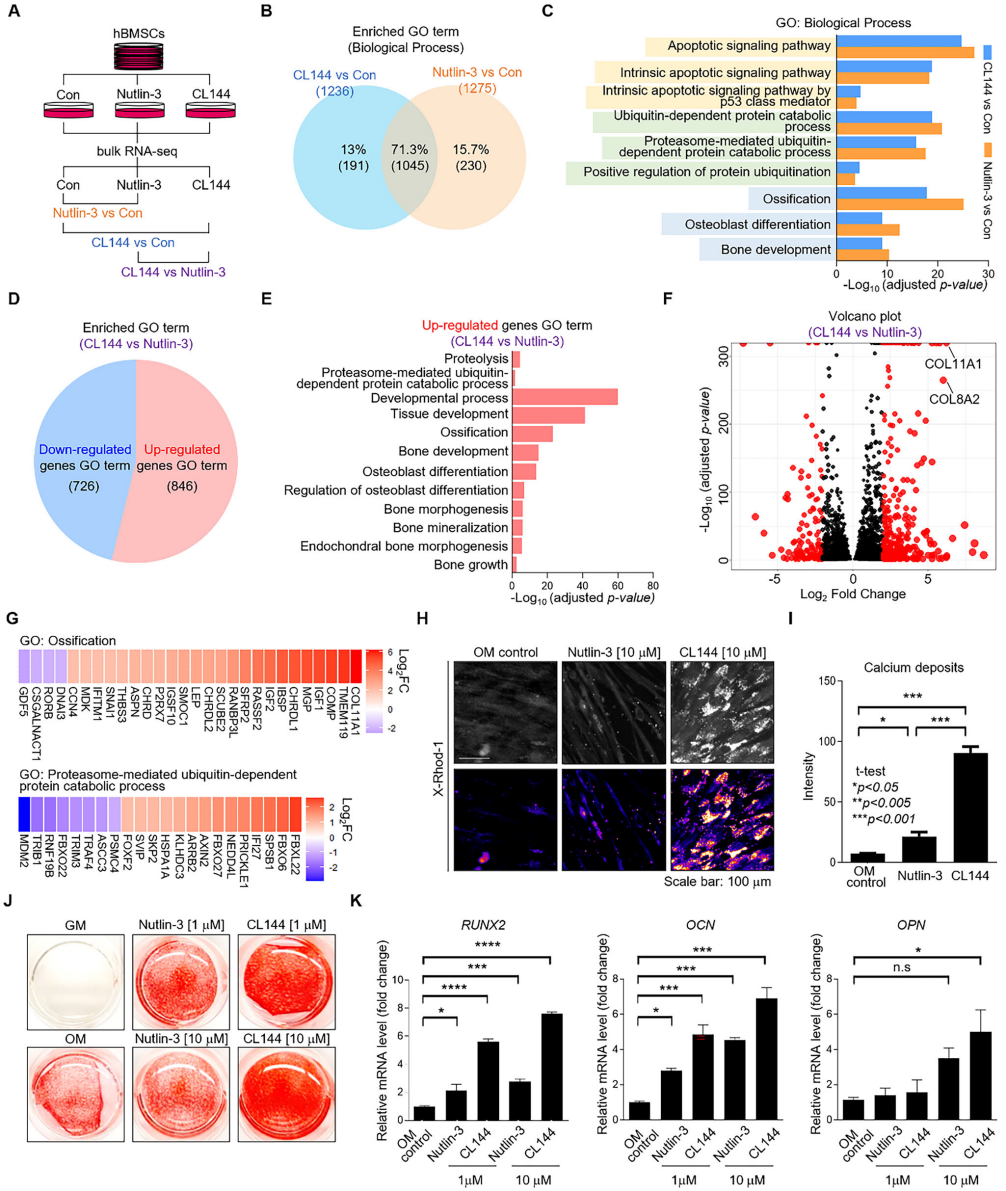
Comparative analysis of MDM2 targeting small molecule (Nutlin‐3) and PROTAC (CL144). A) Schematic diagram of bulk RNA‐seq in cultured hBMSC models. B) Venn diagram of between‐group comparisons of enriched gene ontology (GO) terms (*padj*<0.05) between two comparison pairs (CL144 versus Con and Nultin‐3 versus Con). C) Representative overlapped enriched GO terms of two comparison pairs (CL144 versus Con and Nultin‐3 versus Con). D) Proportion of enriched GO terms analyzed from upregulated and downregulated DEGs of CL144 versus Nutlin‐3 comparison pair (*padj*<0.05). E) Representative GO terms of up‐regulated DEGs of CL144 versus Nutlin‐3 comparison pair (*padj*<0.05). F) Volcano plot showing gene profiles of CL144 versus Nutlin‐3 comparison pair. DEGs are pointed by red dots (*padj*<0.05, │log_2_FC│> 1.5), and bone regeneration‐related genes are displayed (*COL11A1, COL8A2*). G) Gene heatmaps of representative GO terms of Ossification (GO:0001503) and Proteasome‐mediated ubiquitin‐dependent protein catabolic process (GO:0043161), FC = fold change. H) Calcium deposition staining (X‐Rhod‐1) of cultured hBMSCs treated with Nutlin‐3 and CL144 under osteogenic differentiation conditions. Images are pseudo‐colored by intensity. I) Quantitative data of calcium deposition images (H), Student's *t*‐test: *p < 0.05, **p < 0.005, ***p < 0.001, mean with SEM; n = 3. J,K) ARS staining (J) expression level of osteogenic marker genes (K) of hBMSCs by treatment with Nutlin‐3 and CL144 under osteogenic differentiation conditions; ANOVA Bonferroni test: ns = non‐significant, *p < 0.05, **p < 0.01, ***p < 0.001, ****p < 0.0001; mean with SEM; n = 4.

Next, we dissected the differential effects of MDM2‐PROTAC and MDM2 inhibitors. First, we compared the whole‐transcriptome profiles of hBMSCs treated with CL144 and Nutlin‐3 (a POI ligand of CL144) using bulk RNA sequencing (**Figure** **4**A). When comparing CL144 and Nultin‐3 to the control group (Con), we detected a total of 6932 (CL144 versus Con) and 7745 (Nutlin‐3 versus Con) differentially expressed genes (DEGs) (*padj*<0.05). Gene ontology (GO) analysis detected 1236 (CL144 versus Con) and 1275 (Nutlin‐3 versus Con) enriched GO terms in the Biological Process category. Among the different GO terms, 71.3% overlapped between CL144 and Nutlin‐3 groups, suggesting that CL144 and Nutlin‐3 induced similar biological effects (Figure 4B). Representative commonly enriched GO terms were related to p53, apoptotic signaling (Figure 4C, light yellow), and UPS‐based protein degradation processes (Figure 4C, light green). Notably, we observed a significant enrichment of multiple GO terms related to bone regeneration in both groups (Figure 4C, light blue).

To directly compare the effects of CL144 and Nutlin‐3, we conducted a head‐to‐head comparative analysis of the two compounds (CL144 versus Nutlin‐3). We identified 4339 differentially expressed genes (DEGs) (2119 up‐regulated and 2220 down‐regulated genes; *padj*<0.05). GO analysis was performed based on either the upregulated or downregulated genes in the CL144 group, revealing the enrichment of 726 and 846 GO terms, respectively (Figure 4D). When analyzing the enriched GO terms for the upregulated genes in the CL144 group, we observed an enrichment of GO terms related to protein degradation, such as proteolysis (GO:0006508) and proteasome‐mediated ubiquitin‐dependent protein catabolic processes (GO:0043161), as well as various GO terms associated with bone regeneration, including ossification (GO:0001503), osteoblast differentiation (GO:0001649), and bone mineralization (GO:0030282) (Figure 4E). The volcano plot showed significantly upregulated expression of collagen subtypes such as *COL11A1* (log_2_FC = 6.23) and *COL8A2* (log_2_FC = 6.02) in the CL144 group (Figure 4F). *COL11A1* is involved in multiple enriched ossification processes (Figure 4G) and plays an essential role in trabecular bone formation.^[^ ^19^ ^]^ In addition to *COL11A1*, multiple genes, *TMEM119, IGF1*, and *P2RX7*, which are known to potently induce ossification (Figure 4G), showed higher expression levels in CL144 than in Nutlin‐3 treatment.^[^ ^20^ ^]^ Moreover, we constructed a heatmap of DEGs related to proteasome‐mediated ubiquitin‐dependent protein catabolic processes (GO:0043161). We confirmed increased levels of genes encoding E3 ligase (*FBXL22* and *NEDD4L*) and subunits of the ubiquitin protein ligase complex (*FBXO6* and *FBXO27*) (Figure 4G). Collectively, the transcriptome profiling results predicted higher biomineralization potency and enhanced activity of the UPS of CL144 because of enriched gene sets related to biological processes.

To validate the results of the comparative analysis, we evaluated the biomineralization potency of CL144 and Nutlin‐3 by monitoring intracellular calcium deposits in hBMSCs. Calcium deposits were significantly increased with Nutlin‐3 or CL144 treatment compared to the control group (*p* < 0.05). Interestingly, compared to Nutlin‐3 treatment, CL144 treatment resulted in a more potent increase in calcium deposits (*p* < 0.001) (Figure 4H,I). The results of ARS staining also clearly demonstrated that CL144 significantly enhanced biomineralization in hBMSCs compared to the Nutlin‐3 and control groups (Figure 4J). We verified the mRNA levels of osteogenic differentiation marker genes including *RUNX2, OCN*, and *OPN*. In case of *RUNX2* and *OCN*, gene expression levels significantly increased in all experimental groups, while *OPN* was increased only in 10 μΜ CL144 treated group (Figure 4K). When comparing Nutlin‐3 and CL144, CL144 significantly increased the expression levels of *RUNX2* and *OCN* compared to Nutlin‐3 at the same concentrations (Figure 4K). These results indicate that MDM2‐PROTAC (CL144) upregulated osteogenic differentiation marker genes more effectively than the MDM2 inhibitor (Nutlin‐3), leading to a superior inductive effect on biomineralization in vitro. To validate these findings at the protein level, we treated cells with the MDM2 inhibitor (Nutlin‐3) and MDM2‐PROTACs (CL144) and examined the protein expression levels of osteogenic differentiation markers RUNX2 and osteocalcin (OCN) using immunostaining and confocal microscopy. For RUNX2, we observed significant nuclear translocation in all treatment groups compared to the OM control, except for Nutlin‐3 at 1 µM (Figure S7, Supporting Information). This observation is consistent with the well‐established role of RUNX2 as a critical transcription factor in osteogenic differentiation. RUNX2 translocation to the nucleus is essential for activating the expression of osteogenic genes.^[^ ^21^ ^]^ For osteocalcin (OCN), we observed a slight increase in intensity in the Nutlin‐3 and CL144 (10 µM) treatment groups; however, there was no statistically significant difference compared to the control group. These findings suggest that while RUNX2 translocation was evident, the downstream activation of late‐stage osteogenic markers like OCN may require additional time to fully manifest (Figure S7, Supporting Information).

### 2.6 Pharmacokinetic Properties of CL144

Before evaluating the in vivo bone regeneration efficacy of CL144 in animal models, we conducted a pharmacokinetic (PK) study in BALB/c mice by intravenous (iv) and oral (po) administration. As shown in **Table** **1**, intravenous administration of CL144 exhibited moderate PK properties, including substantial systemic exposure (*C_max_* = 1630 ng mL^−1^ and AUC_0–t_ = 532 ng mL^−1^), a reasonable half‐life (*T_1/2_* = 1.18 h), and moderate plasma clearance (Cl = 30.6 mL min^−1^ kg^−1^). In contrast, oral administration of CL144 resulted in low plasma exposure with a bioavailability of 4.74%.

**Table 1:** Pharmacokinetic profile of CL144 in BALB/c mice^a)^

Parameter	1.0 mg kg^−1^ (iv)	10 mg kg^−1^ (po)
*T*_1/2_ (h)	1.18 ± 0.11	0.93 ± 0.26
*T*_max_ (h)	0.083 ± 0.00	0.83 ± 0.29
*C*_max_ (ng mL^−1^)	1630 ± 111	106 ± 9
AUC_0–t_ (ng⋅h mL^−1^)	532 ± 35	252 ± 44
AUC_0–∞_ (ng⋅h mL^−1^)	546 ± 39	256 ± 49
MRT_0–t_ (h)	0.28 ± 0.02	1.58 ± 0.36
MRT_0–∞_ (h)	0.42 ± 0.06	1.67 ± 0.46
*V*_ss_ (mL kg^−1^)	774 ± 92	‐
CL (mL min^−1^ kg^−1^)	30.6 ± 2.1	‐
*F* (%)	‐	4.74 ± 0.83

^a)^ Values are shown as the means ± standard deviation of three independent experiments.

### 2.7 Validation of MDM2‐PROTAC Effect on Bone Regeneration in Preclinical Models

**Figure 5:**
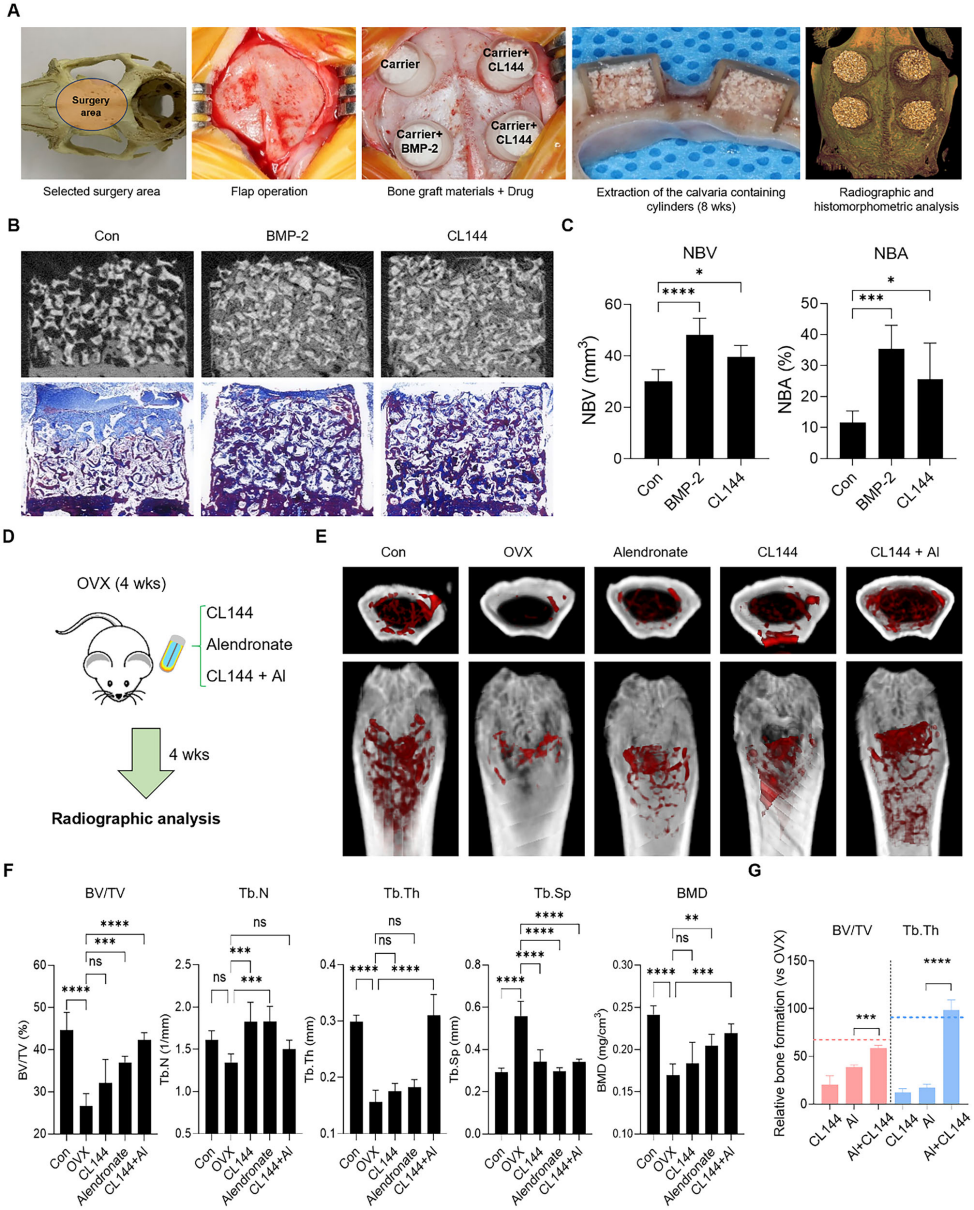
The effects of MDM2‐PROTAC on bone regeneration in preclinical models. A) A process of a vertical onlay graft model on rabbit calvaria. B) Radiographic (upper) and histologic (lower) images of augmented rabbit calvaria bone tissues. C) Quantification of micro‐CT data with parameters of new bone volume (NBV) and new bone area (NBA); ANOVA Bonferroni test: *p < 0.05, ***p < 0.001, ****p < 0.0001; mean with SEM; n = 6. D) Schematic diagram of OVX‐induced osteoporosis model; 4 weeks administration of CL144 or Nutlin‐3 using implanted Alzet pumps in mice induced with osteoporotic conditions through ovariectomy (OVX) surgery. E) The micro‐CT 3D analysis of femur tissues in an OVX‐induced osteoporosis model, following administration of CL144 (0.5 mg kg^−1^) and Al single‐treatment (0.5 mg kg^−1^) or co‐treatment condition (0.5 mg kg^−1^ group); white structure = cortical bone, red structure = trabecular bone, Al = Alendronate. F) The bone formation parameters analysis such as bone volume/tissue volume (BV/TV), trabecular number (Tb.N), trabecular thickness (Tb.Th), and bone mineral density (BMD) following the micro‐CT data analysis, ANOVA Bonferroni test: ns = non‐significant, *p < 0.05, **p <0.005, ****p < 0.0001, Al = Alendronate; mean with SEM; n = 6. G) Compared to the OVX group, relative comparison of bone formation parameters in the CL144, Al single‐treatment group and co‐treatment group; Student's t‐test: ***p < 0.001, ****p < 0.0001; mean with SEM; n = 6; The red dotted line represents the BV/TV value of normal bone (Control), and the blue dotted line represents the Tb.Th value of normal bone (Control).

The local osteogenic effect of MDM2‐PROTAC was verified using a rabbit calvarial vertical onlay graft model. This model is one of the most challenging experimental designs for determining local osteogenic potential because it involves bone regeneration beyond the boundary of pristine bone. Each CL144 (10 μΜ) and recombinant human BMP‐2 (rhBMP‐2) (3.85 μΜ) solutions was soaked onto 0.1 g of synthetic biphasic calcium phosphate carrier for 10 min following clinical guidelines. They were then grafted onto polycarbonate cylinders fixed on the calvaria. After eight weeks, the rabbits were sacrificed and radiological and histological analyses of the cylinders were performed (**Figure** **5**A). All animals showed normal healing processes without significant clinical complications such as weight loss, wound dehiscence, severe swelling, or infection during the experimental period. At eight weeks, the rhBMP‐2 group showed a significantly larger new bone volume than the control group in micro‐CT analysis (48.07 ± 6.66 mm3 versus 30.11 ± 4.62 mm^3^, respectively, *p* < 0.05) (Figure 5B,C). The CL144 group showed higher new bone volume than the control group, however, no significance was found compared to the control and rhBMP‐2 groups (39.69 ± 4.42 mm^3^, *p* < 0.05). Histological analysis revealed massive new bone formation on top of the basal bone in the experimental group. The carrier particles connected to the newly formed bone without any inflammatory reactions. The results of the histomorphometric analysis are presented in Figure 5B,C. Both CL144 and rhBMP‐2 groups had a significantly greater amounts of new bones than the control group at eight weeks (25.58 ± 11.70%, 35.29 ± 7.67% and 11.61 ± 3.76%, respectively) (*p* < 0.05). CL144 showed bone regeneration efficacy similar to that of rhBMP‐2, and no difference in the amount of new bone was observed between the two groups.

Finally, we assessed the regenerative effects of MDM2‐PROTAC after general administration using ovariectomy (OVX)‐induced osteoporosis model. Compounds were administered using an osmotic pump system to provide controlled and sustained release of substances throughout the body (Figure 5D).^[^ ^22^ ^]^ OVX‐induced bone loss occurred over four weeks, and an osmotic pump containing CL144 or Nutlin‐3 was implanted subcutaneously. The pump system was set to deliver the drug at a rate of 0.11 µL h^−1^ for 28 days (Figure S8A, Supporting Information). When CL144 was administered at doses of 0.5 and 1 mg kg^−1^, we observed a notable recovery in trabecular structures in the mouse femur, while Nutlin‐3 group presented lower trabecular density (trabecular structures are labeled by red color) (Figure S8B, Supporting Information). Key parameters linked to bone formation such as bone volume/tissue volume (BV/TV), trabecular bone number (Tb.N), and bone mineral density (BMD) were evaluated. Compared to the OVX group, CL144 resulted in a substantial recovery in BV/TV (65% for CL144 0.5 mg kg^−1^), Tb.N (58% for CL144 0.5 mg kg^−1^) and BMD (12% for CL144 1 mg kg^−1^) (*p* < 0.05). Additionally, when compared to the Nutilin‐3 group, CL144 showed significant improvement in bone formation parameters such as BV/TV (56% for CL144 0.5 mg kg^−1^), Tb.N (61% for CL144 0.5 mg kg^−1^), and BMD (13% for CL144 1 mg kg^−1^) compared to the same concentrations of Nutlin‐3 (Figure S8C, Supporting Information). To analyze the newly formed bone histology, Hematoxylin & Eosin (H&E) and Masson's trichrome (MT) staining were performed on mouse tibia and femur tissues. Compared with the control (Con) group, the OVX group exhibited a significant reduction in trabecular structures, with a scattered and disordered arrangement. In the MT staining, the OVX group showed a loss of collagenous fibers relative to the control groups. H&E staining showed no significant differences between the Nutlin‐3 treatment and OVX groups. However, in the CL144 treatment group, newly formed trabecular structures were observed. In the MT‐stained sections, we observed the recovery of dense collagen structures along with the formation of new trabecular bone in CL144 treated samples (black arrowheads) (Figure S8D, Supporting Information). These preclinical results suggest that sustained treatment with MDM2‐PROTAC effectively improved osteoporotic conditions in the OVX model.

Next, we compared the regenerative effect of CL144 with that of a bisphosphonate reagent (alendronate), a standard medication for osteoporosis therapy, and evaluated the possibility of combinatorial treatment (Figure 5D). In our study, CL144 treatment presented results comparable to those of the alendronate‐treated group, effectively rescuing new bone structures and increasing bone formation parameters (Figure 5E,F). Surprisingly, combined treatment with CL144 and alendronate significantly improved most bone formation parameters, except Tb.N (*p* < 0.005) (Figure 5F). Notably, we observed a synergistic improvement in trabecular structures in the combinatorial treatment group compared with the alendronate single‐treatment group, especially in the parameters of BV/TV and trabecular thickness (Tb. Th) (Figure 5G). While a single treatment with alendronate induced a 38.66% recovery in BV/TV relative to the OVX group, the combination with CL144 remarkably induced a total improvement of 58.61%. Additionally, trabecular thickness increased by 17.07% and 98.43% in the single and combination treatment groups, respectively (*p* < 0.001) (Figure 5G). These data indicated the efficacy of the combinatorial approach of MDM2‐PROTAC and bisphosphonate, which induced an almost complete recovery to the level of normal bone (Figure 5G), thus providing a promising strategy for osteoporosis therapy. To further evaluate the safety profile of CL144 in this combinatorial approach, we assessed its potential to induce bone marrow toxicity. Using immunohistochemistry (IHC), we evaluated the expression of the anti‐apoptotic gene Bcl‐2 and the key apoptosis marker cleaved caspase‐3. The results demonstrated that CL144 treatment effectively reduced MDM2 expression and significantly increased p53 expression compared to the control (Con and DMSO group). Notably, in the group treated with both CL144 and bisphosphonate, p53 expression levels were further elevated compared to the group treated with CL144 alone. Importantly, there was no corresponding increase in the expression of apoptosis markers such as Bcl‐2 or cleaved caspase‐3 (Figure S9, Supporting Information). These findings suggest that CL144 does not induce apoptosis in the bone marrow and is unlikely to cause bone marrow toxicity, further supporting its potential as a safe and effective therapeutic agent in osteoporosis treatment.

## 3 Discussion

In this study, we initially applied a PROTAC system to the field of regenerative medicine, broadening the scope of PROTAC applications. We systematically developed a series of MDM2‐targeting PROTACs and evaluated their degradation efficiency and biological activity to identify the most effective compound for osteogenic differentiation of hBMSCs. Among these, CL144 demonstrated robust osteogenic activity, and we provided evidence of its effectiveness in bone regeneration through multidisciplinary approaches, spanning from cultured cells to animal models.

Based on this evidence, we demonstrate that PROTAC is a promising strategy for bone regeneration.

Compared to traditional approaches for bone regeneration, PROTAC offers several distinct advantages. First, unlike small‐molecule inhibitors, PROTACs facilitate the degradation of target proteins, ensuring a more sustained therapeutic effect by eliminating proteins that negatively regulate bone formation. Thus, a prolonged impact on aberrant signaling proteins may be more efficacious and may mitigate the development of drug resistance, which is commonly observed with other types of inhibitors. Second, PROTACs catalytically promote the ubiquitination of target proteins, leading to their proteolysis, potentially allowing for lower effective dosages and reducing the burden of drug loading for clinical applications. The sustainability and potency of PROTACs effectively decreases the risk of side effects in bone regeneration therapy.^[^ ^7^, ^23^ ^]^ Finally, PROTAC technology can be engineered to target proteins that lack binding sites for conventional inhibitors (undruggable proteins), thereby expanding the range of therapeutic targets for bone‐related diseases.^[^ ^24^ ^]^ Despite these advantages, the clinical application of PROTAC technology in bone regeneration is still in its initial phase, and further research and development are needed to translate these benefits into viable clinical treatments, including studies on their long‐term safety and efficacy in promoting bone regeneration and healing.

Our MDM2‐PROTACs demonstrated remarkable efficacy in degrading the target protein (MDM2), achieving almost complete degradation in a short period. This rapid action was complemented by a high level of maximal degradation, indicating not only the speed but also the extent of its effect (Figure 3F,G). The superior performance of our compound suggests that it could offer a substantial advantage in therapeutic applications, where rapid and complete target degradation is critical. Additionally, MDM2‐PROTAC not only degraded MDM2 but also significantly increased p53 levels, which is a key transcription factor for osteogenic differentiation (Figure 1D,E). These effects were further validated by whole transcriptome analysis, which revealed significant enrichment of GO terms related to the p53 signaling pathway and biomineralization processes in CL144 treated group (Figure 4C). These comprehensive data suggest that MDM2‐PROTAC successfully overcomes the limitations imposed by MDM2‐p53 feedback leading to effective regulation of the MDM2‐p53 signaling pathway.

However, uncontrolled activation of p53 disrupts cell viability. Activated p53 promotes the transcription of proapoptotic genes, leading to mitochondrial disruption, caspase activation, and cell disassembly via apoptosis.^[^ ^25^ ^]^ In our study, MDM2‐PROTAC induced prolonged activation of p53 in hBMSCs during osteogenic differentiation. However, BMSCs did not undergo apoptosis as evidenced by the unchanged proportion of cleaved capase‐3 positive cells after PROTAC treatment (Figure S2, Supporting Information). This finding is distinct from that observed in cancer cells, where robust apoptotic signals are induced by the activation of p53 via MDM2 degradation.^[^ ^18^ ^]^ The reported p53 dynamics could explain these distinct effects depending on the cellular context: sustained p53 levels typically lead to senescence and apoptosis, whereas oscillatory p53 activity selectively activates genes involved in DNA damage repair.^[^ ^26^ ^]^ This suggests that p53 dynamics may have cell type‐specific effects, influencing distinct cellular responses to MDM2 degradation.

Using preclinical models, we conducted a comprehensive study to demonstrate the efficacy of PROTAC in bone regeneration. We observed significant enhancement in multiple bone parameters following both local and systemic administration of MDM2‐PROTAC. Notably, the combination treatment of MDM2‐PROTAC with bisphosphonates almost completely restored osteoporotic conditions to levels comparable to those in normal bone (Figure 5G and Figure S8, Supporting Information). This synergistic effect may be due to the dual strategy of targeting key cell types involved in bone remodeling: osteoblasts and osteoclasts. The combined effect of osteoblast differentiation induced by MDM2‐PROTAC and osteoclast inhibition by bisphosphonates could shift the balance of bone remodeling toward the formation phase. These preclinical results and mechanistic insights suggest the potential for developing a novel therapeutic module for osteoporosis.

PROTACs have been primarily used for the targeted degradation of oncogenic proteins in cancer. To the best of our knowledge, this is the first example of a PROTAC application in the field of regenerative medicine. Our study extends their application to tissue regeneration, with significant results in promoting bone regeneration using MDM2‐PROTAC. Based on these results, we anticipate that a new therapeutic modality for hard tissue regeneration may be developed using an expanded spectrum of PROTAC systems.

## 4 Experimental Section

### Cell Culture, Osteogenic Differentiation, and Transient Transfection

Human Bone Marrow‐derived Mesenchymal Stem Cells (hBMSCs) were provided by Professor Lee's laboratory, and cells from passages four to nine were used for all experiments. hBMSCs were cultured in alpha‐minimum essential medium (Welgene, LM008‐01) supplemented with 10% Fetal Bovine Serum (FBS) (Gibco, US origin, 16000–044) and 1% penicillin/streptomycin (Gibco, 15140‐122).

To induce osteogenic differentiation, the cells were seeded in confluent plates and cultured in osteogenic induction medium the following day. The differentiation medium was prepared by adding 10 mM β‐glycerophosphate (Sigma‐Aldrich, G9422), 50 µM ascorbic acid (Sigma‐Aldrich, A4403), and 0.1 µM dexamethasone (Sigma‐Aldrich, D4902) to the culture medium. The cells were treated with MDM2 inhibitors, Nutlin‐3 (Sigma‐Aldrich, N6787) or CL144 in an osteoinductive medium and incubated for 10–14 days to induce osteogenic differentiation. The differentiation medium was changed every 2–3 days (The list of MDM2 inhibitors used is shown in Figure 1A and presented in Table S1, Supporting Information).

### HeLa Cells

HeLa cells were cultured in DMEM (Welgene, LM001‐05) supplemented with 10% FBS (Welgene, S001‐07). For transient transfection, the cells were seeded at ≈70% confluence. The following day, transfection was performed using Lipofectamine 3000 (Invitrogen, L3000015) for 24 h according to the manufacturer's protocol. The cells were transfected with 100 ng of EGFP‐MDM2 per well in the 96‐well plate and were transfected with 2 µg of MDM2 WT and 2 µg of HA‐ubiquitin, total 4 µg per well in the 6‐well plate.

### Alizarin Red S Staining

Following osteogenic differentiation for the indicated number of days, the cells were fixed in 70% ethanol for 3 min at room temperature or in 4% PFA (Tech & Innovation, BPP‐9004) for 30 min at room temperature, washed with distilled water, and stained with 2% Alizarin Red S (Sigma‐Aldrich; pH 4.2, A5533) for 30 min.

### Plasmid DNA Construct Manufacture

To construct the EGFP‐MDM2 plasmid DNA, MDM2 WT was amplified by PCR using specific primers. The PCR products were digested with BamH1 and EcoR1 to generate compatible ends. The plasmid backbone, EGFP‐C1, previously digested with the same enzyme, was ligated into the digested PCR product using T4 ligase (Biolabs, M0202S). The resulting plasmid construct was confirmed using restriction enzyme digestion and DNA sequencing. MDM2 WT (#16233) and HA‐ubiquitin (#18712) were purchased from Addgene (used primer sequences, see Table S2, Supporting Information).

### Calcium Deposit Staining

hBMSCs were differentiated in the presence of the 10 mM β‐glycerophosphate (Sigma‐Aldrich, G9422) and 50 µM ascorbic acid (Sigma‐Aldrich, A4403) in culture medium for 10 d. Subsequently, the cells were fixed with 4% PFA for 30 min, followed by two washes with Mg^2+^‐and Ca^2+^‐free dPBS (Welgene, LB‐204). To stain for calcium, X‐Rhod‐1 (Invitrogen, X14210) dye was diluted in Mg^2+^‐and Ca^2+^‐free dPBS at a concentration of 1 µM and incubated at room temperature in the dark for 1 h. The cells were then washed five times with Mg^2+^‐and Ca^2+^‐free dPBS at 10 min intervals. The samples were observed under a Cytation5 microscope (Agilent Technologies) at a wavelength of 595 nm.

### Western Blot

After discarding the medium and washing the cells twice with cold dPBS, the cells were collected using a scraper and lysed using RIPA buffer (GenDEPOT, R4200‐010) containing protease and phosphatase inhibitors (GenDEPOT, P3200‐001). The collected samples were centrifuged at 13 200 rpm for 10 min at 4 °C, and the supernatant containing total protein was collected. SDS‐PAGE gels (10–12%) were prepared, and protein samples were mixed with 5X loading buffer containing reducing agent (Biosesang, S2002) and heated at 98 °C for 5 min. Equal amounts of protein samples were loaded onto a gel, separated by electrophoresis, and transferred onto nitrocellulose membranes using a Dry Transfer Stacks Kit (Invitrogen, IB23001). The membrane was blocked with 5% nonfat milk in 1X TBST for 1 h at room temperature to prevent nonspecific binding. The membranes were incubated with primary antibodies specific to the target proteins overnight at 4 °C with gentle shaking. The next day, the membrane was washed three times with TBST at 10 min intervals to remove unbound primary antibodies. The membranes were incubated with secondary antibodies (anti‐rabbit and anti‐mouse) conjugated to horseradish peroxidase (HRP) for 1 h at room temperature. The membrane was washed three times with 1X TBST at 10 min intervals to remove unbound secondary antibodies. After washing, a chemiluminescent substrate (ECL, Millipore, WBULS0100) was applied to the membrane to visualize protein bands using a chemiluminescence detector (for the antibodies used, see Table S2, Supporting Information).

### Immunoprecipitation

Hela cells (passage. 5) 7 × 10^5^ cells well^−1^ were seeded in a 6‐well plate and allowed to adhere overnight (o/n). The next day, WT MDM2 and HA‐ubiquitin plasmid transfection was performed using Lipofectamine 3000 for 24 h, according to the manufacturer's instructions. After transfection for 24 h, cells were treated with CL144 for different time intervals, including 15, 30, and 60 min. After drug treatment, the cells were lysed using immunoprecipitation (IP) lysis buffer (ThermoFisher, 87788) supplemented with a protease inhibitor cocktail (PIC). The cell lysates were harvested for subsequent experiments. The cell lysate was incubated with a HA‐tag primary antibody (1′Ab) at 4 °C overnight to capture the target protein complexes. The next day, the antibody–protein complexes were bound to the beads for 1 h. The beads were washed three times with IP lysis buffer to remove nonspecific binding. The supernatant was carefully aspirated, and the remaining protein complexes on the beads were eluted by adding 1X sample buffer and boiling at 98 °C for 5 min. Eluted proteins were used for western blot analysis.

### Live Cell Imaging and Fixed Fluorescence Imaging

Real‐time Live cell imaging (phase contrast, EGFP fluorescence) was performed to monitor EGFP‐MDM2 intensity at 7 min intervals after compound treatment using Cytation5 (Agilent) laser autofocus conditions to capture EGFP fluorescence to assess intensity. For fluorescence imaging, the cells were fixed using 4% PFA and washed three times with 1X dPBS. Fluorescence imaging was performed using the Cytation5. The intensity of fluorescence was assessed by tracking the fluorescence of individual cells in each image using the Time‐lapse analyzer or ROI measure feature of the Fiji ImageJ software (Version 1.54 h, USA).

### DC_50_, D_max_ Calculation

The *DC_50_* and *D_max_* values were calculated by western blot band quantification according to compound concentrations. The quantification value was normalized to the value relative to the untreated group and calculated using the sigmoidal dose‐response (variable slope) equation in GraphPad Prism.

### Quantitative PCR Analysis

Total RNA was extracted from hBMSCs treated with Nutlin‐3 or CL144, as well as from control cells, using Trizol (Invitrogen, 15596026), according to the manufacturer's instructions. cDNA was synthesized using TOPscript RT DryMIX (Enzynomics, RT200). Human gene‐specific primer sequences were used. (Used primer sequences see Table S2, Supporting Information). qPCR was conducted using the SYBR Green qPCR Master Mix (Applied biosystems, 4 309 155). Relative gene expression levels were calculated using the ΔΔCt method with GAPDH as the internal control for normalization.

### Protein Expression and Purification

E. coli BL21 (DE3) competent cells transformed with pSPEL870 were used to express MDM2 protein.^[^ ^27^ ^]^ The recombinant strain was cultured in 500 mL 2XYT at 37 °C until the optical density (*OD_600_*) reached 0.5. Protein expression was induced by adding 0.2 mM isopropyl β‐D‐1‐thiogalactopyranoside (IPTG; Bioshop, Canada) at 20 °C overnight. The cells were harvested by centrifugation for 15 min at 9300 x g at 4 °C. The protein was purified using Ni‐NTA resin (Qiagen, Germany), according to the manufacturer's protocol. The purified protein was buffer exchanged with phosphate‐buffered saline (PBS, pH 7.4) using a centrifugal filter unit (MWCO: 10 K; Merck Millipore, USA). The protein was stored at –20 °C in PBS with 20% glycerol and 1 mM dithiothreitol (DTT).

### Microscale Thermophoresis Assay

The His‐Tag MDM2 protein was labeled using a His‐Tag Labeling Kit RED‐tris‐NTA second Generation (NanoTemper, MO‐L018) according to the manufacturer's instructions. Equal volumes of MDM2 (200 nM) in PBS‐T buffer (1X PBS, 6.0 mM DTT, 0.05% Tween 20, pH = 7.6) and of RED‐tris‐NTA dye (100 nM) in PBS‐T buffer (1X PBS, 0.05% Tween 20, pH = 7.6) were mixed and incubated for 30 min at room temperature. The mixture was centrifuged for 10 min at 15 000 × g at 4 °C. The supernatant was used for MST assays. The labeled protein (6.0 µL) was mixed with the ligand at serial concentrations in PBS‐T buffer (1X PBS, 0.05% Tween 20, 2% DMSO, pH = 7.6; 6.0 µL). The final mixtures were transferred to capillaries (NanoTemper, MO‐K022) and the samples were measured using a Monolith NT.115Pico (NanoTemper) instrument (auto LED power; medium MST power). The assay was performed in triplicate. The signals were represented as normalized changes in fluorescence (*F_norm_*) upon ligand binding. Baseline‐corrected normalized fluorescence (*DF_norm_*) was obtained from *F_norm_* values using MO Affinity Analysis, and the dissociation constants (*K_d_*) were determined by curve fitting (variable slope model) using GraphPad Prism 10.

### Computational Method

A protocol for predicting the structure of the PROTAC‐induced ternary complex was established to determine the CRBN–PROTAC–MDM2 ternary structure. The protocol consists of three steps. The first step involved predicting the 3D complex between MDM2 and the MDM2 ligand Nutlin‐3a. Second, a linker molecule was attached to the MDM2‐bound ligand, and the conformation of the linker molecule was optimized. The third step involved attaching the E3 ligase ligand, thalidomide, to the end of the linker and positioning the E3 ligase protein, CRBN, according to ligand coordination. During this process, the binding pose of the E3 ligase ligand was varied to generate candidate ternary structures. Finally, molecular dynamics (MD) simulations were performed to simulate the binding process between MDM2 and CRBN facilitated by PROTAC. The final ternary structures were selected on the basis of their binding energies.

The crystal structures of MDM2 in complex with Nutlin‐3a and CRBN in complex with (*S*)‐thalidomide were obtained from the RCSB Protein Data Bank website (https://www.rcsb.org) using the PDB IDs 4HG7 and 6BN7, respectively. All MD simulations were performed using GROMACS software.^[^ ^28^ ^]^ The CHARMM36 m force field^[^ ^29^ ^]^ was employed for the proteins and the TIP3P water model^[^ ^30^ ^]^ was used to describe water molecules. The force field parameters for the PROTAC molecules were generated using the CHARMM general force field (CGenFF) program.^[^ ^30^, ^31^ ^]^ The CRBN–PROTAC–MDM2 ternary structures were solvated in a cubic box with water models extending 10 Å from the ternary structures. The systems were neutralized using Na^+^/Cl^−^ ions by replacing the water molecules. The NPT ensemble was employed in all the MD simulations. The Particle‐Mesh‐Ewald (PME) algorithm was employed for calculating electrostatic interactions with a space cutoff of 12 Å, and the SHAKE algorithm^[^ ^32^ ^]^ was used to constrain hydrogen atoms. Each simulation system was relaxed through a 10 000‐step energy minimization process using the steepest descent algorithm. Subsequently, each system was run for 1000 ns.

### Proteomics Analysis

‐In‐solution protein digestion and TMT labeling: Cells were suspended in PBS containing a cocktail of protease inhibitors (Thermo Fisher, 78 429) and lysed using sonication on ice. The cell lysate samples were combined with 100 µL of 8 M urea in 3 K filter units (Millipore, UFC500396) and centrifuged at 14 000 × g for 15 min. This process was repeated once more. After obtaining the supernatants, dithiothreitol (10 mM) was added to the filter units and incubated at 37 °C for 40 min. Next, iodoacetamide (50 mM) was added to the filter units and allowed to incubate for 20 min in the dark. A final buffer exchange was carried out by adding 100 µL of ammonium bicarbonate buffer (50 mM), followed by another 14 000 × g spin for 15 min. After two further buffer exchanges, the samples were digested with trypsin (Promega, V5280) overnight at 37 °C. The digestion was stopped by adding 1% formic acid, and the samples were desalted using C18 cartridges (Waters, WAT054955). The C18 cartridges were preconditioned with acetonitrile and then equilibrated using 0.1% formic acid. The peptides that adhered to the C18 cartridges were rinsed with 0.1% formic acid and eluted using an elution buffer made of 70% acetonitrile and 0.1% formic acid. The digested peptides were resuspended in 100 µL of triethylammonium bicarbonate buffer (100 mM). Each sample was mixed with a separate TMT (Thermo Scientific, 90110) label suspended in 41 µL of acetonitrile. The samples were incubated at room temperature for 1 h. The labeling reaction was quenched by adding 8 µL of 5% hydroxylamine for 15 min. The samples were then pooled together and dried using SpeedVac. The labeled peptides were resuspended in 300 µL of 0.1% formic acid and desalted using C18 cartridges following standard protocols. The C18 cartridges were conditioned with acetonitrile and equilibrated with 0.1% formic acid. The peptides retained on the C18 cartridges were washed with 0.1% formic acid and eluted with an elution buffer composed of 70% acetonitrile and 0.1% formic acid.

‐Peptide fractionation: For fractionation, pooled sample was loaded to Ultimate 3000 HPLC (Thermo Fisher Scientific) with ACQUITY UPLC Peptide CSHTM C18 column (130Å, 1,7 µm, 1 mm x 1500 mm, Waters). The labeled peptide was separated into 12 fractions with a total 110 min rum time. The linear gradient of buffer B was set as followed, 1% at 0 min, 3% at 1 min, 10% at 20 min, 45% at 90 min, 90% at 94 min, 90% at 103 min, 1% at 105 min, and 1% at 110 min. The mobile phase composition was prepared by buffer A for 10 mM ABC and buffer B for 10 mM ABC in 90% MeCN. All fractions were dried and stored at −80 °C until further use.

‐LC‐MS/MS Analysis: The fractionated peptides were reconstituted to 0.1% formic acid (FA) and analyzed using Orbitrap Exploris 480 (Thermo Fisher Scientific) coupled with Ultimate 3000 UPLC (Thermo Fisher Scientific). In a total of 200 min of analysis, flow rate was set to 0.25 µL min^−1^, and the linear gradient of buffer B was set as followed, 2% at 0 min, 2% at 5 min, 16% at 10 min, 30% at 125 min, 40% at 160 min, 95% at 162 min, 95% at 180 min, 2% at 185 min, and 2% at 200 min. The mobile phase composition was prepared by buffer A for 0.1% FA, 5% dimethyl sulfoxide (DMSO), and buffer B for 0.1% FA, 5% dimethyl sulfoxide (DMSO) in 80% MeCN. To separate the peptide, trap column (2 µm, 2 cm × 75 µm, Thermo Fisher Scientific) and an analytical column (2 µm, 75 µm × 500 mm, Thermo Fisher Scientific) were used. Peptides were analyzed using data‐dependent acquisition (DDA) method. Resolution was set to 60 000 for MS1 and 30 000 for MS2. Scan range was set to 350–1800 m z^−1^. Normalized Collision Energy (NCE) was set to 32% and dynamic exclusion was set to 30 s.

‐Database Search and Data Processing: The LC‐MS/MS raw files were processed with inhouse SAGE open‐source search engine. The human protein database was downloaded from Uniprot (Homo Sapiens, UP000005640). Cysteine alkylation (+57.0214 Da), methionine oxidation (+15.9949 Da), and N termini, lysine TMT (+229.163 Da) are considered as peptide modifications. False discovery rate (FDR) is applied at 1% each at the spectrum, peptide, and protein levels. Using the abundance data, we processed statistical analysis and extracted differentially expressed proteins (DEPs) were defined as permutation‐based *p*‐values<0.001 and |log_2_(Fold Change)| < 0.35 as criteria. Detailed processing python code is freely accessible in following Google Colab url (https://colab.research.google.com/drive/1aPDzZOMi62iDdn6Ffjgxww7JkN5t7owZ?usp = drive_link).

### Bulk RNA‐seq of Cultured Cell and Gene Ontology analysis

‐Sample Preparation: In accordance with the manufacturer's instructions, total RNA was extracted using Trizol from hBMSCs treated with Nutlin‐3 or CL144 as well as from control cells. Utilizing an Agilent Bioanalyzer RNA ScreenTape kit (Agilent, 5067–5576), the integrity of the RNA (RIN) was verified. All samples with RIN value > 8.0 were used for sequencing.

‐Library Construction: RNA‐seq libraries were constructed according to the manufacturer's instructions for the SRSLY Library Prep kit, which were provided by Claretbio (CBS‐K155B‐24). In conclusion, both first‐ and second‐strand cDNA synthesis procedures employed the SuperScript III First‐Strand Synthesis System (Invitrogen, 18 080 051) and E. coli DNA polymerase 1 (Enzynomics, DP002S). This work fragmented and enriched the mRNA using oligo (dT). After ligating the cDNA fragments to adapters, PCR amplification was performed.

‐Sequencing: The libraries were performed to sequencing on the Nextseq2000 (Illumina) platform, utilizing the SRSLY RNA NanoPlus kit to generate 100 bp paired‐end reads.

‐Data Analysis: FastQC was used to trim the adaptor sequences and remove low‐quality reads from the raw sequencing data (FASTQ files). After that, Hisat2 v2.2.1 was used to align these clean reads with the Human Genome Reference GRCh38. DESeq2 was used for differential expression analysis, while g: Profiler was used for GO enrichment analysis.

### Pharmacokinetic Study

Pharmacokinetic (PK) studies of CL144 were conducted by Medicilon Preclinical Research (Shanghai) LLC. Six BALB/c mice were divided into two groups, and CL144 was administered by tail vein injection (iv; 1.0 mg kg^−1^) or gavage (po; 10 mg kg^−1^). Blood samples were collected from the submandibular vein at 0.083, 0.25, 0.5, 1, 4, and 24 h for the iv group and at 0.25, 0.5, 1, 3, 6, and 24 h for the po group. Blood samples were centrifuged at 6800 x g for 6 min at 2–8 °C within 1 h after collection and stored at ≈−80 °C. Plasma samples were analyzed by LC‐MS/MS (TQ6500+), and PK parameters were calculated using the FDA‐certified pharmacokinetic program Phoenix WinNonlin 7.0 (Pharsight, USA).

### Vertical Onlay Graft Model in Rabbit Calvaria

‐Establishment of Vertical Onlay Graft Rabbit Model: The experimental protocol was approved by the Institutional Animal Care and Use Committee of Yonsei Medical Center, Seoul, Korea (IACUC Approval No. 2024–0146). Total six male, New Zealand white rabbits, weighing ≈2.8–3.2 kg were used for the rabbit calvaria onlay graft model. Rabbits were kept in separate cages under standard laboratory conditions with free access to food and water and provided standard meals according to the ARRIVE guidelines. The animals were sacrificed eight weeks postoperatively (n = 6), and each rabbit was divided into three different groups. Commercially available rhBMP‐2 were used for the positive control (Cowellmedi, Korea). CL144 and BMP‐2 solutions (200 µg) were soaked onto 0.1 g a biphasic calcium phosphate carrier (BCP; Osteon III; Genoss, Korea) for 10 min before grafting. The groups were as follows: 1) CL144 group: 10 μΜ CL144 soaked BCP; 2) BMP group: 3.85 μΜ rhBMP‐2 soaked BCP; and 3) BCP group: carriers only. This surgical procedure has been described previously.^[^ ^33^ ^]^ General anesthesia was induced by injecting 65 mg kg^−1^ ketamine (Ketalar, Yuhan, Korea) and xylazine (Rompun, Bayer, Korea). A full‐thickness flap was raised under local anesthesia with 2% lidocaine and 1:100 000 epinephrine to expose the calvarial bone. Slits around the sagittal suture were prepared using a trephine bur (7 mm in diameter, 1 mm in depth), and then four perforations of the external cortical bone plate were made. Polycarbonate cylinders (7 mm in outer diameter and 5 mm in height) were screwed into each slit and then filled with randomly assigned materials. The plastic cylinders were covered with lids, and primary wound closure was performed using absorbable 6‐0 suture material (Monosyn, B. Braun Surgical, S.A, Rubicon, Spain). After eight weeks of healing, the calvaria‐containing cylinders were extracted for radiographic and histomorphometric analyses.

‐Radiographic analysis: A single‐blinded examiner conducted all the measurements. Volumetric assessments were performed on harvested specimens using micro‐CT (SkyScan1173; SKYSCAN, Kartuizersweg 3B2550, Kontich, Belgium) prior to histological preparation. Digital images were captured at 130 kVp and 60 µA with 1.0‐mm aluminum filtration. The specimens were exposed to radiation at 500 ms per 0.2‐degree rotation. High‐resolution images were obtained with a pixel size of 14.91 µm. For image reconstruction, 2240 × 2240 pixel images were processed using computer software (Nrecon, Bruker‐CT, ver.1.5.1.2, Kontich, Belgium). Another software (Ct Analyzer, Bruker‐CT, ver.1.14.4.1) was employed to segment the bone trabecular pattern and marrow cavity for bony structure analysis. The volume of the newly formed bone within the cylinders was measured.

‐Histologic analysis: Specimens were decalcified in Calci‐Clear Rapid (National Diagnostics, 305 Patton Drive, USA) for two weeks. The embedding paraffin blocks were then sectioned into 4 µm thick slices and stained with Masson trichrome. Histomorphometric measurements were conducted by an experienced and blinded examiner using Photoshop CS software version.21.2.2, North America). Within the cylinder, the total augmented area, area and proportion of newly formed bone, and residual graft materials were measured as previously described.^[^ ^34^ ^]^

### Ovariectomized Mice Model

‐Establishment of Ovariectomized Mice Model: The experimental protocols were approved by the IACUC (Approval code: IACUC230118) at the CHA Laboratory Animal Research Center. The mice were handled in compliance with the Guidelines for the Care and Use of Animals at the institution. Female C57BL6 mice (seven weeks old (n = 57)) were randomly divided into six groups (n = 8–10): Con + Vehicle, OVX + Vehicle, OVX + Nutlin‐3 (0.5 and 1 mg kg^−1^), OVX + CL144 (0.5 and 1 mg kg^−1^). All surgeries and pump implantations were performed under aseptic conditions. Mice were anesthetized before removing both sides of the ovaries. In the fourth week following surgery, an Alzet osmotic pump (Durect Corp, #1004) was implanted in the mice. Osmotic pumps containing Nutlin‐3 and CL144 (0.5, 1 mg kg^−1^) were implanted for 28 days delivery at 0.11 µl/h subcutaneously between the scapulae via a small incision.

‐Micro‐CT analysis: Micro‐CT images of mouse femurs fixed in 10% neutral‐buffered formalin solution were obtained using a high‐resolution Skyscan 1173 micro‐CT system (Bruker, Aartselaar, Belgium). Images were acquired at an effective pixel size of 50 µm (130 kV and 60 µA radiation source; 1.0 mm aluminum filter). Subsequently, 3D images were reconstructed from the 2D X‐ray projections by implementing the Feldkamp algorithm, and appropriate image corrections, including ring artifact correction, beam hardening correction, and fine‐tuning, were processed using NRecon software (SkyScan 1173, Belgium). The dynamic image range (contrast limits) was determined at 0–0.3 in units of attenuation coefficient and was applied to all datasets for optimum image contrast. After acquisition and reconstruction of the datasets, images were first reoriented on each 3D plane using DataViewer software (SkyScan 1173, Belgium) to align the long axis of the femur parallel to the coronal and sagittal planes. Next, 3D morphometric analyses of the distal femur and body of the lumbar vertebrae were performed using CT‐Analyzer software (SkyScan 1173, Belgium). Regarding the region of interest (ROI), this work analyzed the 1.5 mm heighted trabecular bone of the distal femur metaphysis, excluding the growth plate. The ROIs were delineated using a freehand drawing tool while maintaining a 2240‐pixel clearance from the endosteal surface. A clearance of 0.1 mm was maintained from the growth plate. A global threshold of 60 (1.01573 g cm^−3^) was applied to all scans to extract a physiologically accurate representation of the trabecular bone phase. Morphometric parameters were then computed from the binarized images using direct 3D techniques (marching cubes and sphere‐fitting methods), and percent bone volume (BV/TV, %), and trabecular number (Tb.N, mm^−1^). All quantitative and structural parameters followed the nomenclature and units recommended by the American Society for Bone and Mineral Research (ASBMR) Histomorphometry Nomenclature Committee. After data quantification,^[^ ^26b^ ^]^ 3D rendered images were generated to visualize the analyzed regions using the marching cube method.

‐H&E staining: Following decalcification, the specimens were fixed in paraffin and longitudinally sliced to a thickness of 3 µm using a microtome. Subsequently, each sample was affixed to a slide and exposed to a temperature of 55 °C. The samples were deparaffinized through four 5 min changes in xylene, followed by dehydration in sequentially decreasing alcohol solutions (100%, 95%, 80%, and 70%) at 2 min intervals for each change. The slides underwent a 1 min wash in flowing purified water and immersed in Harris hematoxylin‐I for 5 min, followed by a 1 min wash under flowing tap water. Sections were briefly immersed in ammonia water for 30 s and washed with tap water for 3 min. Subsequently, the sections were stained with Eosin Y for 2 min and 30 s, followed by gradual drying in 95%–100% ethanol. Any residual stain was rinsed with distilled water and each section was mounted.

‐Masson's trichrome staining: Following deparaffinization and hydration, the sections underwent treatment with a mordant containing a 5% iron alum solution in a dry oven at 56 °C for 30 min. Sections were washed under running tap water for 5–6 min to remove picric acid. Sections were stained with Weigert's iron hematoxylin solution for 10 min, followed by rinsing with purified water. A 5 min staining with Biebrich scarlet‐acid fuchsin solution ensued, succeeded by washing with purified water. The sections were immersed in a phosphomolybdic‐phosphotungstic acid solution for 5 min. After discarding the solution, the sections were washed with distilled water. Differentiation was performed in 1% glacial acetic acid solution for 3 min. The solution was discarded, and the remaining stain was washed with distilled water.

‐Immunohistochemistry: Decalcified femurs were fixed in 4% paraformaldehyde (PFA) at 4 °C for 24 h, rinsed three times with phosphate‐buffered saline (PBS), and decalcified in 12.5 mM EDTA (pH 8.0) for 14 days. The specimens were dehydrated through a graded ethanol series (70%, 80%, 95%, 100%) and embedded in paraffin. Longitudinal sections (3 µm thick) were prepared using a microtome, mounted on glass slides, and dried at 55 °C. For deparaffinization, slides were treated with xylene (four 5‐min treatments) and rehydrated through descending ethanol concentrations (100%, 95%, 80%, 70%) in 2‐min intervals. Immunohistochemistry was performed with a ready‐to‐use IHC/ICC kit (BioVision, Inc.). Sections were incubated overnight at room temperature with primary antibodies (1:100) against MDM2, p53, Bcl‐2, and cleaved caspase‐3, followed by a 20‐min incubation with HRP‐conjugated anti‐mouse IgG polymer. After PBS washes, antigen‐antibody complexes were visualized using 3,3′‐diaminobenzidine (DAB) for 10 min, and sections were counterstained with hematoxylin for 1 minute. The stained sections were imaged using a Slide Scanner (Zeiss, Germany).

### Data Statistical Analysis

All statistical analyses were performed using GraphPad Prism version 10.1.2. Descriptive statistics, such as the mean with Standard Error of the Mean (SEM), were calculated for continuous variables. Group comparisons were performed using a one‐way ANOVA or Student's *t‐*test, followed by Bonferroni multiple comparison tests. A *p*‐value < 0.05 was considered statistically significant, and the results are presented as mean with SEM.

## Conflict of Interest

The authors declare no conflict of interest.

## Author Contributions

S.J., J.‐K.C., and W.A. contributed equally to this work. Conceptualization: J.M.K., S.J., S.L., H.J.A., J.C., S.Y.K. Methodology: J.M.K., S.J., S.L., H.J.A., J.C., J.W.K., M.S.K., J.K.C., K.T.H., J.S.L. Investigation: J.M.K., S.J., S.L., J.M.L., S.C.A., J.C., W.A., J.W.K., W.J.C., S.J.C., T.H.Y., M.S.K., J.K.C., J.M.C. Visualization: J.M.K., S.J., S.L., S.C.A., J.C., J.W.K. Supervision: J.M.K., S.L., J.C., S.Y.K. Writing—original draft: J.M.K., S.J., S.L., S.C.A., J.C., J.W.K., J.K.C. Writing—review & editing: J.M.K., S.J., S.L., H.J.A., J.C., J.W.K.

## Supporting information



Supporting Information

Supporting Information

## Data Availability

The data that support the findings of this study are openly available in Gene Expression Omnibus (GEO) at https://www.ncbi.nlm.nih.gov/geo/query/acc.cgi?acc=GSE255297, reference number 255297.
