# Supplementary material for: Development of MDM2‐Targeting PROTAC for Advancing Bone Regeneration
Source: Adv Sci (Weinh). 2025 Mar 24;12(19):2415626. doi: 10.1002/advs.202415626 (PMC12097015; doi:10.1002/advs.202415626)
Supplement: Supplementary file 1 — Supporting Information [file ADVS-12-2415626-s001.docx]

Supporting Information

Title: Development of MDM2-targeting PROTAC for advancing bone regeneration

Sol Jeong^1†^, Jae-Kook Cha^2,3†^, Wasim Ahmed^4†^, Jaewan Kim^4^, Minsup Kim^5^, Kyung Tae Hong^6^, Wonji Choi^4^, Sunjoo Choi^4^, Tae Hyeon Yo^4^, Hyun‑Ju An^7^, Seung Chan An^7^, Jaemin Lee^7^, Jimin Choi^2^, Sun-Young Kim^8^, Jun-Seok Lee^6^, Soonchul Lee^7^*, Junwon Choi^6,9^*, Jin Man Kim^1,10,11^*

This file includes:

Figs. S1 to S9

Tables S1 to S3


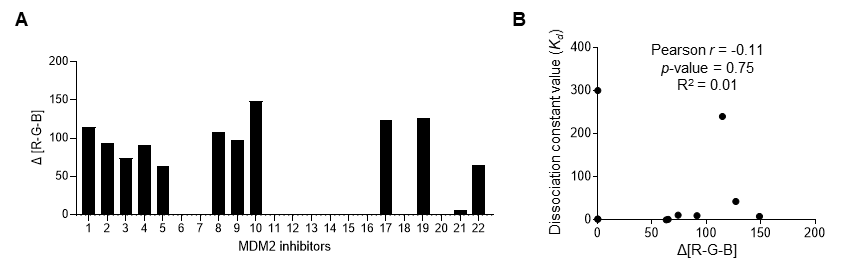


Figure S1. Quantification of osteogenic potency of MDM2 inhibitors and its correlation with binding affinity

(A) Quantification of ARS staining result with MDM2 inhibitors treated human bone marrow-derived stem cells (hBMSCs). (B) Pearson correlation analysis between dissociation constant (*K_d_*) values and Quantified ARS Staining; Dissociation constant lists of compounds is given Table S3.


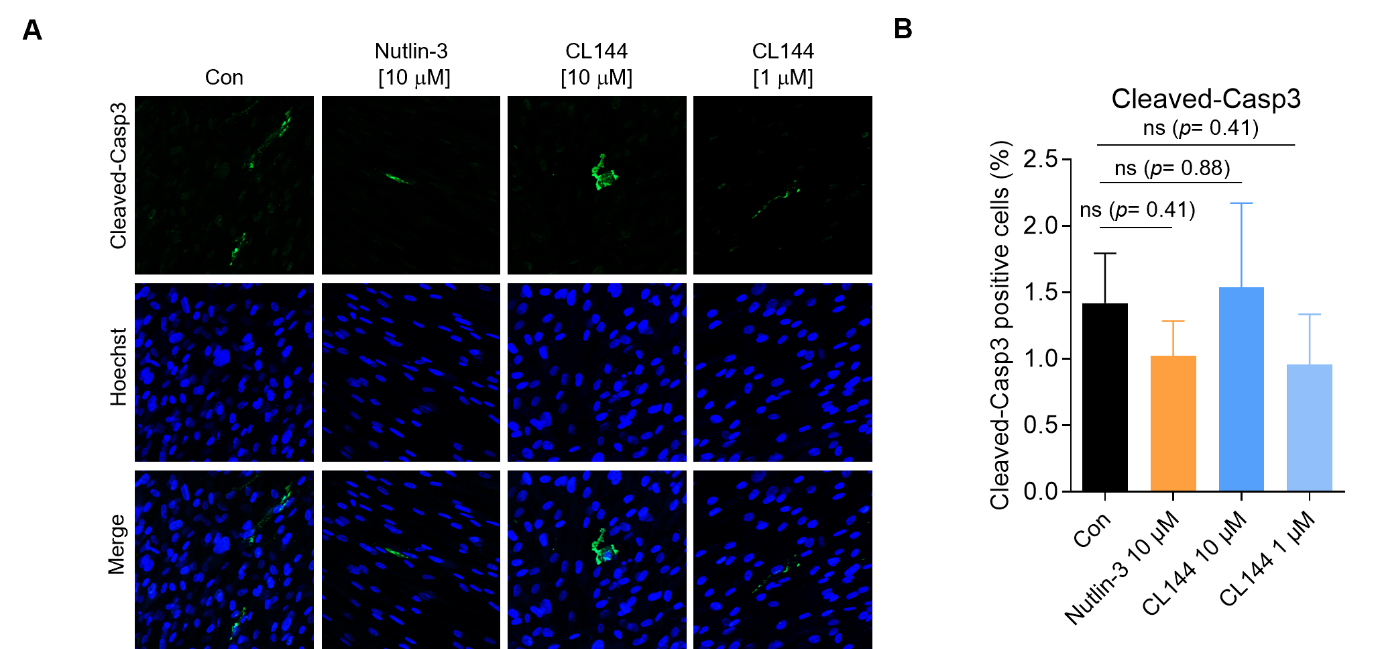


Figure S2. Apoptosis test of MDM2-targeting compounds.

(A) Immunofluorescence images of cleaved-caspase-3 in MDM2 inhibitor (Nutlin-3) and our MDM2-PROTAC (CL144) treated BMSCs. (B) Proportion of the cleaved-caspase-3 positive cells; Student's t-test: ns = non-significant; mean with SEM; n=5.


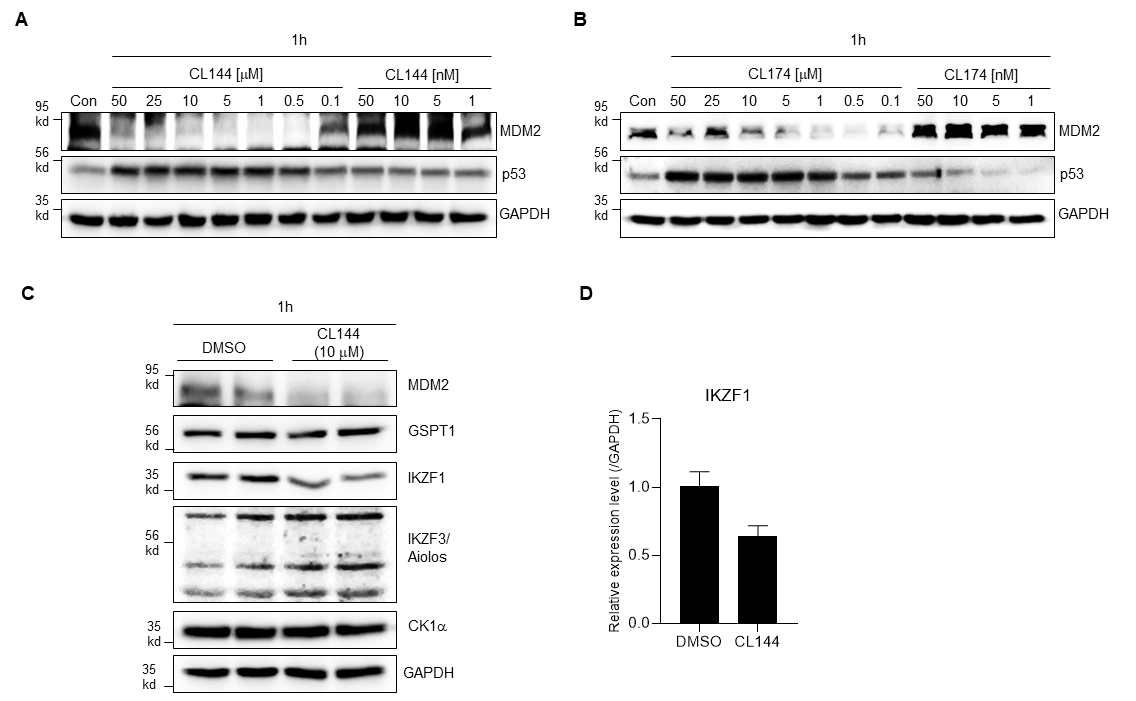


Figure S3. Evaluating the degradation window and Off-target profiles of developed MDM2 targeting PROTACs

(A, B) Immunoblot image of MDM2 and p53 protein in the concentration range of 1 nM-50 mM for CL144 and CL174 in hBMSCs; GAPDH was used as loading control. (C) Neo-substrate immunoblot assay under the condition of 10 μM CL144 (MDM2-PROTAC treated human bone marrow-derived stem cells (hBMSCs). (D) Quantitative analysis of IKZF1 from the immunoblot in (C).


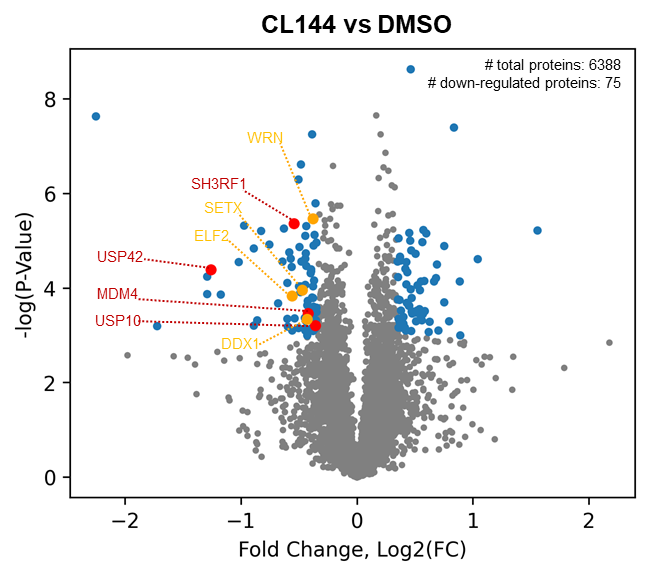


Figure S4. Proteomics analysis of MDM2-PROTAC (CL144) in hBMSCs.

Volcano plot showing protein abundance (Log2(Fold Change)) as a function of *p*-values [−log10(*p*-value)]. Blue: differentially expressed proteins; Red: MDM2–p53 network and ubiquitin processing-related proteins; Yellow: transcription-related proteins.


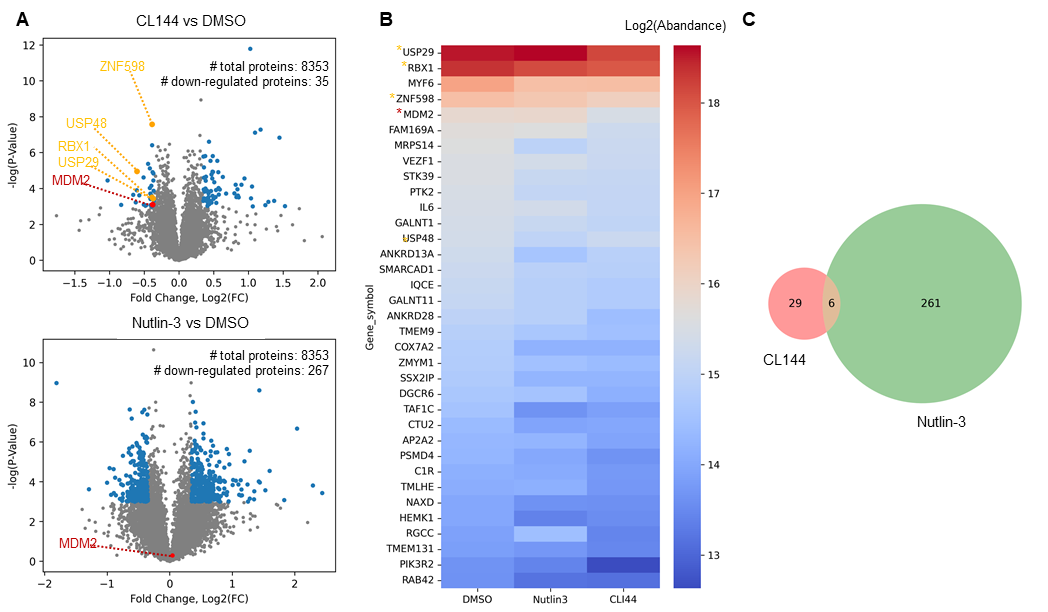


Figure S5. Proteomics analysis of MDM2-PROTAC (CL144) and Nutlin-3 in MDM2-overexpressing HeLa Cells.

(A) Volcano plots showing protein abundance changes [Log2(Fold Change)] as a function of *p*-values [−log10(*p*-value)] of the proteomic analysis of cells treated with CL144 or Nutlin-3. Red: MDM2−p53 network and ubiquitin processing-related proteins; Yellow: transcription-related proteins. (B) Heat map showing protein abundance changes in each group, DMSO, Nutlin-3, and CL144. Red indicates downregulated proteins. (C) Venn diagram illustrating the overlap of downregulated proteins between two comparison groups: CL144 vs. DMSO and Nutlin-3 vs. DMSO.


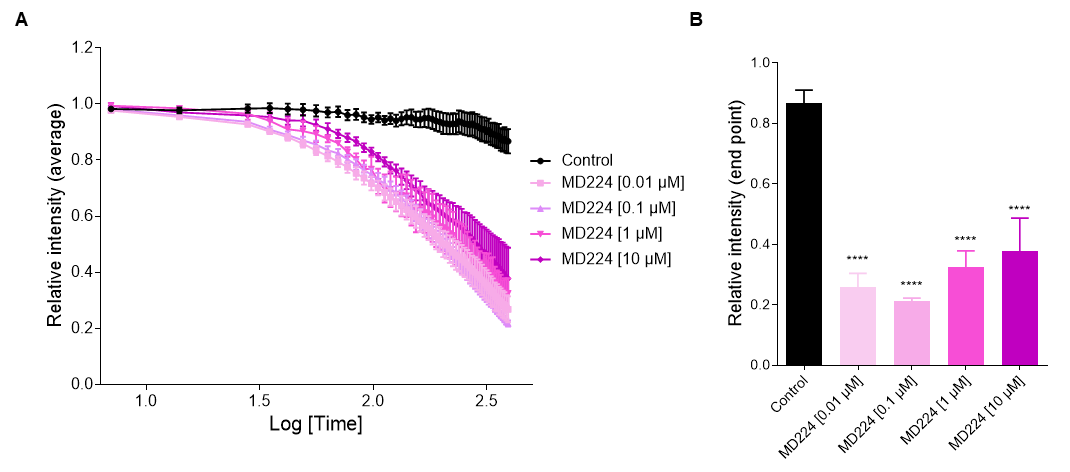


Figure S6. Validation of EGFP-MDM2 degradation kinetics and maximum efficiency of MD224 at various concentration

(A) Live imaging quantification of cells expressing EGFP-MDM2 after treatment with previously characterized MDM2-PROTACs (MD224). Quantification values were calculated from the EGFP signals; mean with SEM; n=3 to 8. (B) Evaluation of the maximum degradation efficiency of MD224 at 0.01 to 10 μM concentrations; ANOVA Bonferroni test: *****p*<0.0001; mean with SEM; n=3 to 8


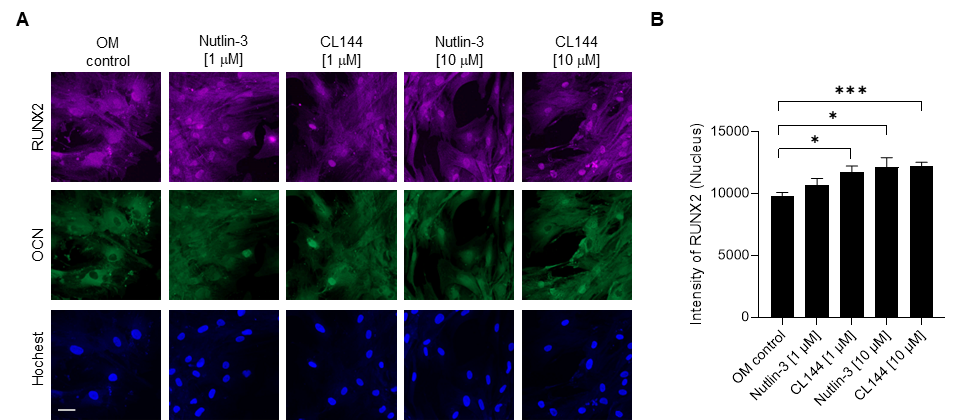


Figure S7. Immunostaining of key factors inducing osteogenic differentiation: RUNX2 and Osteocalcin (OCN) (A) Immunofluorescence images of RUNX2 and Osteocalcin in MDM2 inhibitor (Nutlin-3) and MDM2-PROTAC (CL144) treated human bone marrow-derived stem cells (hBMSCs). Scale bar = 50 μm; mean with SEM; n=5 to 9. (B) Quantitative analysis of RUNX2 from the images in (A); Student’s t-test **p*<0.05, ****p*<0.005; mean with SEM; n=5 to 9


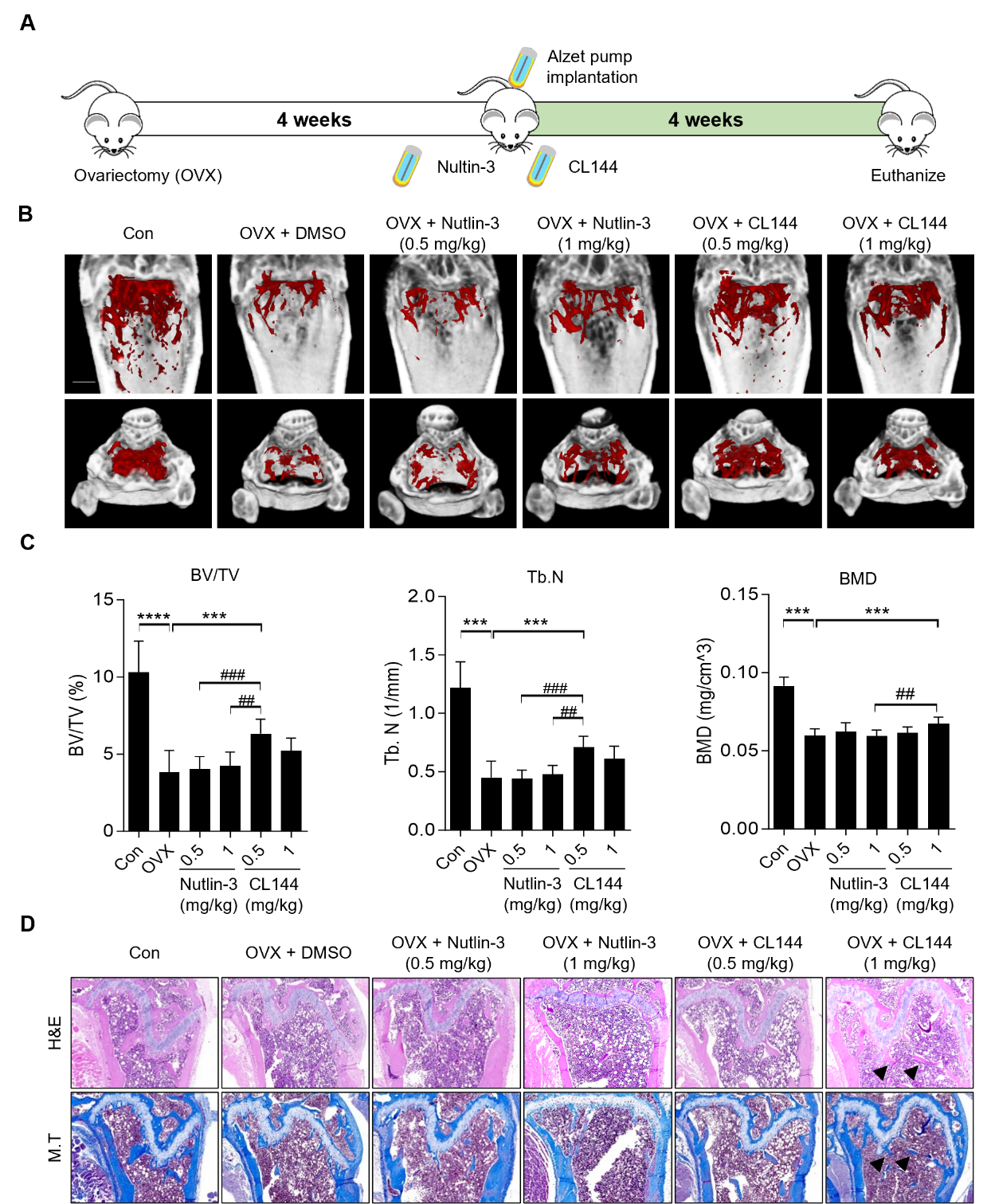


Figure S8. Comparative evaluation of MDM2-PROTAC and Nutlin-3 in bone regeneration.

(A) Schematic diagram of OVX-induced osteoporosis model; 4 weeks administration of CL144 or Nutlin-3 using implanted Alzet pumps in mice induced with osteoporotic conditions through ovariectomy (OVX) surgery. (B) The micro-CT 3D analysis of femur tissues in an OVX-induced osteoporosis model, following administration of Nultin-3 or CL144 at various concentrations; white structure: cortical bone, red structure: trabecular bone, Scale bar = 200 μm. (C) The bone formation parameters analysis of bone volume/tissue volume (BV/TV), trabecular number (Tb.N), and bone mineral density (BMD) in OVX model; ANOVA Bonferroni test: ***p*<0.005, ****p*<0.001, *****p*<0.0001, ##*p*<0.01, ###*p*<0.001; mean with SEM; n=8 to 10. (D) Histological analysis of the model femur tissue using H&E and Masson's trichrome staining. Black arrow heads indicate newly formed bone.


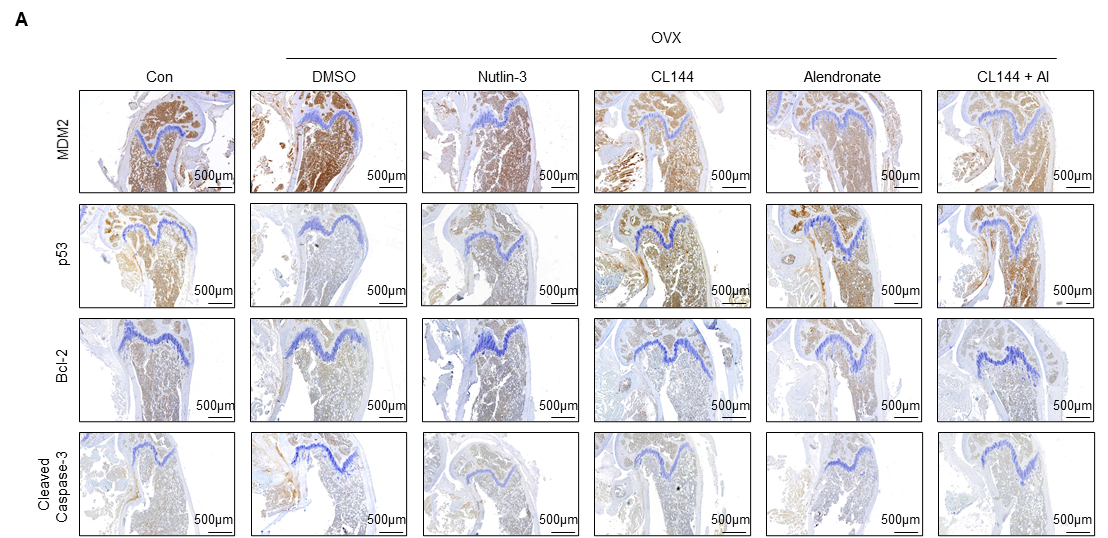


Figure S9. Assessment of bone marrow apoptosis in osteoporotic mice model treated with MDM2-PROTAC.

Immunohistochemistry analysis of the model femur tissue in preclinical models. Bcl-2 is used anti-apoptotic marker and Cleaved-caspase3 is used pro-apoptotic marker; Scale bar = 500 μm

Table S1. The list of MDM2 inhibitors used in this study

| No. | Company | Molecule name | Cat. No. | Solvent | Final conc. (μM) |
| --- | --- | --- | --- | --- | --- |
| 1 | Sigma | Nutlin-3 | N6287 | DMSO | 10 |
| 2 | MCE | Nutlin-3a | HY-10029 | DMSO | 10 |
| 3 | MCE | RG7112 (RO5045337) | HY-15676 | DMSO | 10 |
| 4 | MCE | Idasanutlin (RG7388) | HY-15676 | DMSO | 10 |
| 5 | MCE | Navtemadlin (AMG-232) | HY-12296 | DMSO | 10 |
| 6 | MCE | Alrizomadlin (APG-115) | HY-101518 | DMSO | 10 |
| 7 | MCE | NVP-CGM097 (CGM097) | HY-15954 | DMSO | 10 |
| 8 | MCE | Siremadlin (NVP-HDM201) | HY-18658 | DMSO | 10 |
| 9 | MCE | Milademetan (DS-3032) | HY-101266 | DMSO | 10 |
| 10 | MCE | MI-773 | HY-17493 | DMSO | 10 |
| 11 | Adooq | YH239-EE | A14118 | DMSO | 10 |
| 12 | MCE | NSC 66811 | HY-14967 | DMSO | 10 |
| 13 | MCE | Lithocholic acid | HY-B0172 | DMSO | 10 |
| 14 | Sigma | α-Mangostin | M3824 | MeOH | 10 |
| 15 | Sigma | *R,S*-Gambogic acid | PHL80455 | DMSO | 10 |
| 16 | MCE | SJ-172550 | HY-16664 | DMSO | 10 |
| 17 | MCE | Serdemetan (JNJ-26854165) | HY-12025 | DMSO | 10 |
| 18 | Tocris | RITA | 2443 | DMSO | 10 |
| 19 | MCE | SP-141 | HY-110182 | DMSO | 10 |
| 20 | MedKoo | Cytarabine hydrochloride (MK-8242) | 100200 | DMSO | 10 |
| 21 | Calbiochem | RO-5963 | 444153 | DMSO | 10 |
| 22 | MCE | MI-1061 | HY-125858 | DMSO | 10 |

Table S2. The list of antibodies and primers used in this study

| Antibodies | Source | Identifier |
| --- | --- | --- |
| anti -MDM2 | Santa Cruz | sc-965 |
| anti -p53 | Santa Cruz | sc-126 |
| anti -GAPDH | Santa Cruz | sc-47724 |
| anti-HA-tag | CST | #3724 |
| Mouse anti-Rabbit IgG-HRP | Santa Cruz | sc-2357 |
| Goat anti-mouse IgG(H+L)-HRP | GenDEPOT | SA001-500 |
| qRT-PCR Primer sequences | Gene name | forward/reverse |
| ACGGATTTGGCCGTATT | GAPDH | forward |
| TTGACTGTGCCGTGGAATTTG | GAPDH | reverse |
| AACCCTTAATTTGCACTGGGTCA | RUNX2 | forward |
| CAAATTCCAGCAATGTTTGTGCTAC | RUNX2 | reverse |
| CCCAGGCGCTACCTGTATCAA | OCN | forward |
| GGTCAGCCAACTCGTCACAGTC | OCN | reverse |
| ACACATATGATGGCCGAGGTGA | OPN | forward |
| TGTGAGGTGATGTCCTCGTCTGTAG | OPN | reverse |
| Cloning insert PCR sequences | Gene name | forward/reverse |
| GTAGAATTCGGCCACCATGTGCAATACCAACA | EcoR1-MDM2 | forward |
| GTAGGATCCCCGGGGAAATAAGTTAGCACAATCA | BamH1-MDM2 | reverse |

Table S3. The list of MDM2 inhibitors used in this study and binding affinity

| No. | Molecule name | | Dissociation constant (*K_d_*) value (nM) | Reference  (*K_d_*) | | Reported drugs Conc. (μM) | Reference (drug conc.) |
| --- | --- | --- | --- | --- | --- | --- | --- |
| 1 | Nutlin-3 | 240 | | | BindingDB (BDBM31197) | 1-10 | ^[35]^ |
| 2 | Nutlin-3a | - | | |  | 10 | ^[36]^ |
| 3 | RG7112 (RO5045337) | 11 | | | ^[37]^ | 5 | ^[35]^ |
| 4 | Idasanutlin (RG7388) | 9.8 | | | ^[38]^ | ~1.8 | ^[39]^ |
| 5 | Navtemadlin (AMG-232) | 0.045 | | | ^[40]^ | 10 | ^[41]^ |
| 6 | Alrizomadlin (APG-115) | - | | |  | ~1 | ^[42]^ |
| 7 | NVP-CGM097 (CGM097) | 2.3 | | | ^[43]^ | ~1 | ^[43]^ |
| 8 | Siremadlin (NVP-HDM201) | - | | |  | 10 | ^[44]^ |
| 9 | Milademetan (DS-3032) | - | | |  | ~2 | ^[45]^ |
| 10 | MI-773 | 8.2 | | | ^[46]^ | ~20 | ^[47]^ |
| 11 | YH239-EE | 300 | | | ^[48]^ | 20 | ^[48]^ |
| 12 | NSC 66811 | - | | |  | 20 | ^[49]^ |
| 13 | Lithocholic acid | 660 | | | ^[50]^ | 400 | ^[50]^ |
| 14 | α-Mangostin | - | | |  | 10 | ^[51]^ |
| 15 | *R,S*-Gambogic acid | - | | |  | 10 | ^[51]^ |
| 16 | SJ-172550 | - | | |  | ~15 | ^[52]^ |
| 17 | Serdemetan (JNJ-26854165) | - | | |  | ~10 | ^[53]^ |
| 18 | RITA | 1.5 | | | ^[54]^ | 10 | ^[54]^ |
| 19 | SP-141 | 43 | | | ^[55]^ | ~1 | ^[55]^ |
| 20 | Cytarabine hydrochloride  (MK-8242) | - | | |  | ~10 | ^[56]^ |
| 21 | RO-5963 | - | | |  | ~20 | ^[57]^ |
| 22 | MI-1061 | 1.4 | | | ^[58]^ | ~0.3 | ^[18]^ |
