# Supplementary material for: Development of MDM2‐Targeting PROTAC for Advancing Bone Regeneration
Source: Adv Sci (Weinh). 2025 Mar 24;12(19):2415626. doi: 10.1002/advs.202415626 (PMC12097015; doi:10.1002/advs.202415626)
Supplement: Supplementary file 2 — Supporting Information [file ADVS-12-2415626-s002.pdf]

ADVANCED SCIENCE
Open Access

Supporting Information

for *Adv. Sci.*, DOI 10.1002/advs.202415626

Development of MDM2-Targeting PROTAC for Advancing Bone Regeneration

Sol Jeong, Jae-Kook Cha, Wasim Ahmed, Jaewan Kim, Minsup Kim, Kyung Tae Hong, Wonji Choi, Sunjoo Choi, Tae Hyeon Yoo, Hyun-Ju An, Seung Chan An, Jaemin Lee, Jimin Choi, Sun-Young Kim, Jun-Seok Lee, Soonchul Lee, Junwon Choi* and Jin Man Kim**

Development of MDM2-targeting PROTAC for advancing bone regeneration

Sol Jeong[1†], Jae-Kook Cha[2,3†], Wasim Ahmed[4†], Jaewan Kim[4], Minsup Kim[5], Kyung Tae Hong[6], Wonji Choi[4], Sunjoo Choi[4], Tae Hyeon Yoo[4], Hyun-Ju An[7], Seung Chan An[7], Jaemin Lee[7], Jimin Choi[2], Sun-Young Kim[8], Jun-Seok Lee[6], Soonchul Lee[7]*, Junwon Choi[4,9]*, Jin Man Kim[1,10,11]*

[1]*Department of Oral Microbiology and Immunology, School of Dentistry and Dental Research Institute, Seoul National University, Seoul, 08826, Republic of Korea*

[2]*Department of Periodontology, Research Institute of Periodontal Regeneration, College of Dentistry, Yonsei University, Seoul, 03722, Republic of Korea*

[3]*Department of Oral Medicine, Infection, and Immunity, Harvard School of Dental Medicine, Boston, 02115, USA*

[4]*Department of Molecular Science and Technology, Ajou University, Gyeonggi-do, 16499, Republic of Korea*

[5]*TARS Scientific, Seoul, 01717, Republic of Korea*

[6]*Department of Pharmacology, Korea University College of Medicine, Korea University, Seoul, 02841, Republic of Korea*

[7]*Department of Orthopaedic Surgery, CHA Bundang Medical Center, CHA University School of Medicine, Gyeonggi-do, 13488, Republic of Korea*

[8]*Department of Conservative Dentistry and Dental Research Institute, School of Dentistry, Seoul National University, Seoul, 08826, Republic of Korea*

[9]*Advanced College of Bio-convergence Engineering, Ajou University, Gyeonggi-do, 16499, Republic of Korea*

[10]*Dental Multiomics Center, School of Dentistry and Dental Research Institute, Seoul National University, Gwanak-ro 1, Gwanak-gu, Seoul 08826, Republic of Korea*

[11]*Innovative Pharmaceutical Sciences Program, College of Transdisciplinary Innovations, Seoul National University, Gwanak-ro 1, Gwanak-gu, Seoul 08826, Republic of Korea*

*Corresponding author. Email: jinmankim@snu.ac.kr (J.M.K.); junwchoi@ajou.ac.kr (J.C.); lsceline@cha.ac.kr (S.L.)

Supporting information

Table of contents

1. General Information S-2

2. Preparation of MDM2-PROTAC S-2

3. NMR spectra (^{1}H NMR, ^{13}C NMR) S-23

1. General Information

Unless otherwise noted, reagents were obtained from commercial suppliers and used without further purification. Anhydrous THF, DMF, CH_2Cl_2, MeCN, and DMSO were purchased from Sigma–Aldrich. Pyridinium chlorochromate (PCC), 1-[bis(dimethylamino)methylene]-1*H*-1,2,3-triazolo[4,5-b]pyridinium 3-oxide hexafluorophosphate (HATU), dimethyl (1-diazo-2-oxopropyl)phosphonate, copper(I) iodide (CuI), bis(triphenylphosphine)palladium(II) dichloride ($PdCl_2(PPh_3)_2$), di-*tert*-butyl dicarbonate ($(Boc)_2O$), *N*,*N*-diisopropylethylamine (DIPEA), and trifluoroacetic acid (TFA) were purchased from Sigma–Aldrich, Acros Organics, Alfa Aesar, Angene, Combi-blocks, or TCI. Unless stated otherwise, reactions were conducted under an atmosphere of N_2 using anhydrous solvents. Analytical thin-layer chromatography (TLC) was performed with TLC Silica gel 60 F254 (Merck) and visualized by UV fluorescence quenching, ninhydrin, or $KMnO_4$ stain. Flash column chromatography was performed on a Biotage® Selekt Flash Purification System using Biotage® Sfär Silica or Biotage® Sfär C18 D-Duo 100Å cartridges. Preparative HPLC was performed on an Agilent 1260 system with an Agilent 10 prep-C18 column (100Å, length 250 mm, I.D. 21.2 mm, 10 µm) with a solvent flow rate of 20 mL/min. All NMR spectra were recorded on JNM-ECZR 600 MHz or Bruker 400 MHz spectrometers. ^{1}H NMR spectra were reported relative to residual $CDCl_3$ (δ 7.26 ppm), CD_3OD (3.31 ppm), $(CD_3)_2SO$ (δ 2.50 ppm), or CD_3CN (δ 1.94 ppm). ^{13}C NMR spectra were reported relative to residual CD_3OD (δ 49.00 ppm). Data for ^{1}H and ^{13}C NMR spectra were reported in terms of chemical shifts (δ ppm). HRMS was taken on a Bruker Ultra High-Resolution ESI Q-TOF mass spectrometer.

2. Preparation of MDM2-PROTAC

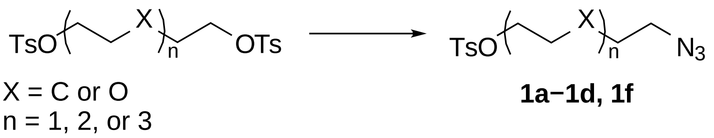

1a–1d, 1f

General Procedure 1 (GP-1). Ditosylated compounds were prepared from corresponding diols according to a literature procedure.[1] The ditosylated compound (3.00 equiv) was added to a solution of NaN_3 (1.00 equiv) in anhydrous MeCN in a round-bottom flask. The reaction mixture was stirred at 70 °C for 16 h. After completion of the reaction, the mixture was cooled to room temperature. The mixture was diluted with ethyl acetate and filtered. The filtrate was concentrated under reduced pressure, and the resulting residue was purified by flash column chromatography on silica gel.

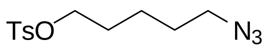

5-Azidopentyl 4-methylbenzenesulfonate (1a). The title compound was synthesized from NaN_3 (300 mg, 4.61 mmol) and pentane-1,5-diyl bis(4-methylbenzenesulfonate) (5.70 g, 13.8 mmol) in anhydrous MeCN (30.0 mL) according to **GP-1**. The product was purified by flash column chromatography on silica gel (0%→90% ethyl acetate/hexanes): 960 mg (73%). Colorless oil.
^{1}H NMR (600 MHz, $CDCl_3$) δ 7.81–7.77 (m, 2H), 7.37–7.33 (m, 2H), 4.03 (t, *J* = 6.3 Hz, 2H), 3.23 (t, *J* = 6.8 Hz, 2H), 2.45 (s, 3H), 1.71–1.65 (m, 2H), 1.57–1.51 (m, 2H), 1.43–1.37 (m, 2H).

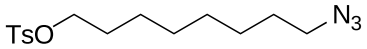

(1) Kim, D.-Y.; Kim, H.-J.; Yu, K.-H.; Min J.-J. *Bioconjugate Chem.* **2012**, *23*, 431−437.

8-Azidooctyl 4-methylbenzenesulfonate (1b). The title compound was synthesized from NaN_3 (170 mg, 2.61 mmol) and octane-1,8-diyl bis(4-methylbenzenesulfonate) (3.57 g, 7.85 mmol) in anhydrous MeCN (30.0 mL) according to **GP-1**. The product was purified by flash column chromatography on silica gel (8% ethyl acetate/petroleum ether): 720 mg (85%). Colorless oil.
^{1}H NMR (600 MHz, $CDCl_3$) δ 7.81–7.77 (m, 2H), 7.37–7.32 (m, 2H), 4.02 (t, *J* = 6.5 Hz, 2H), 3.24 (t, *J* = 6.9 Hz, 2H), 2.45 (s, 3H), 1.63 (dq, *J* = 7.9, 6.6 Hz, 2H), 1.60–1.53 (m, 3H), 1.35–1.20 (m, 7H).

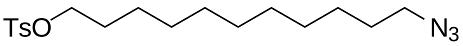

11-Azidoundecyl 4-methylbenzenesulfonate (1c). The title compound was synthesized from NaN_3 (250 mg, 3.85 mmol) and undecane-1,11-diyl bis(4-methylbenzenesulfonate) (5.70 g, 11.5 mmol) in anhydrous MeCN (20.0 mL) according to **GP-1**. The product was purified by flash column chromatography on silica gel (6% ethyl acetate/petroleum ether): 530 mg (38%). Colorless oil.
^{1}H NMR (600 MHz, $CDCl_3$) δ 7.80–7.77 (m, 2H), 7.36–7.33 (m, 2H), 4.01 (t, *J* = 6.5 Hz, 2H), 3.25 (t, *J* = 7.0 Hz, 2H), 2.45 (s, 3H), 1.66–1.56 (m, 4H), 1.38–1.19 (m, 14H).

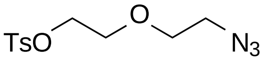

2-(2-Azidoethoxy)ethyl 4-methylbenzenesulfonate (1d). The title compound was synthesized from NaN_3 (100 mg, 1.54 mmol) and oxybis(ethane-2,1-diyl) bis(4-methylbenzenesulfonate) (1.91 g, 4.61 mmol) in anhydrous MeCN (8.00 mL) according to **GP-1**. The product was purified by flash column chromatography on silica gel (40% ethyl acetate/petroleum ether): 330 mg (75%). Colorless oil.
^{1}H NMR (600 MHz, $CDCl_3$) δ 7.83–7.78 (m, 2H), 7.37–7.33 (m, 2H), 4.19–4.15 (m, 2H), 3.72–3.68 (m, 2H), 3.62–3.59 (m, 2H), 3.32 (d, *J* = 5.1 Hz, 2H), 2.45 (s, 3H).

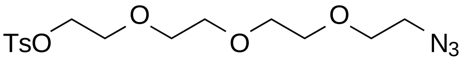

2-(2-(2-(2-Azidoethoxy)ethoxy)ethoxy)ethyl 4-methylbenzenesulfonate (1f). The title compound was synthesized from NaN_3 (250 mg, 3.85 mmol) and ((oxybis(ethane-2,1-diyl))bis(oxy))bis(ethane-2,1-diyl) bis(4-methylbenzenesulfonate) (5.80 g, 11.5 mmol) in anhydrous MeCN (20.0 mL) according to **GP-1**. The product was purified by flash column chromatography on silica gel (45% ethyl acetate/petroleum ether): 830 mg (58%). Colorless oil.
^{1}H NMR (600 MHz, $CDCl_3$) δ 7.81–7.78 (m, 2H), 7.36–7.32 (m, 2H), 4.18–4.14 (m, 2H), 3.70–3.57 (m, 10H), 3.38 (t, *J* = 5.1 Hz, 2H), 2.45 (s, 3H), 1.63–1.57 (m, 2H).

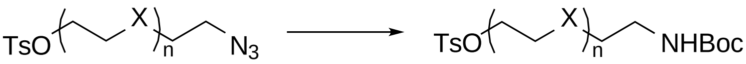

1a–1d, 1f **2a–2d, 2f**

X = C or O
n = 1, 2, or 3

General Procedure 2 (GP-2). Di-*tert*-butyl dicarbonate (1.20 equiv) was added to a stirred solution of the azide (1.00 equiv) in anhydrous THF in a round-bottom flask. The flask was purged with a stream of N_2. Pd/C (10 wt%; 0.10 equiv) was added to the reaction mixture, and the reaction mixture was stirred under atmospheric pressure of H_2 at room temperature for 1 h. After completion of the reaction, the mixture was filtered through a pad of Celite®, and the Celite® was washed with ethyl acetate. The resulting filtrate was concentrated under reduced pressure and purified by flash column chromatography on silica gel.

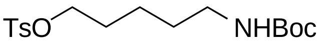

5-((*tert*-Butoxycarbonyl)amino)pentyl 4-methylbenzenesulfonate (2a). The title compound was synthesized from (5-azidopentyl 4-methylbenzenesulfonate (**1a**, 500 mg, 1.76 mmol) in anhydrous THF (10.0 mL) according to **GP-2**. The product was purified by flash column chromatography on silica gel (10% ethyl acetate/hexanes→20% ethyl acetate/hexanes): 430 mg (68 %). Colorless oil.
^{1}H NMR (600 MHz, $CDCl_3$) δ 7.80–7.77 (m, 2H), 7.34 (d, *J* = 8.0 Hz, 2H), 4.49 (br s, 1H), 4.01 (t, *J* = 6.4 Hz, 2H), 3.10–3.01 (m, 2H), 2.45 (s, 3H), 1.68–1.62 (m, 2H), 1.46–1.39 (m, 11H), 1.36–1.30 (m, 2H).

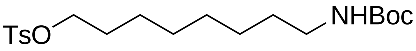

8-((*tert*-Butoxycarbonyl)amino)octyl 4-methylbenzenesulfonate (2b). The title compound was synthesized from 8-azidooctyl 4-methylbenzenesulfonate (**1b**, 250 mg, 0.768 mmol) in anhydrous THF (10.0 mL) according to **GP-2**. The product was purified by flash column chromatography on silica gel (20% ethyl acetate/petroleum ether): 230 mg (75%). Colorless oil.
^{1}H NMR (600 MHz, $CDCl_3$) δ 7.80–7.77 (m, 2H), 7.34 (d, *J* = 8.0 Hz, 2H), 4.49 (br s, 1H), 4.01 (t, *J* = 6.5 Hz, 2H), 3.08 (q, *J* = 6.7 Hz, 2H), 2.45 (s, 3H), 1.66–1.56 (m, 4H), 1.44 (s, 9H), 1.33–1.18 (m, 8H).

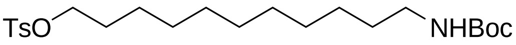

11-((*tert*-Butoxycarbonyl)amino)undecyl 4-methylbenzenesulfonate (2c). The title compound was synthesized from 11-azidoundecyl 4-methylbenzenesulfonate (**1c**, 300 mg, 0.816 mmol) in anhydrous THF (10.0 mL) according to **GP-2**. The product was purified by flash column chromatography on silica gel (20% ethyl acetate/hexanes): 290 mg (80%). White solid.
^{1}H NMR (600 MHz, $CDCl_3$) δ 7.80–7.77 (m, 2H), 7.34 (d, *J* = 8.0 Hz, 2H), 4.49 (br s, 1H), 4.01 (t, *J* = 6.5 Hz, 2H), 3.12–3.04 (m, 2H), 2.45 (s, 3H), 1.67–1.59 (m, 3H), 1.44 (s, 9H), 1.32–1.17 (m, 15H).

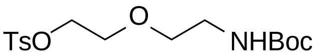

2-(2-((*tert*-Butoxycarbonyl)amino)ethoxy)ethyl 4-methylbenzenesulfonate (2d). The title compound was synthesized from 2-(2-azidoethoxy)ethyl 4-methylbenzenesulfonate (**1d**, 160 mg, 0.561 mmol) in anhydrous THF (5.00 mL) according to **GP-2**. The product was purified by flash column chromatography on silica gel (40% ethyl acetate/petroleum ether): 150 mg (74%). Colorless oil.
^{1}H NMR (600 MHz, $CDCl_3$) δ 7.82–7.79 (m, 2H), 7.35 (d, *J* = 8.0 Hz, 2H), 4.80 (br s, 1H), 4.19–4.14 (m, 2H), 3.64–3.61 (m, 2H), 3.45 (t, *J* = 5.2 Hz, 2H), 3.26–3.20 (m, 2H), 2.45 (s, 3H), 1.45 (s, 9H).

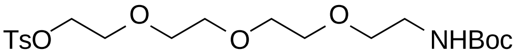

2,2-Dimethyl-4-oxo-3,8,11,14-tetraoxa-5-azahexadecan-16-yl 4-methylbenzenesulfonate (2f). The title compound was synthesized from 2-(2-(2-(2-azidoethoxy)ethoxy)ethoxy)ethyl 4-methylbenzenesulfonate (**1f**, 200 mg, 0.536 mmol) in anhydrous THF (8.00 mL) according to **GP-2**. The product was purified by column chromatography on silica gel (60% ethyl acetate/petroleum ether): 190 mg (79%). Colorless oil.

^{1}H NMR (600 MHz, $CDCl_3$) δ 7.81–7.78 (m, 2H), 7.34 (d, *J* = 7.9 Hz, 2H), 5.02 (br s, 1H), 4.18–4.15 (m, 2H), 3.71–3.68 (m, 2H), 3.62–3.57 (m, 8H), 3.52 (t, *J* = 5.2 Hz, 2H), 3.32–3.26 (m, 2H), 2.44 (s, 3H), 1.43 (s, 9H).

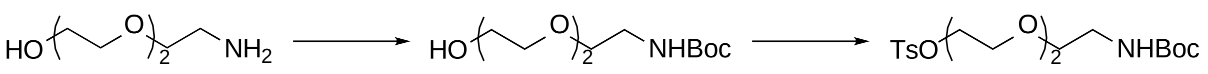

2e

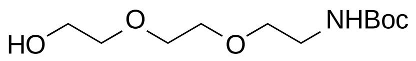

***tert*-Butyl (2-(2-(2-hydroxyethoxy)ethoxy)ethyl)carbamate.** To a stirred solution of 2-(2-(2-aminoethoxy)ethoxy)ethan-1-ol (900 mg, 6.03 mmol) in 1,4-dioxane (14.0 mL) in a 50-mL round-bottom flask was added di-*tert*-butyl dicarbonate (1.32 g, 6.05 mmol). The reaction mixture was stirred at room temperature for 3 h. After completion of the reaction, the mixture was diluted with water (50 mL) and ethyl acetate (50 mL), stirred vigorously, and extracted with ethyl acetate (2 × 50 mL). The combined organic layer was washed with water, dried over Na_2SO_4, filtered, and concentrated under reduced pressure. The desired product was purified by flash column chromatography on silica gel (1:1 ethyl acetate/hexanes): 1.04 g (69%). Light-yellow oil.
^{1}H NMR (400 MHz, $CDCl_3$) δ 5.10 (br s, 1H), 3.79–3.72 (m, 2H), 3.69–3.59 (m, 6H), 3.56 (t, *J* = 5.2 Hz, 2H), 3.32 (t, *J* = 5.2 Hz, 2H), 2.11 (br s, 1H), 1.45 (s, 9H).

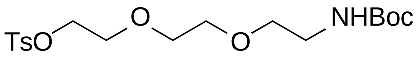

2,2-Dimethyl-4-oxo-3,8,11-trioxa-5-azatridecan-13-yl 4-methylbenzenesulfonate (2e). *tert*-Butyl (2-(2-(2-hydroxyethoxy)ethoxy)ethyl)carbamate (900 mg, 3.61 mmol) was dissolved in CH_2Cl_2 (12.0 mL) in a 50-mL round-bottom flask and cooled to 0 °C. Tosyl chloride (1.03 g, 5.40 mmol), Et_3N (1.51 mL, 10.8 mmol), and 4-(dimethylamino)pyridine (44.1 mg, 0.361 mmol) were added to the solution at 0 °C. The reaction mixture was stirred at 0 °C for 4.5 h. After completion of the reaction, the mixture was diluted with $CHCl_3$ (100 mL), washed with brine (100 mL), dried over Na_2SO_4, filtered, and concentrated under reduced pressure. The desired product was purified by flash column chromatography on silica gel (0%→80% ethyl acetate/hexanes): 969 mg (67%). Light-yellow oil.
^{1}H NMR (400 MHz, $CDCl_3$) δ 7.80 (d, *J* = 8.0 Hz, 2H), 7.34 (d, *J* = 8.0 Hz, 2H), 4.94 (br s, 1H), 4.17 (t, *J* = 4.8 Hz, 2H), 3.69 (t, *J* = 5.1 Hz, 2H), 3.60–3.52 (m, 4H), 3.50 (t, *J* = 5.1 Hz, 2H), 3.29 (m, 2H), 2.45 (s, 3H), 1.43 (s, 9H).

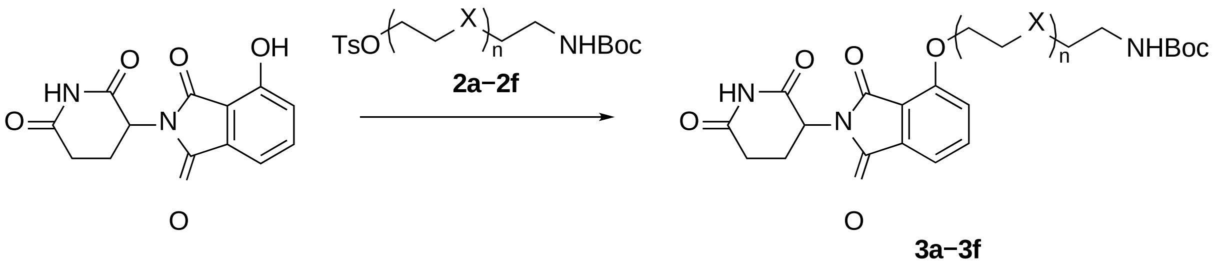

3a−3f

General Procedure 3 (GP-3). A sealed tube was charged with the alkyl tosylate (**2**) and anhydrous MeCN. 2-(2,6-Dioxopiperidin-3-yl)-4-hydroxyisoindoline-1,3-dione and DIPEA were added to the solution, and the reaction mixture was stirred at 70 °C for 16 h. After completion of the reaction, the mixture was cooled to room temperature and filtered through a pad of Celite®, and the Celite® was

washed with ethyl acetate. The resulting filtrate was concentrated under reduced pressure and purified by flash column chromatography on silica gel.

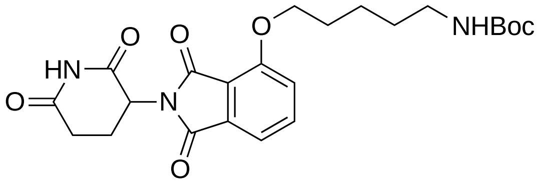

***tert*-Butyl (5-((2-(2,6-dioxopiperidin-3-yl)-1,3-dioxoisoindolin-4-yl)oxy)pentyl)carbamate (3a).** The title compound was synthesized from 2-(2,6-dioxopiperidin-3-yl)-4-hydroxyisoindoline-1,3-dione (250 mg, 0.912 mmol), 5-((*ter*t-butoxycarbonyl)amino)pentyl 4-methylbenzenesulfonate (**2a**, 340 mg, 0.951 mmol), and DIPEA (1.40 mL, 8.04 mmol) in anhydrous MeCN (10.0 mL) according to **GP-3**. The product was purified by column chromatography on silica gel (20% acetone/hexanes): 160 mg (38%). White solid.

^{1}H NMR (600 MHz, $CDCl_3$) δ 7.96 (s, 1H), 7.67 (dd, *J* = 8.4, 7.3 Hz, 1H), 7.46 (d, *J* = 7.1 Hz, 1H), 7.21 (d, *J* = 8.4 Hz, 1H), 4.95 (dd, *J* = 12.6, 5.4 Hz, 1H), 4.62 (s, 1H), 4.22–4.14 (m, 2H), 3.18–3.11 (m, 2H), 2.94–2.69 (m, 3H), 2.16–2.11 (m, 1H), 1.93–1.88 (m, 2H), 1.61–1.52 (m, 4H), 1.44 (s, 9H).

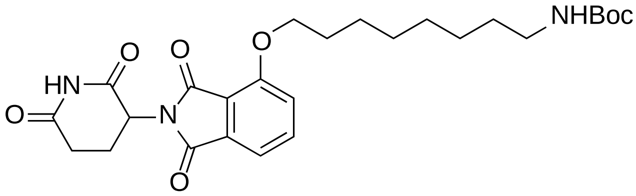

***tert*-Butyl (8-((2-(2,6-dioxopiperidin-3-yl)-1,3-dioxoisoindolin-4-yl)oxy)octyl)carbamate (3b).** The title compound was synthesized from 2-(2,6-dioxopiperidin-3-yl)-4-hydroxyisoindoline-1,3-dione (130 mg, 0.474 mmol), 8-((*tert*-butoxycarbonyl)amino)octyl 4-methylbenzenesulfonate (**2b**, 200 mg, 0.501 mmol), and DIPEA (0.730 mL, 4.19 mmol) in anhydrous MeCN (5.00 mL) according to **GP-3**. The product was purified by column chromatography on silica gel (25% acetone/hexanes): 100 mg (42%). White solid.

^{1}H NMR (600 MHz, $CDCl_3$) δ 8.12 (s, 1H), 7.67 (dd, *J* = 8.5, 7.3 Hz, 1H), 7.45 (d, *J* = 7.1 Hz, 1H), 7.21 (d, *J* = 8.5 Hz, 1H), 4.99–4.92 (m, 1H), 4.54 (s, 1H), 4.21–4.13 (m, 2H), 3.14–3.08 (m, 2H), 2.92–2.69 (m, 3H), 2.16–2.09 (m, 1H), 1.87 (p, *J* = 7.4, 6.9 Hz, 2H), 1.53–1.42 (m, 13H), 1.40–1.29 (m, 6H).

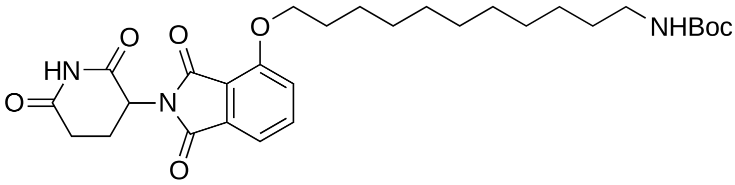

***tert*-Butyl (11-((2-(2,6-dioxopiperidin-3-yl)-1,3-dioxoisoindolin-4-yl)oxy)undecyl)carbamate (3c).** The title compound was synthesized from 2-(2,6-dioxopiperidin-3-yl)-4-hydroxyisoindoline-1,3-dione (120 mg, 0.438 mmol), 11-((*tert*-butoxycarbonyl)amino)undecyl 4-methylbenzenesulfonate (**2c**, 186 mg, 0.421 mmol), and DIPEA (0.670 mL, 3.85 mmol) in anhydrous MeCN (5.00 mL) according to **GP-3**. The product was purified by column chromatography on silica gel (25% acetone/hexanes): 80 mg (35%). White solid.

^{1}H NMR (600 MHz, $CDCl_3$) δ 7.98 (s, 1H), 7.66 (dd, *J* = 8.5, 7.3 Hz, 1H), 7.44 (d, *J* = 7.3 Hz, 1H), 7.20 (d, *J* = 8.5 Hz, 1H), 4.94 (dd, *J* = 12.5, 5.4 Hz, 1H), 4.49 (br s, 1H), 4.16 (t, *J* = 6.6 Hz, 2H), 3.12–3.05 (m, 2H), 2.93–2.69 (m, 3H), 2.14–2.09 (m, 1H), 1.87 (p, *J* = 14.0, 6.7 Hz, 2H), 1.51–1.41 (m, 13H), 1.37–1.23 (m, 12H).

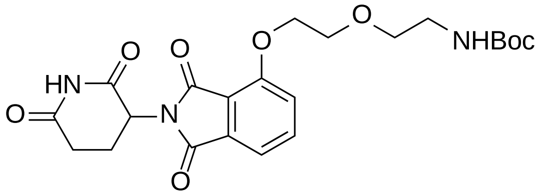

***tert*-Butyl (2-(2-((2-(2,6-dioxopiperidin-3-yl)-1,3-dioxoisoindolin-4-yl)oxy)ethoxy)ethyl)carbamate (3d).** The title compound was synthesized from 2-(2,6-dioxopiperidin-3-yl)-4-hydroxyisoindoline-1,3-dione (100 mg, 0.365 mmol), 2-(2-((*tert*-butoxycarbonyl)amino)ethoxy)ethyl 4-methylbenzenesulfonate (**2d**, 138 mg, 0.384 mmol) and DIPEA (0.600 mL, 3.44 mmol) in anhydrous MeCN (3.00 mL) according to **GP-3**. The product was purified by column chromatography on silica gel (40% acetone/hexanes): 70 mg (42%). White solid.
^{1}H NMR (600 MHz, $CDCl_3$) δ 7.98 (s, 1H), 7.69 (dd, *J* = 8.4, 7.3 Hz, 1H), 7.49 (dd, *J* = 7.3, 0.7 Hz, 1H), 7.27–7.25 (m, 1H), 5.04 (br s, 1H), 4.96 (dd, *J* = 12.6, 5.4 Hz, 1H), 4.34 (t, *J* = 4.8 Hz, 2H), 3.92–3.89 (m, 2H), 3.66 (t, *J* = 5.2 Hz, 2H), 3.37–3.30 (m, 2H), 2.93–2.87 (m, 1H), 2.83 (qd, *J* = 12.7, 4.0 Hz, 1H), 2.73 (ddd, *J* = 16.9, 13.6, 5.0 Hz, 1H), 2.16–2.10 (m, 1H), 1.42 (s, 9H).

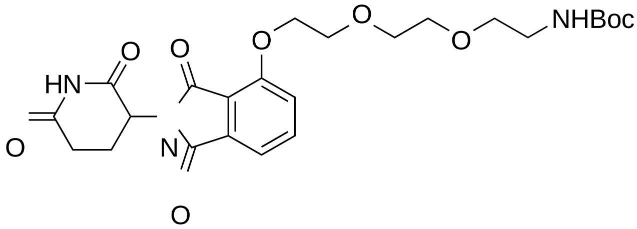

***tert*-Butyl (2-(2-(2-((2-(2,6-dioxopiperidin-3-yl)-1,3-dioxoisoindolin-4-yl)oxy)ethoxy)ethoxy)ethyl)carbamate (3e).** The title compound was synthesized from 2-(2,6-dioxopiperidin-3-yl)-4-hydroxyisoindoline-1,3-dione (150 mg, 0.547 mmol), 2,2-dimethyl-4-oxo-3,8,11-trioxa-5-azatridecan-13-yl 4-methylbenzenesulfonate (**2e**, 155 mg, 0.384 mmol), and DIPEA (0.560 mL, 3.21 mmol) in anhydrous MeCN (3.00 mL) according to **GP-3**. The product was purified by column chromatography on silica gel (40% acetone/hexanes): 155 mg (80%). White solid.
^{1}H NMR (600 MHz, $CDCl_3$) δ 8.20 (s, 1H), 7.68 (dd, *J* = 8.3, 7.4 Hz, 1H), 7.48 (d, *J* = 7.3 Hz, 1H), 7.27–7.25 (m, 1H), 5.03 (s, 1H), 4.95 (m, 1H), 4.37–4.35 (t, *J* = 4.7 Hz, 2H), 3.95 (t, *J* = 4.7 Hz, 2H), 3.78 (t, *J* = 4.5 Hz, 2H), 3.63 (t, *J* = 4.6 Hz, 2H), 3.57–3.52 (m, 2H), 3.33–3.27 (m, 2H), 2.93–2.69 (m, 3H), 2.15–2.10 (m, 1H), 1.43 (s, 9H).

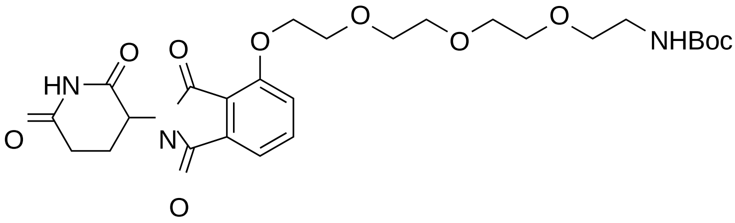

***tert*-Butyl (2-(2-(2-(2-((2-(2,6-dioxopiperidin-3-yl)-1,3-dioxoisoindolin-4-yl)oxy)ethoxy)ethoxy)ethoxy)ethyl)carbamate (3f).** The title compound was synthesized from 2-(2,6-dioxopiperidin-3-yl)-4-hydroxyisoindoline-1,3-dione (100 mg, 0.365 mmol), 2,2-dimethyl-4-oxo-3,8,11,14-tetraoxa-5-azahexadecan-16-yl 4-methylbenzenesulfonate (**2f**, 172 mg, 0.384 mmol), and

DIPEA (0.550 mL, 3.16 mmol) in anhydrous MeCN (5.00 mL) according to **GP-3**. The product was purified by column chromatography on silica gel (45% acetone/hexanes): 120 mg (60%). White solid. ^{1}H NMR (600 MHz, $CDCl_3$) δ 8.19 (s, 1H), 7.67 (dd, *J* = 8.5, 7.3 Hz, 1H), 7.47 (d, *J* = 7.1 Hz, 1H), 7.27 (d, *J* = 8.5 Hz, 1H), 5.06 (s, 1H), 4.95 (dd, *J* = 12.5, 5.4 Hz, 1H), 4.36 (t, *J* = 4.8 Hz, 2H), 3.96–3.94 (m, 2H), 3.81–3.78 (m, 2H), 3.68–3.65 (m, 2H), 3.65–3.58 (m, 4H), 3.53 (t, *J* = 5.2 Hz, 2H), 3.33–3.27 (m, 2H), 2.90–2.68 (m, 3H), 2.15–2.09 (m, 1H), 1.43 (s, 9H).

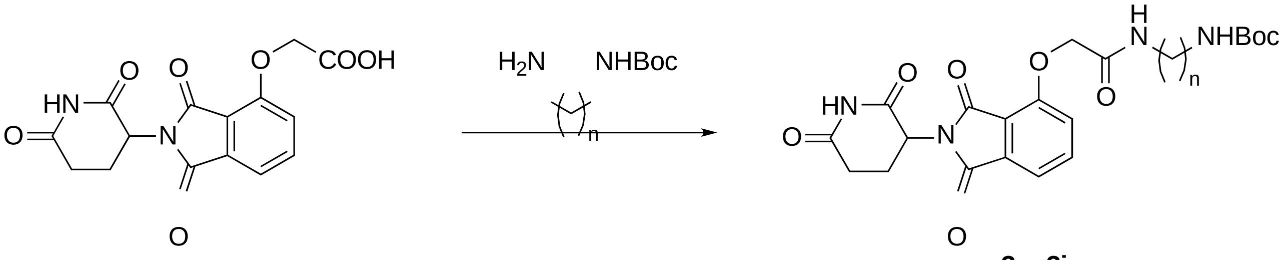

3g−3i

General procedure 4 (GP-4). 2-((2-(2,6-Dioxopiperidin-3-yl)-1,3-dioxoisoindolin-4-yl)oxy)acetic acid was prepared according to a literature procedure.[2] The amine (1.20 equiv), HATU (1.20 equiv), and DIPEA (3.00 equiv) were added to a solution of 2-((2-(2,6-dioxopiperidin-3-yl)-1,3-dioxoisoindolin-4-yl)oxy)acetic acid (1.00 equiv; 0.150 M) in anhydrous DMF in a round-bottom flask. The reaction mixture was stirred at room temperature for 16 h. After completion of the reaction, the mixture was diluted with aqueous HCl solution (1 M). The mixture was extracted with ethyl acetate. The combined organic layer was washed with a saturated aqueous solution of LiCl and brine, dried over Na_2SO_4, filtered, and concentrated under reduced pressure. The product was purified by flash column chromatography.

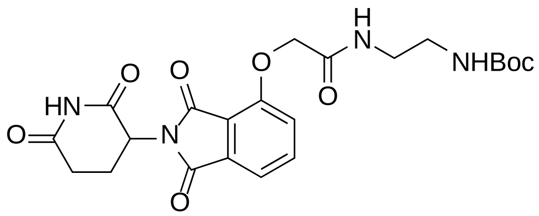

***tert*-Butyl (2-(2-((2-(2,6-dioxopiperidin-3-yl)-1,3-dioxoisoindolin-4-yl)oxy)acetamido)ethyl)carbamate (3g).** The title compound was synthesized from 2-((2-(2,6-dioxopiperidin-3-yl)-1,3-dioxoisoindolin-4-yl)oxy)acetic acid (100 mg, 0.301 mmol) and *tert*-butyl (2-aminoethyl)carbamate (58 mg, 0.36 mmol) according to **GP-4**. The product was purified by flash column chromatography on silica gel (1→10% MeOH/CH_2Cl_2): 68 mg (48%). White solid. ^{1}H NMR (600 MHz, DMSO-d_6) δ 11.12 (s, 1H), 8.05 (t, *J* = 5.8 Hz, 1H), 7.81 (dd, *J* = 8.5, 7.3 Hz, 1H), 7.50 (d, *J* = 7.2 Hz, 1H), 7.39 (d, *J* = 8.3 Hz, 1H), 6.87 (t, *J* = 5.8 Hz, 1H), 5.12 (dd, *J* = 12.9, 5.5 Hz, 1H), 4.76 (s, 2H), 3.16 (q, *J* = 6.5 Hz, 2H), 3.01 (q, *J* = 6.2 Hz, 2H), 2.89 (ddd, *J* = 17.3, 14.0, 5.6 Hz, 1H), 2.63–2.55 (m, 1H), 2.55–2.52 (m, 1H), 2.06–2.00 (m, 1H), 1.36 (s, 9H).

(2) Remillard, D.; Buckley, D. L.; Paulk, J.; Brien, G. L.; Sonnett, M.; Seo, H.-S.; Dastjerdi, S.; Wühr, M.; Dhe-Paganon, S.; Armstrong, S. A.; Bradner, J. E. *Angew. Chem., Int. Ed.* **2017**, *56*, 5738–5743.

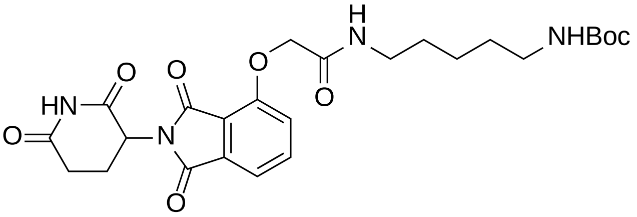

***tert*-Butyl (5-(2-((2-(2,6-dioxopiperidin-3-yl)-1,3-dioxoisoindolin-4-yl)oxy)acetamido)pentyl)carbamate (3h).** The title compound was synthesized from 2-((2-(2,6-dioxopiperidin-3-yl)-1,3-dioxoisoindolin-4-yl)oxy)acetic acid (100 mg, 0.301 mmol) and *tert*-butyl (5-aminopentyl)carbamate (73 mg, 0.36 mmol) according to **GP-4**. The product was purified by flash column chromatography on silica gel (1→10% MeOH/CH_2Cl_2): 90 mg (58%). White solid.
^{1}H NMR (600 MHz, DMSO-d_6) δ 11.12 (s, 1H), 7.94 (t, J = 5.9 Hz, 1H), 7.82 (dd, J = 8.5, 7.3 Hz, 1H), 7.49 (d, J = 7.2 Hz, 1H), 7.39 (d, J = 8.5 Hz, 1H), 6.77 (t, J = 5.6 Hz, 1H), 5.12 (dd, J = 12.9, 5.5 Hz, 1H), 4.76 (s, 2H), 3.12 (q, J = 6.7 Hz, 2H), 2.94–2.85 (m, 3H), 2.62–2.58 (m, 1H), 2.57–2.52 (m, 1H), 2.06–2.00 (m, 1H), 1.45–1.39 (m, 2H), 1.39–1.32 (s, 11H), 1.26–1.20 (m, 2H).

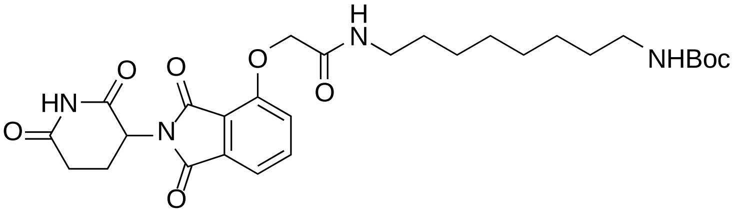

***tert*-Butyl (8-(2-((2-(2,6-dioxopiperidin-3-yl)-1,3-dioxoisoindolin-4-yl)oxy)acetamido)octyl)carbamate (3i).** The title compound was synthesized from 2-((2-(2,6-dioxopiperidin-3-yl)-1,3-dioxoisoindolin-4-yl)oxy)acetic acid (100 mg, 0.301 mmol) and *tert*-butyl (8-aminoethyl)carbamate (88 mg, 0.36 mmol) according to **GP-4**. The product was purified by flash column chromatography on silica gel (1→10% MeOH/CH_2Cl_2) and then on C-18 silica gel (10→60% MeCN/water): 87 mg (52%). White solid.
^{1}H NMR (600 MHz, DMSO-d_6) δ 11.12 (s, 1H), 7.93 (t, J = 5.8 Hz, 1H), 7.81 (m, 1H), 7.50 (d, J = 7.5 Hz, 1H), 7.39 (d, J = 8.6 Hz, 1H), 6.76 (t, J = 5.7 Hz, 1H), 5.12 (dd, J = 13.0, 5.5 Hz, 1H), 4.76 (s, 2H), 3.13 (q, J = 6.7 Hz, 2H), 2.94–2.84 (m, 3H), 2.63–2.57 (m, 1H), 2.54–2.51(m, 1H), 2.06–2.01 (m, 1H), 1.45–1.39 (m, 2H), 1.38–1.30 (m, 11H), 1.27–1.17 (m, 8H).

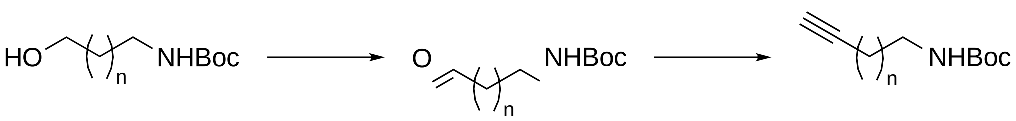

n = 3, 6, or 9

4a–4c

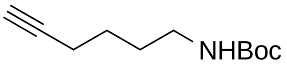

***tert*-Butyl hex-5-yn-1-ylcarbamate (4a).** Oxalyl chloride (0.102 mL, 1.19 mmol) was added to a stirred solution of anhydrous DMSO (93.0 µL, 1.31 mmol) in anhydrous CH_2Cl_2 (1.56 mL) dropwise in a round-bottom flask at –78 ºC, and the mixture was stirred for 5 min. A solution of *tert*-butyl (5-hydroxypentyl)carbamate (156 mg, 0.767 mmol) in anhydrous CH_2Cl_2 (2.35 mL) was added, and then the mixture was stirred at –78 ºC for 15 min. DIPEA (0.519 mL, 2.98 mmol) was added dropwise at –78 ºC. The resulting mixture was warmed to –30 ºC, stirred for 30 min, and concentrated under reduced pressure. The residue was dissolved in ethyl acetate (50 mL) and water (50 mL). The organic

layer was separated, and the aqueous layer was extracted with ethyl acetate (3 × 50 mL). The combined organic layer was washed with aqueous solution of $NaHCO_3$ (5%) (50 mL), water (50 mL), and brine (50 mL), dried over anhydrous Na_2SO_4, filtered, and concentrated under reduced pressure. The crude product was used for the next step without further purification.

Dimethyl (1-diazo-2-oxopropyl)phosphonate (0.138 mL, 0.919 mmol) and K_2CO_3 (234 mg, 1.69 mmol) were added to a stirred solution of the crude *tert*-butyl (5-oxopentyl)carbamate (120 mg, 0.596 mmol) in anhydrous MeOH (4.00 mL) in a 25-mL round-bottom flask at room temperature for 12 h. After completion of the reaction, the reaction mixture was concentrated under reduced pressure, and the resulting residue was dissolved in diethyl ether (50 mL) and water (50 mL). The organic layer was separated, and the aqueous layer was extracted with diethyl ether (3 × 50 mL). The combined organic layer was washed with brine (50 mL), dried over anhydrous Na_2SO_4, filtered, and concentrated under reduced pressure. The product was purified by flash column chromatography. The product was purified by flash column chromatography on silica gel (40% ethyl acetate/hexanes): 75 mg (50% over two steps). Light-yellow oil.

^{1}H NMR (600 MHz, $CDCl_3$) δ 4.55 (br s, 1H), 3.13 (br s, *J* = 6.7 Hz, 2H), 2.21 (td, *J* = 6.8, 2.6 Hz, 2H), 1.94 (t, *J* = 2.6 Hz, 1H), 1.63–1.52 (m, 4H), 1.43 (s, 9H).

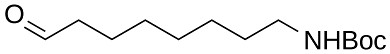

***tert*-Butyl (8-oxooctyl)carbamate.** Pyridinium chlorochromate (276 mg, 1.28 mmol) was added to a solution of *tert*-butyl (8-hydroxyoctyl)carbamate (210 mg, 0.856 mmol) in anhydrous CH_2Cl_2 (10.0 mL) in a 25-mL round-bottom flask. Al_2O_3 (524 mg, 5.14 mmol) was added portionwise over 15 min, and the reaction mixture was stirred at room temperature for 2 h. After completion of the reaction, the mixture was filtered through a pad of Celite®, and the Celite® was washed with CH_2Cl_2. The resulting filtrate was concentrated under reduced pressure. The product was purified by flash column chromatography on silica gel (50% ethyl acetate/hexanes): 175 mg (84%). Light-yellow oil.

^{1}H NMR (600 MHz, $CDCl_3$) δ 9.77–9.76 (m, 1H), 4.51 (br s, 1H), 3.09 (t, *J* = 7.2 Hz, 2H), 2.42 (td, *J* = 6.9, 2.0 Hz, 2H), 1.62 (p, *J* = 7.4 Hz, 2H), 1.50–1.42 (m, 11H), 1.35–1.27 (m, 6H).

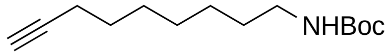

***tert*-Butyl non-8-yn-1-ylcarbamate (4b).** Dimethyl (1-diazo-2-oxopropyl)phosphonate (0.124 mL, 0.826 mmol) and K_2CO_3 (210 mg, 1.52 mmol) were added to a stirred solution of *tert*-butyl (8-oxooctyl)carbamate (170 mg, 0.699 mmol) in anhydrous MeOH (5.00 mL) in a 25-mL round-bottom flask at room temperature for 12 h. After completion of the reaction, the reaction mixture was concentrated under reduced pressure, and the resulting residue was dissolved in diethyl ether (50 mL) and water (50 mL). The organic layer was separated, and the aqueous layer was extracted with diethyl ether (3 × 50 mL). The combined organic layer was washed with brine (50 mL), dried over anhydrous Na_2SO_4, filtered, and concentrated under reduced pressure. The product was purified by flash column chromatography on silica gel (50% ethyl acetate/hexanes): 155 mg (93%). Light-yellow oil.

^{1}H NMR (600 MHz, $CDCl_3$) δ 4.48 (br s, 1H), 3.10 (t, *J* = 7.1 Hz, 2H), 2.18 (td, *J* = 7.1, 2.7 Hz, 2H), 1.93 (t, *J* = 2.6 Hz, 1H), 1.55–1.49 (m, 2H), 1.48–1.42 (s, 11H), 1.42–1.36 (m, 2H), 1.35–1.28 (m, 4H).

***tert*-Butyl (11-oxoundecyl)carbamate.** Pyridinium chlorochromate (146 mg, 0.677 mmol) was added to a solution of *tert*-butyl (11-hydroxyundecyl)carbamate (130 mg, 0.452 mmol) in anhydrous CH_2Cl_2 (8.00 mL) in a 25-mL round-bottom flask. Al_2O_3 (276 mg, 2.71 mmol) was added portionwise over 15 min, and the reaction mixture was stirred at room temperature for 2 h. After completion of the reaction, the mixture was filtered through a pad of Celite®, and the Celite® was washed with CH_2Cl_2. The resulting filtrate was concentrated under reduced pressure. The product was purified by flash column chromatography on silica gel (35% ethyl acetate/hexanes): 64 mg (50%). White solid.
The NMR data were matched to those reported in the literature.[3]

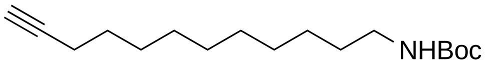

***tert*-Butyl dodec-11-yn-1-ylcarbamate (4c).** K_2CO_3 (103 mg, 0.745 mmol) and dimethyl (1-diazo-2-oxopropyl)phosphonate (60.9 μL, 0.406 mmol) were added to a solution of *tert*-butyl (11-oxoundecyl)carbamate (97 mg, 0.34 mmol) in anhydrous MeOH (4.00 mL) in a 25-mL round-bottom flask at room temperature. The reaction mixture was stirred at room temperature for 16 h. After completion of the reaction, the reaction mixture was concentrated under reduced pressure, and the resulting residue was dissolved in diethyl ether (25 ml) and water (25 ml). The organic layer was separated, and the aqueous layer was extracted with diethyl ether (3 × 25 mL). The combined organic layer was washed with brine (25 mL), dried over anhydrous Na_2SO_4, filtered, and concentrated under reduced pressure. The product was purified by flash column chromatography on silica gel (40% ethyl acetate /hexanes): 64 mg (67%). Light-yellow oil.
^{1}H NMR (600 MHz, $CDCl_3$) δ 4.46 (br s, 1H), 3.08 (br s, 2H), 2.17 (td, *J* = 7.1, 2.6 Hz, 2H), 1.92 (t, *J* = 2.6 Hz, 1H), 1.54–1.48 (m, 2H), 1.47–1.41 (m, 11H), 1.40–1.33 (m, 2H), 1.27 (m, 10H).

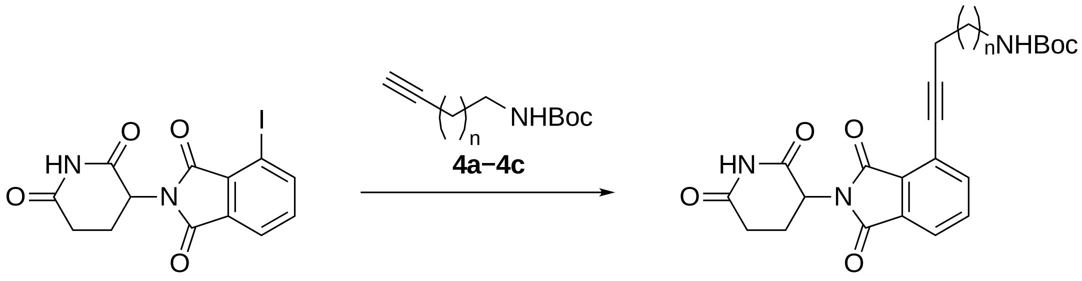

3j–3l

General procedure 5 (GP-5). The alkyne (1.20 equiv), CuI (0.20 equiv), and Et_3N (1.20 equiv) were added to a stirred solution of 2-(2,6-dioxopiperidin-3-yl)-4-iodoisoindoline-1,3-dione (1.00 equiv; 0.15 M) in anhydrous DMF in a round-bottom flask. The reaction mixture was degassed with N_2, followed by the addition of $PdCl_2(PPh_3)_2$ (0.100 equiv). The reaction mixture was stirred at 80 °C for 16 h. After completion of the reaction, the reaction mixture was cooled to room temperature and poured into a saturated aqueous solution of NH_4Cl. The resulting mixture was extracted with ethyl acetate. The combined organic layer was washed with a saturated aqueous solution of LiCl and brine, dried over anhydrous Na_2SO_4, filtered, and concentrated under reduced pressure. The product was purified by flash column chromatography.

(3) Amatore, M.; Beeson, T. D.; Brown, S. P.; MacMillan, D. W. C. *Angew. Chem., Int. Ed.* **2009**, *48*, 5121–5124.

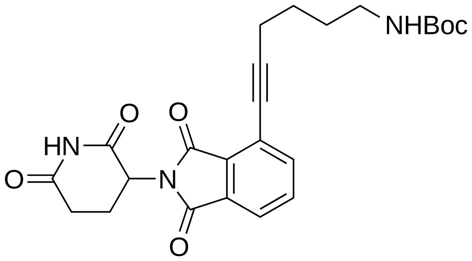

***tert*-Butyl (6-(2-(2,6-dioxopiperidin-3-yl)-1,3-dioxoisoindolin-4-yl)hex-5-yn-1-yl)carbamate (3j).** The title compound was synthesized from *tert*-butyl hex-5-yn-1-ylcarbamate (**4a**, 70 mg, 0.35 mmol) and 2-(2,6-dioxopiperidin-3-yl)-4-iodoisoindoline-1,3-dione (113 mg, 0.294 mmol) according to **GP-5**. The product was purified by flash column chromatography on silica gel (30%→60% acetone/hexanes) and then on C-18 silica gel (10%→70% MeCN/water containing 0.1% formic acid): 66 mg (49%). Yellow solid.

^{1}H NMR (600 MHz, DMSO-d_6) δ 11.11 (s, 1H), 7.87–7.85 (m, 1H), 7.84–7.80 (m, 2H), 6.81 (m, 1H), 5.13 (dd, J = 12.9, 5.5 Hz, 1H), 3.00–2.93 (m, 2H), 2.89 (ddd, J = 17.2, 14.0, 5.5 Hz, 1H), 2.61–2.58 (m, 1H), 2.57–2.51 (m, 3H), 2.08–2.03 (m, 1H), 1.60–1.55 (m, 4H) 1.37 (s, 9H).

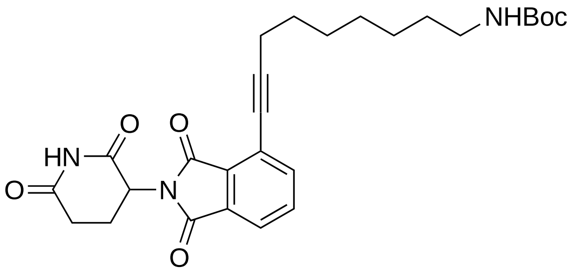

***tert*-Butyl (9-(2-(2,6-dioxopiperidin-3-yl)-1,3-dioxoisoindolin-4-yl)non-8-yn-1-yl)carbamate (3k).** The title compound was synthesized from *tert*-butyl non-8-yn-1-ylcarbamate (**4b**, 97 mg, 0.41 mmol) and 2-(2,6-dioxopiperidin-3-yl)-4-iodoisoindoline-1,3-dione (130 mg, 0.338 mmol) according to **GP-5**. The product was purified by flash column chromatography on silica gel (30%→60% acetone/hexanes): 110 mg (66%). Yellow solid.

^{1}H NMR (600 MHz, DMSO-d_6) δ 11.13 (s, 1H), 7.86 (dd, J = 6.2, 2.3 Hz, 1H), 7.84–7.79 (m, 2H), 6.75 (t, J = 5.6 Hz, 1H), 5.14 (dd, J = 12.9, 5.4 Hz, 1H), 2.94–2.84 (m, 3H), 2.62–2.58 (m, 1H), 2.57–2.51 (m, 3H), 2.08–2.02 (m, 1H), 1.58 (p, J = 7.1 Hz, 2H), 1.48–1.42 (m, 2H), 1.41–1.33 (m, 11H), 1.33–1.22 (m, 4H).

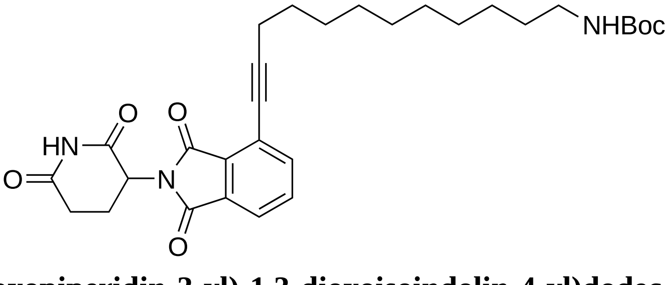

***tert*-Butyl (12-(2-(2,6-dioxopiperidin-3-yl)-1,3-dioxoisoindolin-4-yl)dodec-11-yn-1-yl)carbamate (3l).** The title compound was synthesized from *tert*-butyl dodec-11-yn-1-ylcarbamate (55 mg, 0.20 mmol) and 2-(2,6-dioxopiperidin-3-yl)-4-iodoisoindoline-1,3-dione (63 mg, 0.16 mmol) according to **GP-5**. The product was purified by flash column chromatography on silica gel (10%→40% acetone/hexanes): 66 mg (75%). Yellow solid.

^{1}H NMR (600 MHz, DMSO-d_6) δ 11.13 (s, 1H), 7.86 (dd, *J* = 6.6, 1.9 Hz, 1H), 7.84–7.79 (m, 2H), 6.74 (t, *J* = 5.7 Hz, 1H), 5.14 (dd, *J* = 12.9, 5.4 Hz, 1H), 2.93–2.84 (m, 3H), 2.61–2.58 (m, 1H), 2.56–2.51 (m, 3H), 2.10–2.02 (m, 1H), 1.58 (p, *J* = 7.1 Hz, 2H), 1.46 (p, *J* = 7.1 Hz, 2H), 1.39–1.30 (m, 11H), 1.29–1.17 (m, 10H).

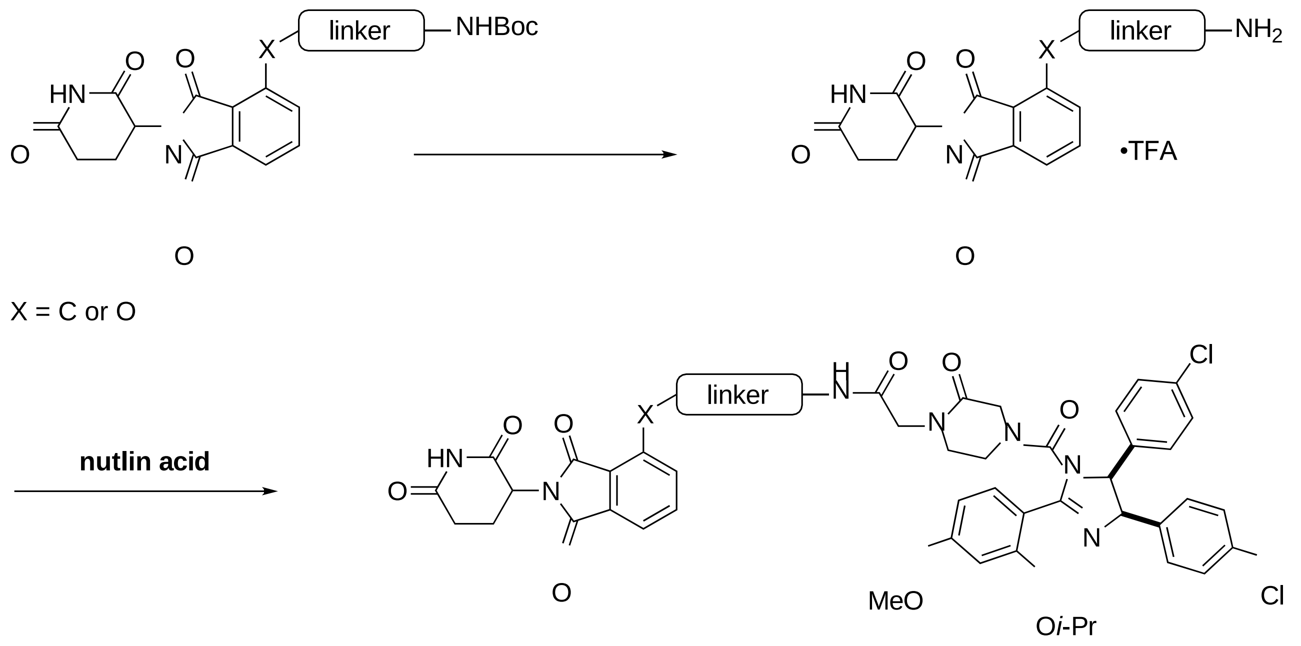

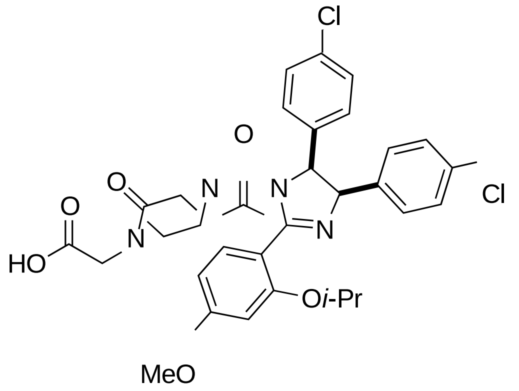

nutlin acid

General Procedure 6 (GP-6). To a stirred solution of the Boc-protected amine in anhydrous CH_2Cl_2 was added TFA dropwise at 0 ºC in a round-bottom flask. The reaction mixture was stirred at room temperature for 1 h. After completion of the reaction, the mixture was concentrated under reduced pressure. The resulting residue was suspended in toluene and subjected to concentration, with this suspension-concentration cycle repeated two times. The amine TFA salt was used for the next step without further purification, assuming a quantitative yield.

Nutlin acid was prepared according to literature procedures.[4] HATU and DIPEA were added to a stirred solution of nutlin acid in anhydrous DMF at room temperature. The reaction mixture was stirred for 30 min at the same temperature, followed by the dropwise addition of the amine TFA salt in anhydrous DMF. The reaction mixture was stirred at room temperature for 16 h. After the completion of the reaction, the mixture was diluted with water and ethyl acetate and stirred vigorously. The resulting mixture was extracted with ethyl acetate. The combined organic layer was washed with a saturated aqueous solution of LiCl and brine, dried over Na_2SO_4, filtered, and concentrated under reduced pressure. The product was purified by flash column chromatography.

---

(4) Schneekloth, A. R.; Pucheault, M.; Tae, H. S.; Crews, C. M. *Bioorg. Med. Chem. Lett.* **2008**, *18*, 5904–5908.

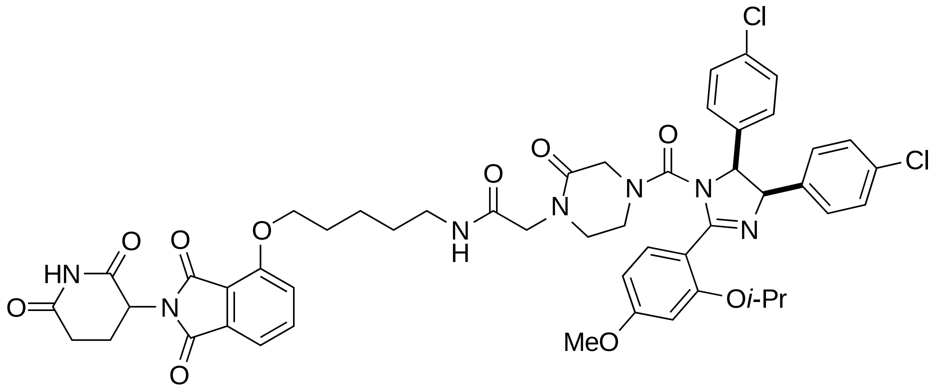

2-(4-(*cis*-4,5-Bis(4-chlorophzenyl)-2-(2-isopropoxy-4-methoxyphenyl)-4,5-dihydro-1*H*-imidazole-1-carbonyl)-2-oxopiperazin-1-yl)-N-(5-((2-(2,6-dioxopiperidin-3-yl)-1,3-dioxoisoindolin-4-yl)oxy)pentyl)acetamide (CL131). The title compound was synthesized from *tert*-butyl (5-((2-(2,6-dioxopiperidin-3-yl)-1,3-dioxoisoindolin-4-yl)oxy)pentyl)carbamate (**3a**) according to **GP-6**. Boc deprotection was conducted with *N*-Boc amine **3a** (50 mg, 0.11 mmol) and TFA (0.600 mL, 7.84 mmol) in anhydrous CH_2Cl_2 (3.00 mL). The resulting amine TFA salt was reacted with nutlin acid (77 mg, 0.12 mmol), HATU (55 mg, 0.14 mmol), and DIPEA (70.1 µL, 0.402 mmol) in anhydrous DMF (7.00 mL). The desired product was purified by column chromatography on C-18 silica gel (10%→ 100% MeCN/water containing 0.1% formic acid) and then preparative HPLC on C-18 silica gel (10% →100% MeCN/water containing 0.1% formic acid): 13 mg (12% over two steps). White solid.
^{1}H NMR (600 MHz, CD_3OD) δ 7.77 (ddd, *J* = 8.5, 7.2, 2.1 Hz, 1H), 7.58 (dd, *J* = 8.3, 3.0 Hz, 1H), 7.43 (t, *J* = 7.8 Hz, 2H), 7.14 (dd, *J* = 8.5, 2.1 Hz, 2H), 7.08 (d, *J* = 8.1 Hz, 2H), 7.03 (dt, *J* = 8.4, 1.8 Hz, 2H), 6.95 (d, *J* = 8.0 Hz, 2H), 6.69–6.63 (m, 2H), 5.75 (d, *J* = 10.1 Hz, 1H), 5.57 (d, *J* = 9.9 Hz, 1H), 5.10 (ddd, *J* = 12.5, 5.3, 1.2 Hz, 1H), 4.75 (p, *J* = 6.0 Hz, 1H), 4.22 (dd, *J* = 6.8, 4.8 Hz, 2H), 4.02–3.72 (m, 7H), 3.52 (d, *J* = 16.7 Hz, 1H), 3.44–3.36 (m, 1H), 3.23 (s, 2H), 3.05 (d, *J* = 19.9 Hz, 2H), 2.89–2.80 (m, 1H), 2.78–2.66 (m, 2H), 2.14–2.05 (m, 1H), 1.86 (s, 2H), 1.58 (s, 4H), 1.40–1.36 (m, 3H), 1.36–1.32 (m, 3H).
^{13}C NMR (151 MHz, CD_3OD) δ 174.7, 174.6, 171.59, 171.56, 169.7, 168.6, 167.5, 167.3, 165.2, 163.3, 158.6, 158.0, 155.7, 138.0, 137.4, 136.3, 135.1, 134.3, 133.2, 130.7, 130.0, 129.1, 129.0, 120.5, 118.1, 116.4, 113.4, 106.5, 101.2, 72.4, 71.8, 70.3, 70.1, 56.2, 50.6, 50.4, 50.3, 48.1, 43.2, 40.13, 40.10, 32.2, 29.8, 29.5, 24.2, 23.7, 22.5, 22.4.
HRMS (ESI) *m/z* ($[M+H]^+$) cacld for $C_{50}H_{52}Cl_2N_7O_{10}$: 980.3153, found:.980.3150.

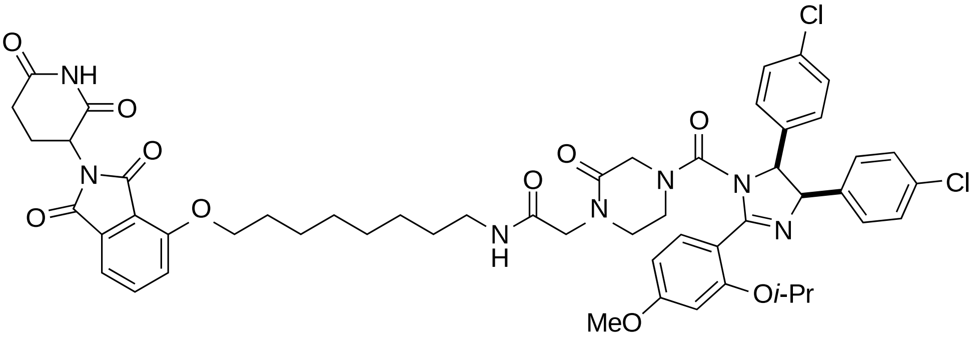

2-(4-(*cis*-4,5-Bis(4-chlorophenyl)-2-(2-isopropoxy-4-methoxyphenyl)-4,5-dihydro-1*H*-imidazole-1-carbonyl)-2-oxopiperazin-1-yl)-*N*-(8-((2-(2,6-dioxopiperidin-3-yl)-1,3-dioxoisoindolin-4-yl)oxy)octyl)acetamide (CL144). The title compound was synthesized from *tert*-butyl (8-((2-(2,6-dioxopiperidin-3-yl)-1,3-dioxoisoindolin-4-yl)oxy)octyl)carbamate (**3b**) according to **GP-6**. Boc

deprotection was conducted with *N*-Boc amine **3b** (18 mg, 0.036 mmol) and TFA (0.300 mL, 3.92 mmol) in anhydrous CH_2Cl_2 (0.771 mL). The resulting amine TFA salt was reacted with nutlin acid (30 mg, 0.047 mmol), HATU (21 mg, 0.055 mmol), and DIPEA (48.5 μL, 0.278 mmol) in anhydrous DMF (2.00 mL). The desired product was purified by column chromatography on C-18 silica gel (10%→100% MeCN/water) and then preparative HPLC on C-18 silica gel (40%→100% MeCN/water): 17 mg (46% over two steps). Light-yellow solid.

^{1}H NMR (600 MHz, CD_3OD) δ 7.78–7.74 (m, 1H), 7.58 (dd, *J* = 8.4, 1.3 Hz, 1H), 7.43 (dd, *J* = 7.5, 3.2 Hz, 2H), 7.15–7.12 (m, 2H), 7.08 (d, *J* = 7.7 Hz, 2H), 7.03 (d, *J* = 8.3 Hz, 2H), 6.94 (d, *J* = 8.0 Hz, 2H), 6.69–6.64 (m, 2H), 5.75 (d, *J* = 10.1 Hz, 1H), 5.56 (d, *J* = 10.1 Hz, 1H), 5.09 (dd, *J* = 12.7, 5.5 Hz, 1H), 4.73 (p, *J* = 6.1 Hz, 1H), 4.22 (t, *J* = 6.3 Hz, 2H), 3.94–3.75 (m, 7H), 3.56–3.49 (m, 1H), 3.43–3.37 (m, 1H), 3.16 (t, *J* = 7.0 Hz, 2H), 3.10–2.99 (m, 2H), 2.86 (ddd, *J* = 19.0, 13.9, 5.3 Hz, 1H), 2.78–2.66 (m, 2H), 2.13–2.07 (m, 1H), 1.88–1.81 (m, 2H), 1.57–1.44 (m, 4H), 1.41–1.31 (m, 12H).

^{13}C NMR (151 MHz, CD_3OD) δ 174.6, 171.6, 169.6, 168.7, 167.4, 165.0, 163.2, 158.6, 158.1, 155.8, 138.0, 137.6, 136.5, 135.1, 134.2, 133.1, 130.7, 130.0, 129.1, 129.0, 120.5, 118.1, 116.3, 113.8, 106.4, 101.2, 72.4, 72.1, 70.5, 70.1, 56.2, 50.6, 50.4, 50.2, 48.2, 43.2, 40.4, 32.2, 30.3, 30.1, 29.9, 27.7, 26.8, 23.7, 22.5, 22.4.

HRMS (ESI) *m/z* ($[M+H]^+$) cacld for $C_{53}H_{58}Cl_2N_7O_{10}$: 1022.3622, found: 1022.3615.

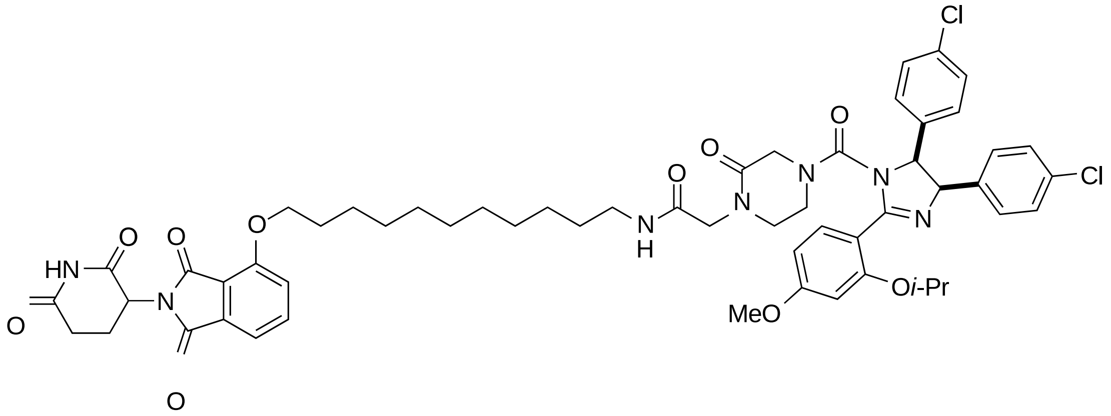

2-(4-(*cis*-4,5-Bis(4-chlorophenyl)-2-(2-isopropoxy-4-methoxyphenyl)-4,5-dihydro-1*H*-imidazole-1-carbonyl)-2-oxopiperazin-1-yl)-*N*-(11-((2-(2,6-dioxopiperidin-3-yl)-1,3-dioxoisoindolin-4-yl)oxy)undecyl)acetamide (CL145). The title compound was synthesized from *tert*-butyl (11-((2-(2,6-dioxopiperidin-3-yl)-1,3-dioxoisoindolin-4-yl)oxy)undecyl)carbamate (**3c**) according to **GP-6**. Boc deprotection was conducted with *N*-Boc amine **3c** (13 mg, 0.024 mmol) and TFA (0.217 mL, 2.84 mmol) in anhydrous CH_2Cl_2 (0.650 mL). The resulting amine TFA salt was reacted with nutlin acid (20 mg, 0.031 mmol), HATU (14 mg, 0.037 mmol), and DIPEA (32.3 μL, 0.185 mmol) in anhydrous DMF (2.00 mL). The desired product was purified by column chromatography on C-18 silica gel (10%→100% MeCN/water containing 0.1% formic acid) and then preparative HPLC on C-18 silica gel (40%→100% MeCN/water containing 0.1% formic acid): 16 mg (63% over two steps). Light-yellow solid.

^{1}H NMR (600 MHz, CD_3OD) δ 7.76 (dd, *J* = 8.5, 7.3 Hz, 1H), 7.59 (d, *J* = 8.4 Hz, 1H), 7.45–7.40 (m, 2H), 7.16–7.11 (m, 2H), 7.11–7.06 (m, 2H), 7.06–7.01 (m, 2H), 6.95 (d, *J* = 8.2 Hz, 2H), 6.70–6.64 (m, 2H), 5.76 (d, *J* = 10.1 Hz, 1H), 5.57 (d, *J* = 10.1 Hz, 1H), 5.10 (dd, *J* = 12.6, 5.5 Hz, 1H), 4.74 (p, *J* = 6.1 Hz, 1H), 4.22 (t, *J* = 6.4 Hz, 2H), 3.94–3.77 (m, 7H), 3.56–3.50 (m, 1H), 3.43–3.40 (m, 1H), 3.15 (t, *J* = 7.1 Hz, 2H), 3.10–3.01 (m, 2H), 2.86 (ddd, *J* = 17.7, 13.9, 5.4 Hz, 1H), 2.78–2.66 (m, 2H), 2.15–2.07 (m, 1H), 1.85 (dt, *J* = 14.1, 6.5 Hz, 2H), 1.56–1.44 (m, 4H), 1.43–1.26 (m, 18H).

^{13}C NMR (151 MHz, CD_3OD) δ 174.6, 171.5, 169.6, 168.7, 167.4, 165.0, 163.2, 158.5, 158.1, 155.8, 137.9, 137.6, 136.5, 135.1, 134.2, 133.1, 130.7, 130.0, 129.1, 129.0, 120.5, 118.1, 116.3, 113.8, 106.4, 101.2, 72.4, 72.1, 70.5, 70.1, 56.2, 50.5, 50.4, 50.2, 48.2, 43.2, 40.5, 32.2, 30.6, 30.5, 30.4, 30.33, 30.30, 30.0, 27.9, 26.9, 23.7, 22.5, 22.4.
HRMS (ESI) *m/z* ([M+H]$^+$) cacld for $C_{56}H_{64}Cl_2N_7O_{10}$: 1064.4092, found: 1064.4088.

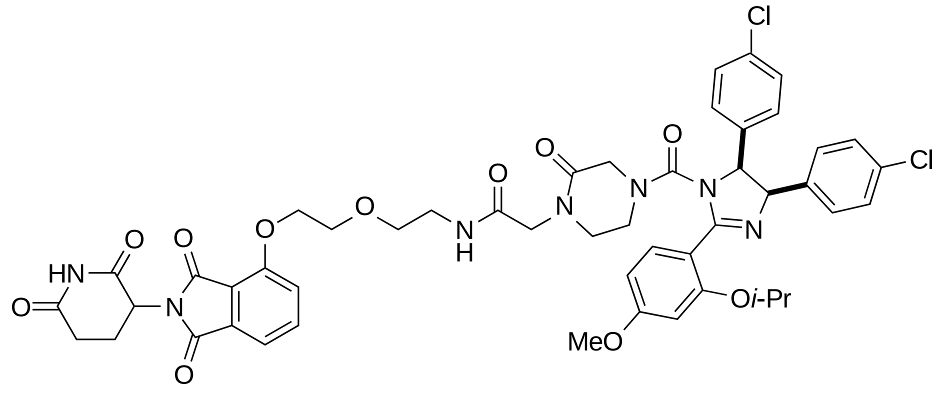

2-(4-(*cis*-4,5-Bis(4-chlorophenyl)-2-(2-isopropoxy-4-methoxyphenyl)-4,5-dihydro-1*H*-imidazole-1-carbonyl)-2-oxopiperazin-1-yl)-*N*-(2-(2-((2-(2,6-dioxopiperidin-3-yl)-1,3-dioxoisoindolin-4-yl)oxy)ethoxy)ethyl)acetamide (CL136). The title compound was synthesized from *tert*-butyl (2-(2-((2-(2,6-dioxopiperidin-3-yl)-1,3-dioxoisoindolin-4-yl)oxy)ethoxy)ethyl)carbamate (**3d**) according to **GP-6**. Boc deprotection was conducted with *N*-Boc amine **3d** (52 mg, 0.11 mmol) and TFA (0.596 mL, 7.79 mmol) in anhydrous CH_2Cl_2 (2.99 mL). The resulting amine TFA salt was reacted with nutlin acid (83 mg, 0.13 mmol), HATU (57 mg, 0.15 mmol), and DIPEA (76.8 μL, 0.441 mmol) in anhydrous DMF (7.80 mL). The desired product was purified by column chromatography on amine silica gel (7% MeOH/CH_2Cl_2) and then preparative HPLC on C-18 silica gel (10%→100% MeCN/water containing 0.1% formic acid): 30 mg (27% over two steps). Light-yellow solid.
^{1}H NMR (600 MHz, CD_3OD) δ 7.79 (dd, *J* = 8.5, 7.3 Hz, 1H), 7.58 (dd, *J* = 8.3, 1.7 Hz, 1H), 7.50–7.44 (m, 2H), 7.13 (d, *J* = 7.9 Hz, 2H), 7.10–7.06 (m, 2H), 7.02 (dd, *J* = 176.4, 8.4 Hz, 2H), 6.95 (d, *J* = 8.0 Hz, 2H), 6.69–6.63 (m, 2H), 5.74 (dd, *J* = 10.0, 2.4 Hz, 1H), 5.57 (dd, *J* = 10.0, 6.8 Hz, 1H), 5.10 (dd, *J* = 12.4, 4.9 Hz, 1H), 4.77–4.70 (m, 1H), 4.37–4.32 (m, 2H), 3.92–3.70 (m, 9H), 3.65 (td, *J* = 5.3, 1.4 Hz, 2H), 3.54–3.45 (m, 1H), 3.45–3.35 (m, 3H), 3.09–2.97 (m, 2H), 2.90–2.79 (m, 1H), 2.76–2.67 (m, 2H), 2.13–2.05 (m, 1H), 1.38 (dd, *J* = 6.0, 1.0 Hz, 3H), 1.34 (dd, *J* = 6.0, 2.0 Hz, 3H).
^{13}C NMR (151 MHz, CD_3OD) δ 174.61, 174.56, 171.5, 169.9, 168.5, 167.7, 167.4, 165.1, 163.2, 158.6, 157.8, 155.9, 138.1, 137.6, 136.6, 136.5, 135.1, 134.2, 133.2, 130.7, 130.0, 129.1, 129.0, 120.8, 118.3, 116.8, 106.4, 101.2, 72.3, 72.1, 70.6, 70.3, 70.2, 69.9, 56.2, 50.5, 50.3, 48.1, 43.2, 40.4, 32.21, 32.18, 23.6, 22.5, 22.4.
HRMS (ESI) *m/z* ([M+H]$^+$) cacld for $C_{49}H_{50}Cl_2N_7O_{11}$: 982.2945, found: 982.2957.

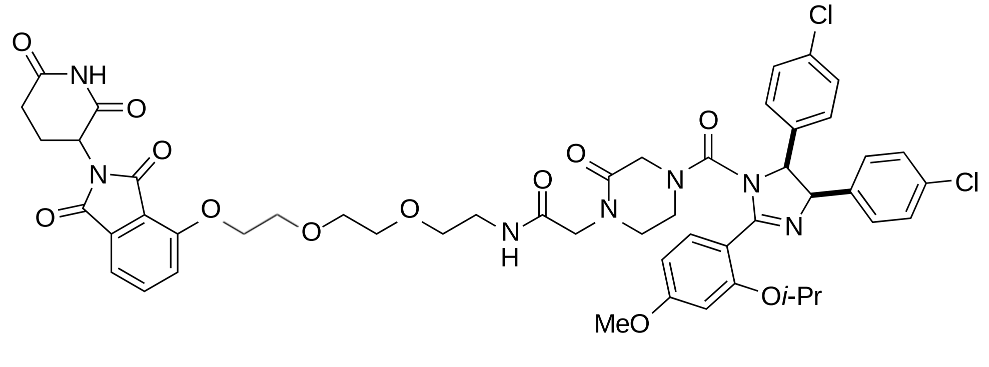

2-(4-(*cis*-4,5-Bis(4-Chlorophenyl)-2-(2-isopropoxy-4-methoxyphenyl)-4,5-dihydro-1*H*-imidazole-1-carbonyl)-2-oxopiperazin-1-yl)-*N*-(2-(2-(2-((2-(2,6-dioxopiperidin-3-yl)-1,3-dioxoisoindolin-4-yl)oxy)ethoxy)ethoxy)ethyl)acetamide (CL138). The title compound was synthesized from *tert*-butyl (2-(2-(2-((2-(2,6-dioxopiperidin-3-yl)-1,3-dioxoisoindolin-4-yl)oxy)ethoxy)ethoxy)ethyl)carbamate (**3e**) according to **GP-6**. Boc deprotection was conducted with *N*-Boc amine **3e** (10 mg, 0.020 mmol) and TFA (0.333 mL, 4.35 mmol) in anhydrous CH_2Cl_2 (1.000 mL). The resulting amine TFA salt was reacted with nutlin acid (20 mg, 0.031 mmol), HATU (14 mg, 0.037 mmol), and DIPEA (32.3 μL, 0.185 mmol) in anhydrous DMF (1.50 mL). The desired product was purified by column chromatography on C-18 silica gel (10%→100% MeCN/water) and then preparative HPLC on C-18 silica gel (40%→100% MeCN/water): 10 mg (49% over two steps). White solid.

^{1}H NMR (600 MHz, CD_3CN) δ 9.06 (s, 1H), 7.74 (dd, *J* = 8.5, 7.3 Hz, 1H), 7.53 (dd, *J* = 8.4, 1.7 Hz, 1H), 7.43 (d, *J* = 7.3 Hz, 1H), 7.39 (d, *J* = 8.5 Hz, 1H), 7.14–7.10 (m, 2H), 7.06 (d, *J* = 8.6 Hz, 2H), 7.03 (d, *J* = 8.1 Hz, 2H), 6.96 (d, *J* = 8.2 Hz, 2H), 6.62–6.55 (m, 3H), 5.62 (d, *J* = 9.9 Hz, 1H), 5.52 (d, *J* = 10.0 Hz, 1H), 4.96 (dd, *J* = 12.5, 5.4 Hz, 1H), 4.69 (p, *J* = 6.0 Hz, 1H), 4.33–4.29 (m, 2H), 3.87–3.75 (m, 7H), 3.71–3.62 (m, 4H), 3.58–3.52 (m, 2H), 3.49–3.39 (m, 3H), 3.31–3.21 (m, 3H), 3.01–2.91 (m, 2H), 2.79–2.60 (m, 3H), 2.11–2.06 (m, 1H) 1.31–1.28 (m, 6H).

^{13}C NMR (151 MHz, CD_3OD) δ 174.7, 174.6, 171.5, 169.9, 168.6, 167.4, 165.0, 163.2, 158.5, 157.8, 155.8, 138.0, 137.6, 136.5, 135.2, 134.2, 133.1, 130.7, 130.0, 129.1, 129.0, 120.9, 118.3, 116.7, 113.8, 106.4, 101.2, 72.4, 72.1, 71.3, 70.45, 70.40, 70.1, 56.2, 50.6, 50.5, 50.2, 48.1, 43.2, 40.5, 32.2, 23.7, 22.5, 22.4.

HRMS (ESI) *m/z* ($[M+H]^+$) cacld for $C_{51}H_{54}Cl_2N_7O_{12}$: 1026.3208, found: 1026.3213.

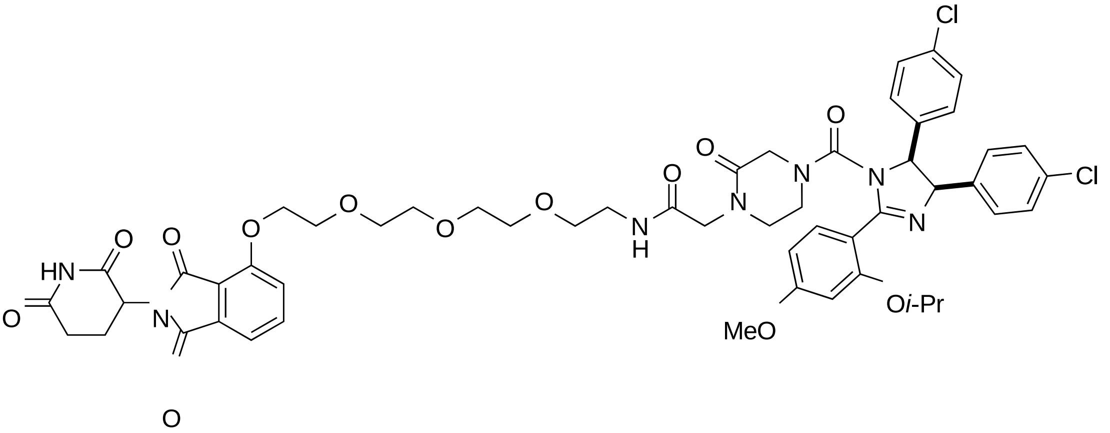

2-(4-(*cis*-4,5-Bis(4-chlorophenyl)-2-(2-isopropoxy-4-methoxyphenyl)-4,5-dihydro-1*H*-imidazole-1-carbonyl)-2-oxopiperazin-1-yl)-*N*-(2-(2-(2-(2-((2-(2,6-dioxopiperidin-3-yl)-1,3-dioxoisoindolin-4-yl)oxy)ethoxy)ethoxy)ethoxy)ethyl)acetamide (CL140). The title compound was synthesized from *tert*-butyl (2-(2-(2-(2-((2-(2,6-dioxopiperidin-3-yl)-1,3-dioxoisoindolin-4-yl)oxy)ethoxy)ethoxy)ethoxy)ethyl)carbamate (**3f**) according to **GP-6**. Boc deprotection was conducted with *N*-Boc amine **3f** (12 mg, 0.022 mmol) and TFA (0.514 mL, 6.72 mmol) in anhydrous CH_2Cl_2 (0.514 mL). The resulting amine TFA salt was reacted with nutlin acid (20 mg, 0.031 mmol), HATU (14 mg, 0.037 mmol), and DIPEA (32.3 μL, 0.185 mmol) in anhydrous DMF (1.50 mL). The desired product was purified by column chromatography on C-18 silica gel (10%→75% MeCN/water containing 0.1% formic acid) and then preparative HPLC on C-18 silica gel (40%→100% MeCN/water containing 0.1% formic acid): 6.7 mg (29% over two steps). White solid.

^{1}H NMR (600 MHz, CD_3CN) δ 9.13 (d, *J* = 5.8 Hz, 1H), 7.74 (dd, *J* = 8.5, 7.3 Hz, 1H), 7.53 (d, *J* = 8.4 Hz, 1H), 7.42 (d, *J* = 7.3 Hz, 1H), 7.38 (d, *J* = 8.5 Hz, 1H), 7.13–7.09 (m, 2H), 7.08–7.05 (m, 2H),

7.05–7.01 (m, 2H), 6.96 (d, J = 8.2 Hz, 2H), 6.63 (t, J = 5.7 Hz, 1H), 6.60–6.55 (m, 2H), 5.61 (dd, J = 9.9, 1.7 Hz, 1H), 5.51 (d, J = 9.9 Hz, 1H), 4.96 (dd, J = 12.6, 5.4 Hz, 1H), 4.69 (p, J = 6.0 Hz, 1H), 4.35–4.26 (m, 2H), 3.85–3.75 (m, 7H), 3.70–3.61 (m, 4H), 3.59–3.55 (m, 2H), 3.54–3.49 (m, 4H), 3.42 (t, J = 5.6 Hz, 3H), 3.30–3.20 (m, 3H), 3.00–2.91 (m, 2H), 2.80–2.62 (m, 3H), 2.12–2.06 (m, 1H), 1.31 (d, J = 6.0 Hz, 3H), 1.27 (d, J = 6.0 Hz, 3H).

^{13}C NMR (151 MHz, CD_3OD) δ 174.6, 171.5, 169.9, 168.6, 167.34, 167.28, 165.0, 163.2, 158.6, 157.8, 155.8, 138.0, 137.6, 136.5, 135.2, 134.2, 133.1, 130.7, 130.0, 129.1, 129.0, 120.9, 118.3, 116.7, 113.8, 106.5, 101.2, 72.4, 72.1, 72.0, 71.6, 71.3, 70.53, 70.45, 70.3, 70.1, 56.2, 50.5, 50.4, 50.2, 48.1, 43.2, 40.4, 32.2, 23.7, 22.5, 22.4.

HRMS (ESI) *m/z* ([M+H]$^+$) cacld for $C_{53}H_{58}Cl_2N_7O_{13}$: 1070.3470, found: 1070.3466.

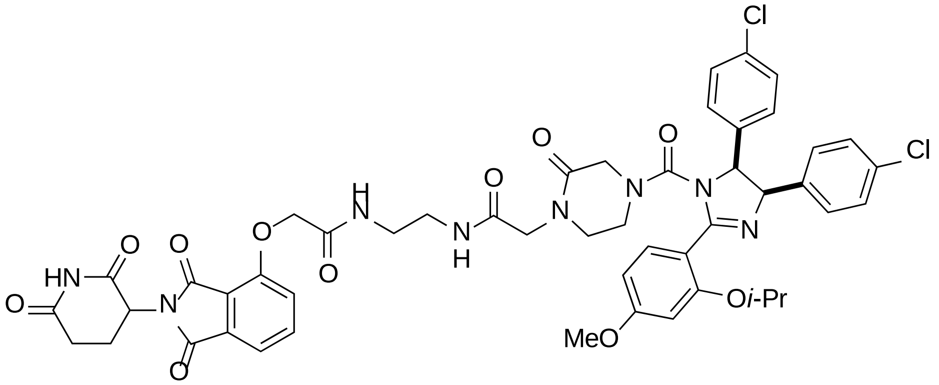

2-(4-(*cis*-4,5-Bis(4-chlorophenyl)-2-(2-isopropoxy-4-methoxyphenyl)-4,5-dihydro-1*H*-imidazole-1-carbonyl)-2-oxopiperazin-1-yl)-*N*-(2-(2-((2-(2,6-dioxopiperidin-3-yl)-1,3-dioxoisoindolin-4-yl)oxy)acetamido)ethyl)acetamide (CL172). The title compound was synthesized from *tert*-butyl (2-(2-((2-(2,6-dioxopiperidin-3-yl)-1,3-dioxoisoindolin-4-yl)oxy)acetamido)ethyl)carbamate (**3g**) according to **GP-6**. Boc deprotection was conducted with *N*-Boc amine **3g** (36 mg, 0.076 mmol) and TFA (0.600 mL, 7.84 mmol) in anhydrous CH_2Cl_2 (0.900 mL). The resulting amine TFA salt was reacted with nutlin acid (66 mg, 0.10 mmol), HATU (47 mg, 0.12 mmol), and DIPEA (88.9 µL, 0.510 mmol) in anhydrous DMF (2.00 mL). The desired product was purified by flash column chromatography on silica gel (1→10% MeOH/CH_2Cl_2) and then on C-18 silica gel (10→60% MeCN/water containing 0.1% formic acid): 52 mg (69% over two steps). White solid.

^{1}H NMR (600 MHz, CD_3OD) δ 7.81 (ddd, J = 8.6, 7.3, 1.3 Hz, 1H), 7.59–7.53 (m, 2H), 7.43 (d, J = 8.5 Hz, 1H), 7.14 (dd, J = 14.2, 5.5 Hz, 2H), 7.08 (d, J = 8.7 Hz, 2H), 7.03 (d, J = 14.1 Hz, 2H), 6.95 (d, J = 8.1 Hz, 2H), 6.69–6.64 (m, 2H), 5.74 (dd, J = 10.1, 2.9 Hz, 1H), 5.57 (dd, J = 10.1, 5.9 Hz, 1H), 5.16 (ddd, J = 12.7, 11.6, 5.6 Hz, 1H), 4.78–4.70 (m, 3H), 4.06–3.70 (m, 7H), 3.53–3.39 (m, 3H), 3.39–3.32 (m, 3H), 3.09–2.96 (m, 2H), 2.92–2.82 (m, 1H), 2.78–2.69 (m, 2H), 2.15–2.09 (m, 1H), 1.38 (d, J = 6.0 Hz, 3H), 1.33 (dd, J = 6.0, 1.3 Hz, 3H).

^{13}C NMR (151 MHz, CD_3OD) δ 174.6, 171.5, 170.5, 170.3, 168.3, 167.9, 167.4, 165.0, 163.2, 158.6, 156.3, 155.9, 138.3, 137.6, 136.5, 134.9, 134.2, 133.2, 130.8, 130.0, 129.1, 129.0, 122.0, 119.4, 118.1, 106.5, 101.2, 72.4, 72.1, 70.1, 69.5, 69.4, 56.2, 50.6, 50.4, 50.3, 48.1, 43.2, 40.3, 39.5, 32.2, 23.6, 22.5, 22.4.

HRMS (ESI) *m/z* ([M+H]$^+$) cacld for $C_{49}H_{49}Cl_2N_8O_{11}$: 995.2898, found: 995.2892.

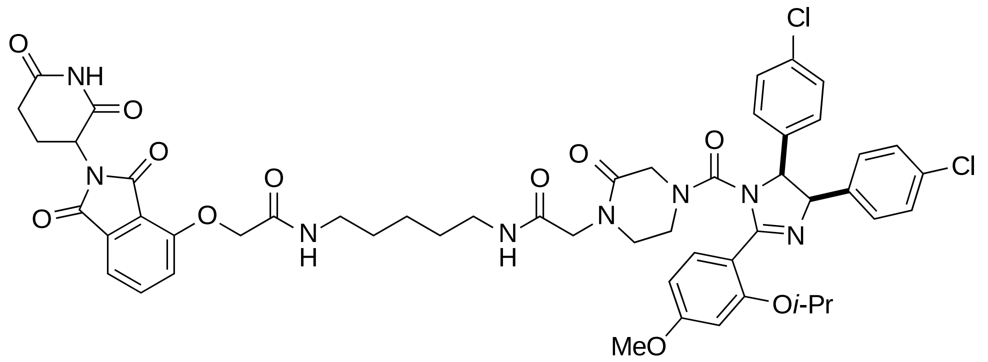

2-(4-(*cis*-4,5-Bis(4-Chlorophenyl)-2-(2-isopropoxy-4-methoxyphenyl)-4,5-dihydro-1*H*-imidazole-1-carbonyl)-2-oxopiperazin-1-yl)-*N*-(5-(2-((2-(2,6-dioxopiperidin-3-yl)-1,3-dioxoisoindolin-4-yl)oxy)acetamido)pentyl)acetamide (CL171). The title compound was synthesized from *tert*-butyl (5-(2-((2-(2,6-dioxopiperidin-3-yl)-1,3-dioxoisoindolin-4-yl)oxy)acetamido)pentyl)carbamate (**3h**) according to **GP-6**. Boc deprotection was conducted with *N*-Boc amine **3h** (58 mg, 0.11 mmol) and TFA (0.773 mL, 10.1 mmol) in anhydrous CH_2Cl_2 (1.16 mL). The resulting amine TFA salt was reacted with nutlin acid (77 mg, 0.12 mmol), HATU (55 mg, 0.14 mmol), and DIPEA (0.104 mL, 0.597 mmol) in anhydrous DMF (2.00 mL). The desired product was purified by flash column chromatography on silica gel (1→10% MeOH/CH_2Cl_2): 70 mg (60% over two steps). White solid.
^{1}H NMR (600 MHz, CD_3CN) δ 9.07 (br s, 1H), 7.77 (dd, J = 8.6, 7.4 Hz, 1H), 7.53–7.49 (m, 2H), 7.36 (d, *J* = 8.4 Hz, 1H), 7.31 (br s, 1H), 7.11 (d, *J* = 8.4 Hz, 2H), 7.05 (t, *J* = 8.7 Hz, 4H), 6.96 (d, *J* = 8.4 Hz, 2H), 6.61–6.56 (m, 2H), 6.35 (br s, 1H), 5.63 (d, *J* = 10.0 Hz, 1H), 5.52 (d, *J* = 10.0 Hz, 1H), 5.01 (dd, *J* = 12.4, 5.6 Hz, 1H), 4.69 (p, *J* = 6.0 Hz, 1H), 4.65 (s, 2H), 3.85 (s, 3H), 3.81–3.76 (m, 2H), 3.71–3.66 (m, 2H), 3.45–3.39 (m, 1H), 3.30–3.21 (m, 3H), 3.15–3.09 (m, 2H), 3.04–2.95 (m, 2H), 2.83–2.75 (m, 1H), 2.74–2.64 (m, 2H), 2.15–2.13 (m, 1H), 1.53 (p, *J* = 6.9 Hz, 2H), 1.46 (p, *J* = 7.1 Hz, 2H), 1.38–1.28 (m, 8H).
^{13}C NMR (151 MHz, CD_3OD) δ 174.6, 171.4, 169.9, 169.7, 168.3, 167.8, 167.4, 165.0, 163.2, 158.6, 156.3, 155.9, 138.3, 137.6, 136.5, 134.9, 134.2, 133.1, 130.7, 130.0, 129.1, 129.0, 121.8, 119.4, 118.0, 113.8, 106.4, 101.2, 72.4, 72.1, 70.1, 69.4, 56.2, 50.6, 50.3, 48.2, 43.2, 40.3, 40.0, 32.2, 29.9, 29.8, 25.1, 23.6, 22.5, 22.4.
HRMS (ESI) *m/z* ($[M+H]^+$) cacld for $C_{52}H_{55}Cl_2N_8O_{11}$: 1037.3367, found: 1037.3364.

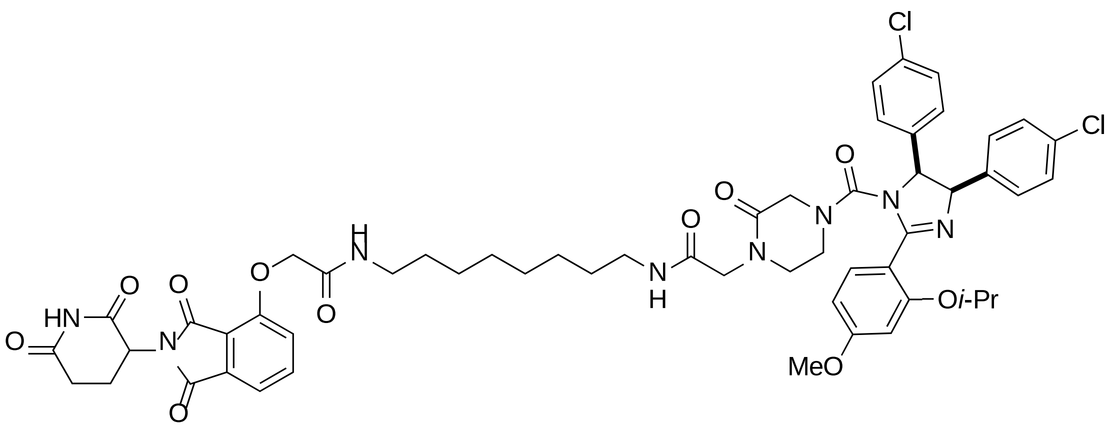

2-(4-(*cis*-4,5-Bis(4-chlorophenyl)-2-(2-isopropoxy-4-methoxyphenyl)-4,5-dihydro-1*H*-imidazole-1-carbonyl)-2-oxopiperazin-1-yl)-*N*-(8-(2-((2-(2,6-dioxopiperidin-3-yl)-1,3-dioxoisoindolin-4-yl)oxy)acetamido)octyl)acetamide (CL173). The title compound was synthesized from *tert*-butyl (8-(2-((2-(2,6-dioxopiperidin-3-yl)-1,3-dioxoisoindolin-4-yl)oxy)acetamido)octyl)carbamate (**3i**) according to **GP-6**. Boc deprotection was conducted with *N*-Boc amine **3i** (58 mg, 0.10 mmol) and

TFA (0.933 mL, 12.2 mmol) in anhydrous CH_2Cl_2 (1.55 mL). The resulting amine TFA salt was reacted with nutlin acid (78 mg, 0.12 mmol), HATU (56 mg, 0.15 mmol), and DIPEA (0.106 mL, 0.609 mmol) in anhydrous DMF (3.00 mL). The desired product was purified by flash column chromatography on silica gel (1→10% MeOH/CH_2Cl_2): 59 mg (53% over two steps). White solid.
^{1}H NMR (600 MHz, CD_3OD) 7.78 (t, *J* = 12.9 Hz, 1H), 7.56 (d, *J* = 8.4 Hz, 1H), 7.51 (dd, *J* = 7.2, 1.5 Hz, 1H), 7.40 (d, *J* = 8.5 Hz, 1H), 7.12–7.09 (m, 2H), 7.07–7.05 (m, 2H), 7.02–6.99 (m, 2H), 6.92 (d, *J* = 8.0 Hz, 2H), 6.69–6.65 (m, 2H), 5.73 (d, *J* = 10.1 Hz, 1H), 5.54 (d, *J* = 10.2 Hz, 1H), 5.11 (dd, *J* = 12.6, 5.4 Hz, 1H), 4.74–4.68 (m, 3H), 3.88–3.78 (m, 7H), 3.54–3.47 (m, 1H), 3.44–3.37 (m, 1H), 3.11 (t, *J* = 7.1 Hz, 2H), 3.08–2.98 (m, 2H), 2.88–2.80 (m, 1H), 2.76–2.66 (m, 2H), 2.14–2.08 (m, 1H), 1.54 (p, *J* = 7.0 Hz, 2H), 1.43 (p, *J* = 7.0 Hz, 2H), 1.37–1.22 (m, 16H).
^{13}C NMR (151 MHz, CD_3OD) δ 174.6, 171.4, 169.8, 169.6, 168.3, 167.8, 167.4, 165.0, 163.2, 158.6, 156.3, 155.8, 138.2, 137.6, 136.5, 134.9, 134.2, 133.1, 130.7, 130.0, 129.1, 129.0, 121.7, 119.3, 118.0, 113.8, 106.5, 101.2, 72.4, 72.1, 70.1, 69.4, 56.2, 50.6, 50.3, 48.2, 43.2, 40.4, 40.1, 32.2, 30.3, 30.2, 27.8, 27.7, 23.6, 22.5, 22.4.
HRMS (ESI) *m/z* ($[M+H]^+$) cacld for $C_{55}H_{61}Cl_2N_8O_{11}$: 1079.3837, found: 1079.3836.

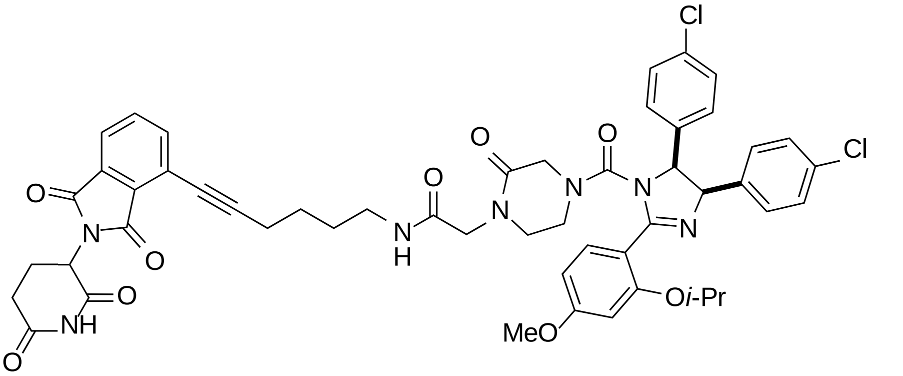

2-(4-(*cis*-4,5-Bis(4-chlorophenyl)-2-(2-isopropoxy-4-methoxyphenyl)-4,5-dihydro-1*H*-imidazole-1-carbonyl)-2-oxopiperazin-1-yl)-*N*-(6-(2-(2,6-dioxopiperidin-3-yl)-1,3-dioxoisoindolin-4-yl)hex-5-yn-1-yl)acetamide (CL175). The title compound was synthesized from *tert*-butyl (6-(2-(2,6-dioxopiperidin-3-yl)-1,3-dioxoisoindolin-4-yl)hex-5-yn-1-yl)carbamate (**3j**) according to **GP-6**. Boc deprotection was conducted with *N*-Boc amine **3j** (49 mg, 0.11 mmol) and TFA (0.817 mL, 10.7 mmol) in anhydrous CH_2Cl_2 (1.63 mL). The resulting amine TFA salt was reacted with nutlin acid (82 mg, 0.13 mmol), HATU (59 mg, 0.16 mmol), and DIPEA (0.112 mL, 0.643 mmol) in anhydrous DMF (2.00 mL). The desired product was purified by flash column chromatography on silica gel (1→10% MeOH/CH_2Cl_2) and then on C-18 silica gel (20%→70% MeCN/water containing 0.1% formic acid): 45 mg (43% over two steps). White solid.
^{1}H NMR (600 MHz, CD_3OD) δ 7.83–7.78 (m, 1H), 7.77–7.72 (m, 2H), 7.58 (dd, *J* = 8.4, 3.7 Hz, 1H), 7.15–7.12 (m, 2H), 7.08 (d, *J* = 8.7 Hz, 2H), 7.05–7.01 (m, 2H), 6.95 (d, *J* = 7.8 Hz, 2H), 6.69–6.64 (m, 2H), 5.75 (d, *J* = 10.2 Hz, 1H), 5.57 (d, *J* = 10.1 Hz, 1H), 5.16 (ddd, *J* = 12.5, 5.4, 2.8 Hz, 1H), 4.73 (p, *J* = 6.0 Hz, 1H), 3.99–3.77 (m, 7H), 3.56–3.49 (m, 1H), 3.45–3.35 (m, 1H), 3.26 (t, *J* = 6.7 Hz, 2H), 3.10–3.02 (m, 2H), 2.91–2.81 (m, 1H), 2.79–2.69 (m, 2H), 2.55 (t, *J* = 6.7 Hz, 2H), 2.16–2.08 (m, 1H), 1.79–1.72 (m, 2H), 1.71–1.63 (m, 2H), 1.38 (d, *J* = 6.0 Hz, 3H), 1.34 (dd, *J* = 6.0, 1.4 Hz, 3H).
^{13}C NMR (151 MHz, CD_3OD) δ 174.6, 171.5, 169.8, 168.0, 167.6, 167.3, 165.0, 163.3, 158.6, 155.8, 139.5, 137.5, 136.4, 135.3, 134.2, 133.8, 133.1, 131.9, 130.7, 130.0, 129.1, 129.0, 123.4, 122.4, 113.7,

106.5, 101.3, 99.6, 77.4, 72.4, 72.0, 70.1, 56.2, 50.6, 50.3, 48.1, 43.2, 39.9, 32.2, 29.4, 26.6, 23.6, 22.5, 22.4, 20.0.
HRMS (ESI) *m/z* ([M+H]$^+$) cacld for $C_{51}H_{50}Cl_2N_7O_9$: 974.3047, found: 974.3057.

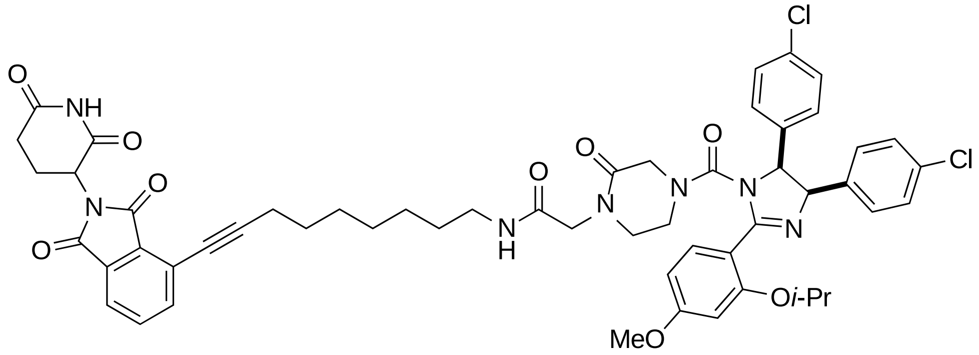

2-(4-(*cis*-4,5-Bis(4-chlorophenyl)-2-(2-isopropoxy-4-methoxyphenyl)-4,5-dihydro-1*H*-imidazole-1-carbonyl)-2-oxopiperazin-1-yl)-*N*-(9-(2-(2,6-dioxopiperidin-3-yl)-1,3-dioxoisoindolin-4-yl)non-8-yn-1-yl)acetamide (CL174). The title compound was synthesized from *tert*-butyl (9-(2-(2,6-dioxopiperidin-3-yl)-1,3-dioxoisoindolin-4-yl)non-8-yn-1-yl)carbamate (**3k**) according to **GP-6**. Boc deprotection was conducted with *N*-Boc amine **3k** (60 mg, 0.12 mmol) and TFA (0.800 mL, 10.5 mmol) in anhydrous CH_2Cl_2 (1.60 mL). The resulting amine TFA salt was reacted with nutlin acid (88 mg, 0.14 mmol), HATU (63 mg, 0.17 mmol), and DIPEA (0.120 mL, 0.689 mmol) in anhydrous DMF (2.50 mL). The desired product was purified by flash column chromatography on silica gel (1→10% MeOH/CH_2Cl_2) and then on C-18 silica gel (10%→60% MeCN/water containing 0.1% formic acid): 36 mg (29% over two steps). White solid.
^{1}H NMR (600 MHz, CD_3OD) δ 7.80 (dd, *J* = 6.4, 2.1 Hz, 1H), 7.76–7.72 (m, 2H), 7.58 (dd, *J* = 8.4, 2.2 Hz, 1H), 7.14 (d, *J* = 8.4 Hz, 2H), 7.08 (d, *J* = 8.8 Hz, 2H), 7.03 (d, *J* = 7.1 Hz, 2H), 6.94 (d, *J* = 8.0 Hz, 2H), 6.68–6.64 (m, 2H), 5.75 (d, *J* = 10.1 Hz, 1H), 5.56 (d, *J* = 10.1 Hz, 1H), 5.14 (dd, *J* = 12.7, 5.5 Hz, 1H), 4.73 (p, *J* = 6.0 Hz, 1H), 3.90–3.76 (m, 7H), 3.55–3.49 (m, 1H), 3.39 (m, 1H), 3.16 (t, *J* = 7.0 Hz, 2H), 3.10–2.99 (m, 2H), 2.91–2.83 (m, 1H), 2.78–2.67 (m, 2H), 2.52 (t, *J* = 6.8 Hz, 2H), 2.13 (m, 1H), 1.68–1.62 (m, 2H), 1.59–1.53 (m, 2H), 1.50 (p, *J* = 7.1 Hz, 2H), 1.40–1.32 (m, 10H).
^{13}C NMR (151 MHz, CD_3OD) δ 174.6, 171.5, 169.6, 168.1, 167.5, 167.4, 165.0, 163.2, 158.6, 155.8, 139.5, 137.6, 136.5, 135.3, 134.2, 133.8, 133.1, 131.9, 130.7, 130.0, 129.1, 129.0, 123.3, 122.6, 113.8, 106.5, 101.3, 100.1, 77.1, 72.4, 72.1, 70.1, 56.2, 50.6, 50.2, 48.2, 43.2, 40.4, 32.2, 30.2, 29.8, 29.6, 29.3, 27.8, 23.6, 22.5, 22.4, 20.3.
HRMS (ESI) *m/z* ([M+H]$^+$) cacld for $C_{54}H_{56}Cl_2N_7O_9$:1016.3517, found: 1016.3515.

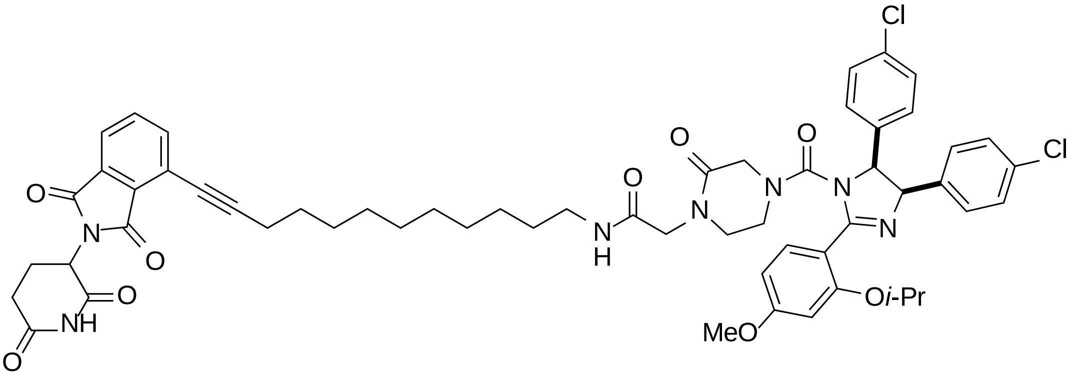

2-(4-(*cis*-4,5-Bis(4-Chlorophenyl)-2-(2-isopropoxy-4-methoxyphenyl)-4,5-dihydro-1*H*-imidazole-1-carbonyl)-2-oxopiperazin-1-yl)-*N*-(12-(2-(2,6-dioxopiperidin-3-yl)-1,3-dioxoisoindolin-4-

yl)dodec-11-yn-1-yl)acetamide (CL176). The title compound was synthesized from *tert*-butyl (12-(2-(2,6-dioxopiperidin-3-yl)-1,3-dioxoisoindolin-4-yl)dodec-11-yn-1-yl)carbamate (**3l**) according to **GP-6**. Boc deprotection was conducted with *N*-Boc amine **3l** (48 mg, 0.089 mmol) and TFA (0.421 mL, 5.50 mmol) in anhydrous CH_2Cl_2 (1.26 mL). The resulting amine TFA salt was reacted with nutlin acid (64 mg, 0.10 mmol), HATU (46 mg, 0.12 mmol), and DIPEA (86.3 µL, 0.495 mmol) in anhydrous DMF (2.00 mL). The desired product was purified by flash column chromatography on silica gel (8% MeOH/CH_2Cl_2) and then on C-18 silica gel (30%→90% MeCN/water containing 0.1% formic acid): 42 mg (44% over two steps). White solid.

^{1}H NMR (600 MHz, CD_3CN) δ 9.10 (s, 1H), 7.80–7.76 (m, 1H), 7.75–7.71 (m, 2H), 7.53 (d, *J* = 8.7 Hz, 1H), 7.14–7.11 (m, 2H), 7.07 (d, *J* = 6.8 Hz, 2H), 7.03 (d, *J* = 8.2 Hz, 2H), 6.96 (d, *J* = 8.1 Hz, 2H), 6.61–6.55 (m, 2H), 6.43 (br s, 1H), 5.62 (d, *J* = 9.9 Hz, 1H), 5.51 (d, *J* = 9.8 Hz, 1H), 5.03–4.97 (m, 1H), 4.75–4.66 (m, 1H), 3.87–3.84 (m, 3H), 3.83–3.75 (m, 2H), 3.65 (d, *J* = 17.4 Hz, 2H), 3.48–3.40 (m, 1H), 3.30–3.20 (m, 1H), 3.09 (q, *J* = 6.8 Hz, 2H), 3.02–2.91 (m, 2H), 2.82–2.63 (m, 3H), 2.55–2.49 (m, 2H), 2.13–2.09 (m, 1H), 1.65–1.59 (m, 2H), 1.55–1.45 (m, 2H), 1.43–1.35 (m, 2H), 1.35–1.20 (m, 16H).

^{13}C NMR (151 MHz, CD_3OD) δ 174.6, 171.4, 169.6, 168.1, 167.4, 167.3, 165.0, 163.2, 158.6, 155.8, 139.5, 137.6, 136.4, 135.3, 134.2, 133.8, 133.1, 131.9, 130.7, 130.0, 129.1, 129.0, 123.3, 122.6, 113.8, 106.5, 101.2, 100.2, 77.1, 72.4, 72.0, 70.1, 56.2, 50.6, 50.2, 48.2, 43.2, 40.4, 32.2, 30.53, 30.50, 30.4, 30.3, 30.2, 29.8, 29.4, 27.9, 23.6, 22.5, 22.4, 20.4.

HRMS (ESI) *m/z* ($[M+H]^+$) cacld for $C_{57}H_{62}C_{l2}N_7O_9$: 1058.3986, found: 1058.3987.

3. NMR Spectra

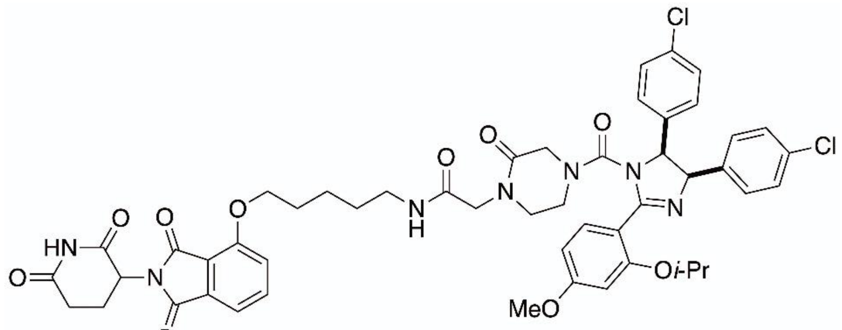

CL0131

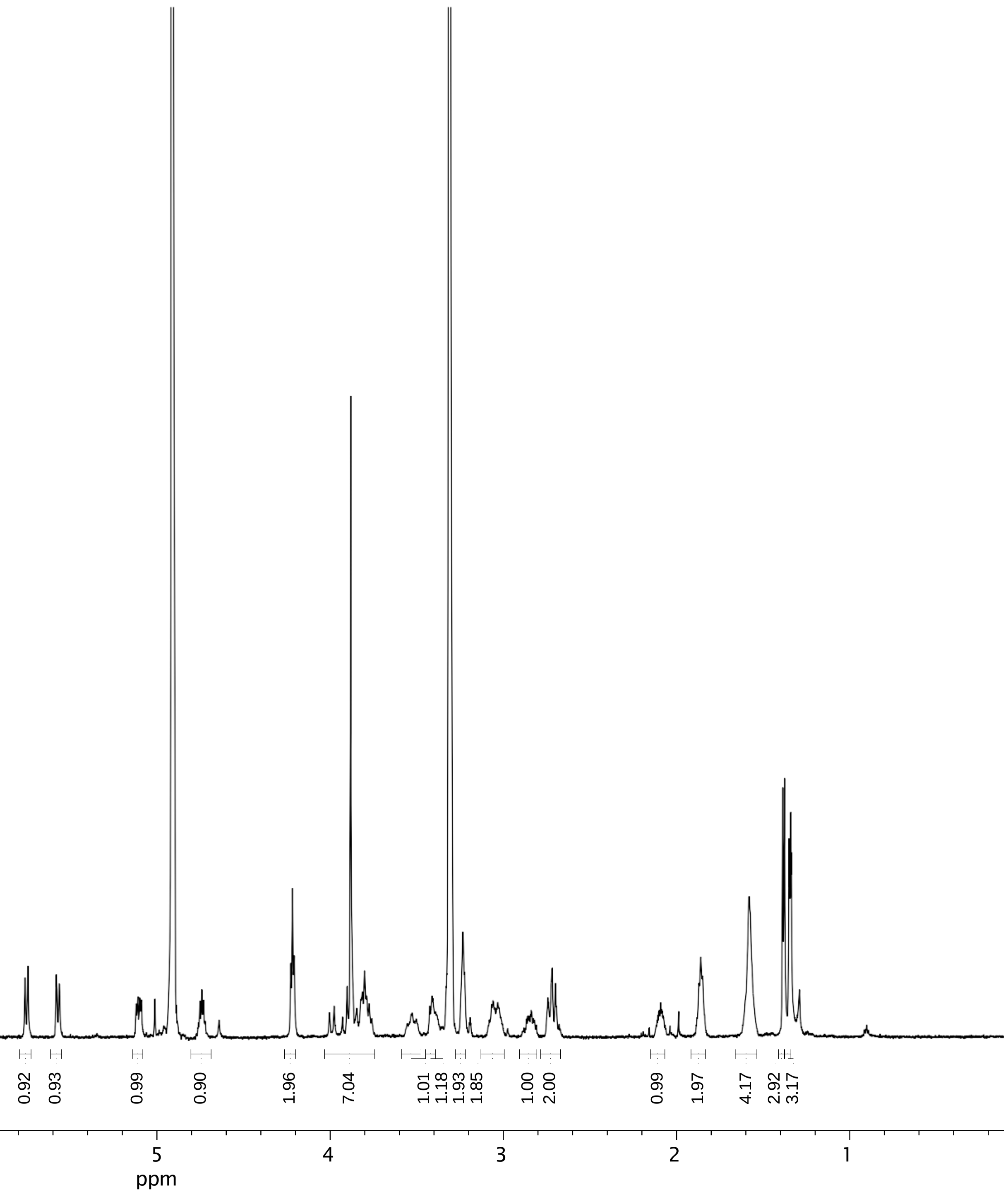

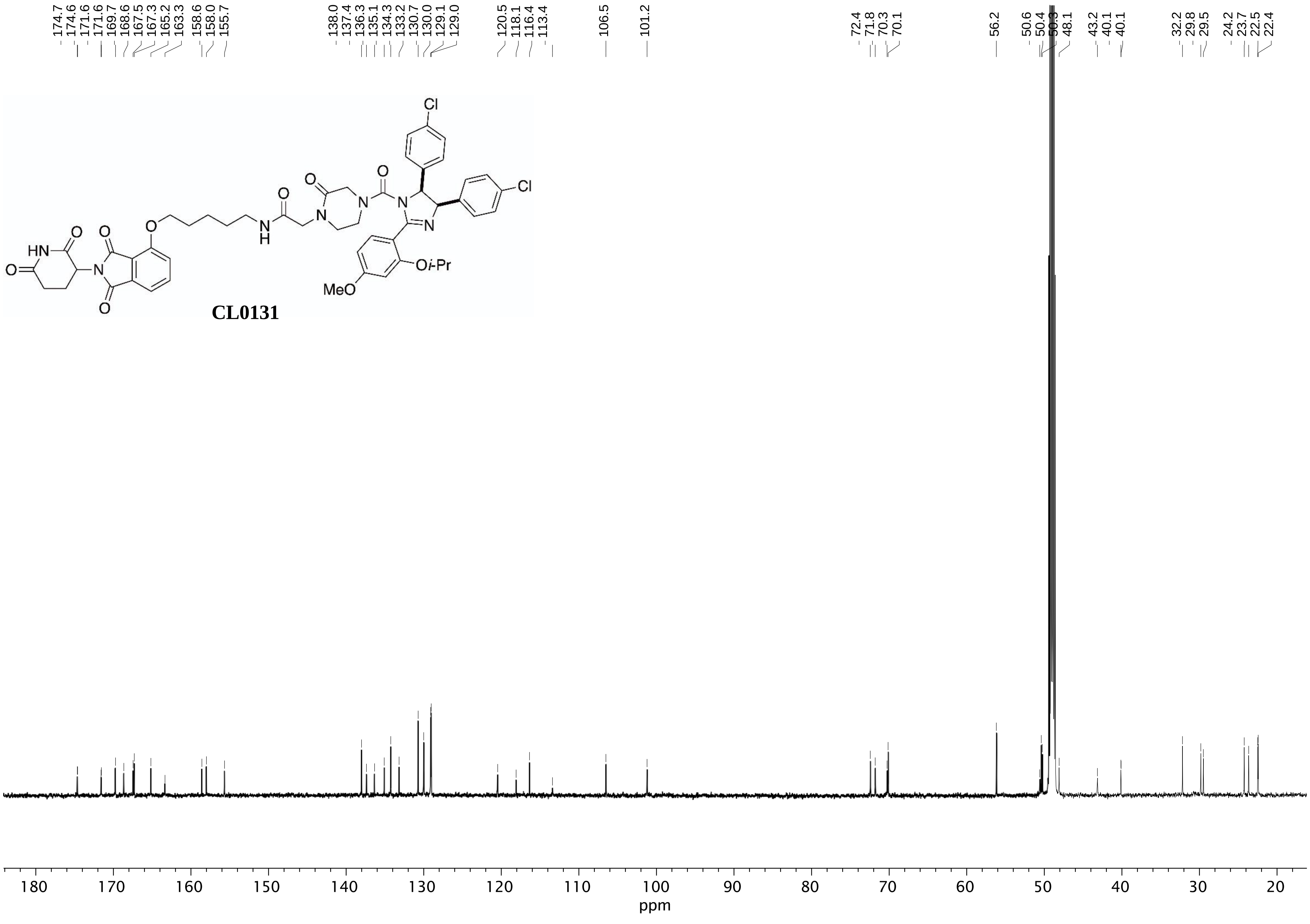

174.7
174.6
171.6
171.6
169.7
168.6
167.5
167.3
165.2
163.3
158.6
158.0
155.7
138.0
137.4
136.3
135.1
134.3
133.2
130.7
130.0
129.1
129.0
120.5
118.1
116.4
113.4
106.5
101.2
72.4
71.8
70.3
70.1
56.2
50.6
50.4
50.3
48.1
43.2
40.1
40.1
32.2
29.8
29.5
24.2
23.7
22.5
22.4
HN
O
N
NH
N
Cl
Oi-Pr
MeO
CL0131
ppm

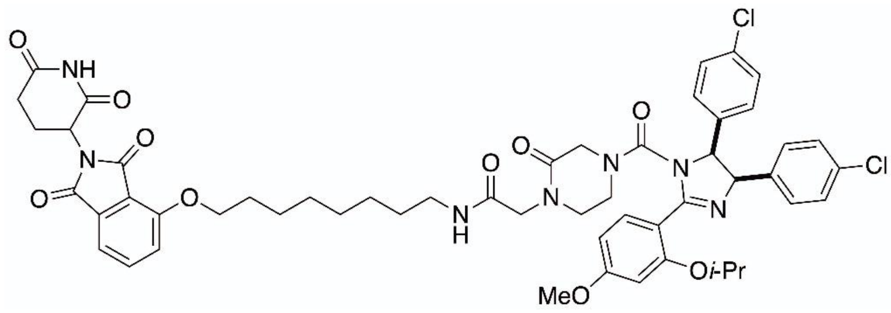

CL0144

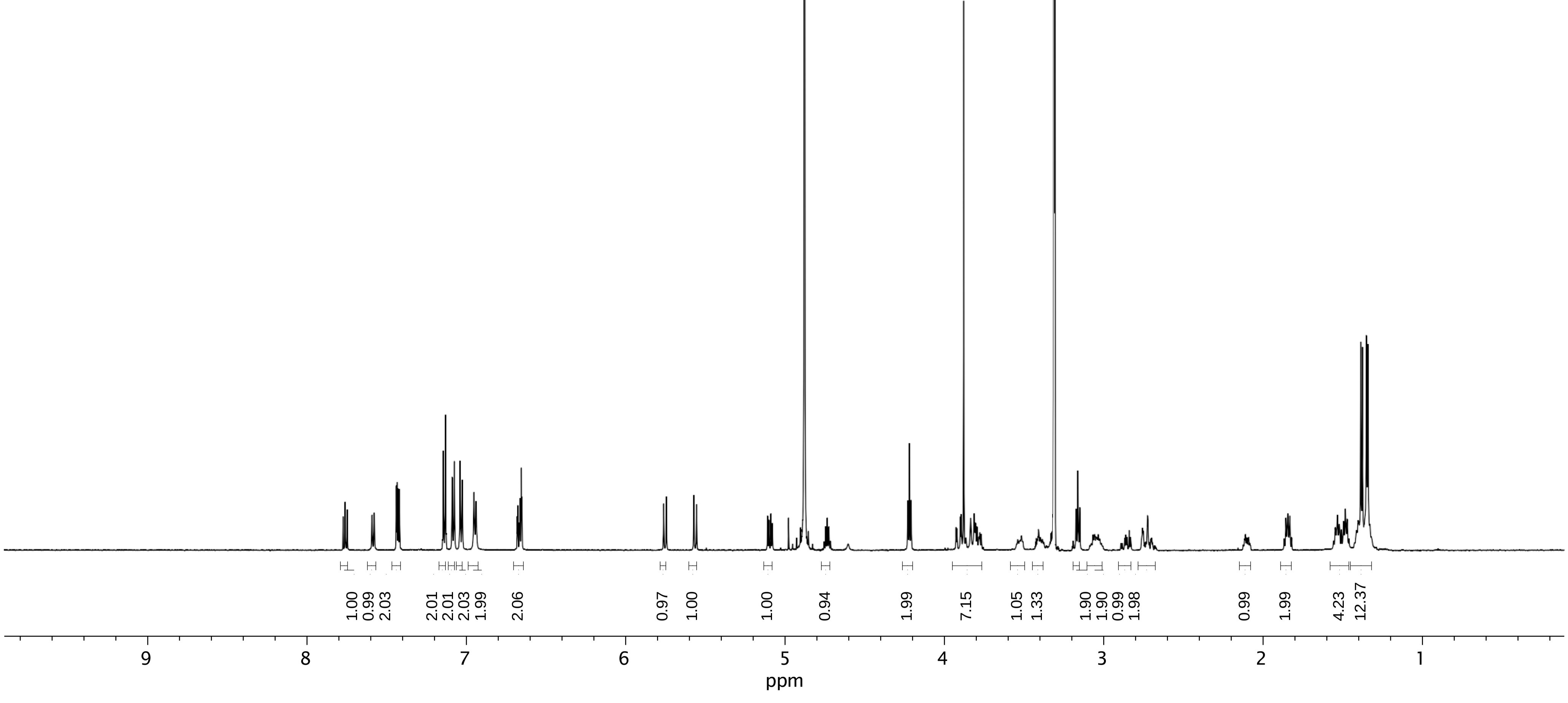
1.00
0.99
2.03
2.01
2.01
2.03
1.99
2.06
0.97
1.00
1.00
0.94
1.99
7.15
1.05
1.33
1.90
1.90
0.99
1.98
0.99
1.99
4.23
12.37
9
8
7
6
5
4
3
2
1
ppm

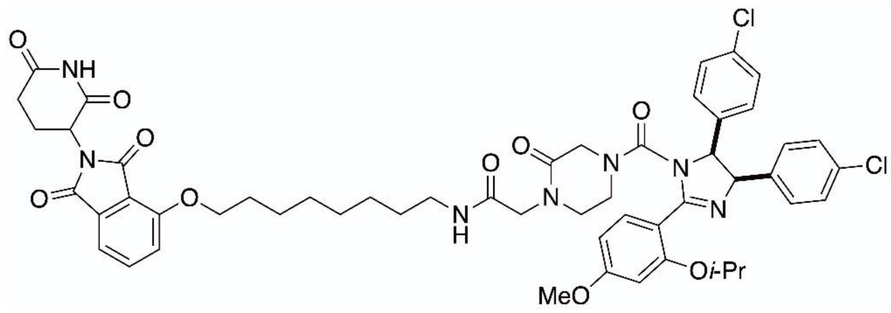
O
NH
O
O
N
O
O
O
N
H
O
N
N
O
N
N
Cl
Cl
MeO
Oi-Pr

CL0144

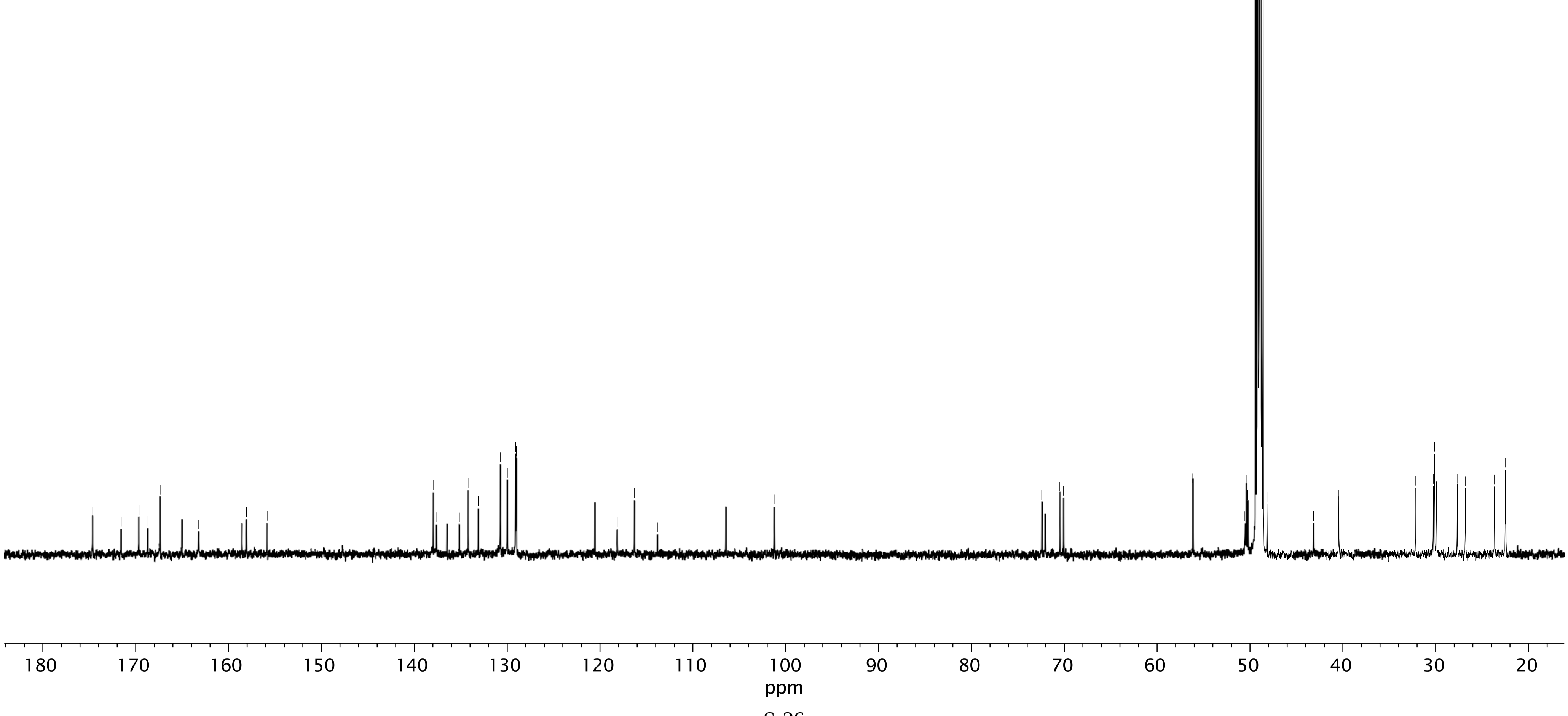
174.6
171.6
169.6
168.7
167.4
165.0
163.2
158.6
158.1
155.8
138.0
137.6
136.5
135.1
134.2
133.1
130.7
130.0
129.1
129.0
120.5
118.1
116.3
113.8
106.4
101.2
72.4
72.1
70.5
70.1
56.2
50.6
50.4
50.2
48.2
43.2
40.4
32.2
30.3
30.1
29.9
27.7
26.8
23.7
22.5
22.4
180
170
160
150
140
130
120
110
100
90
80
70
60
50
40
30
20
ppm

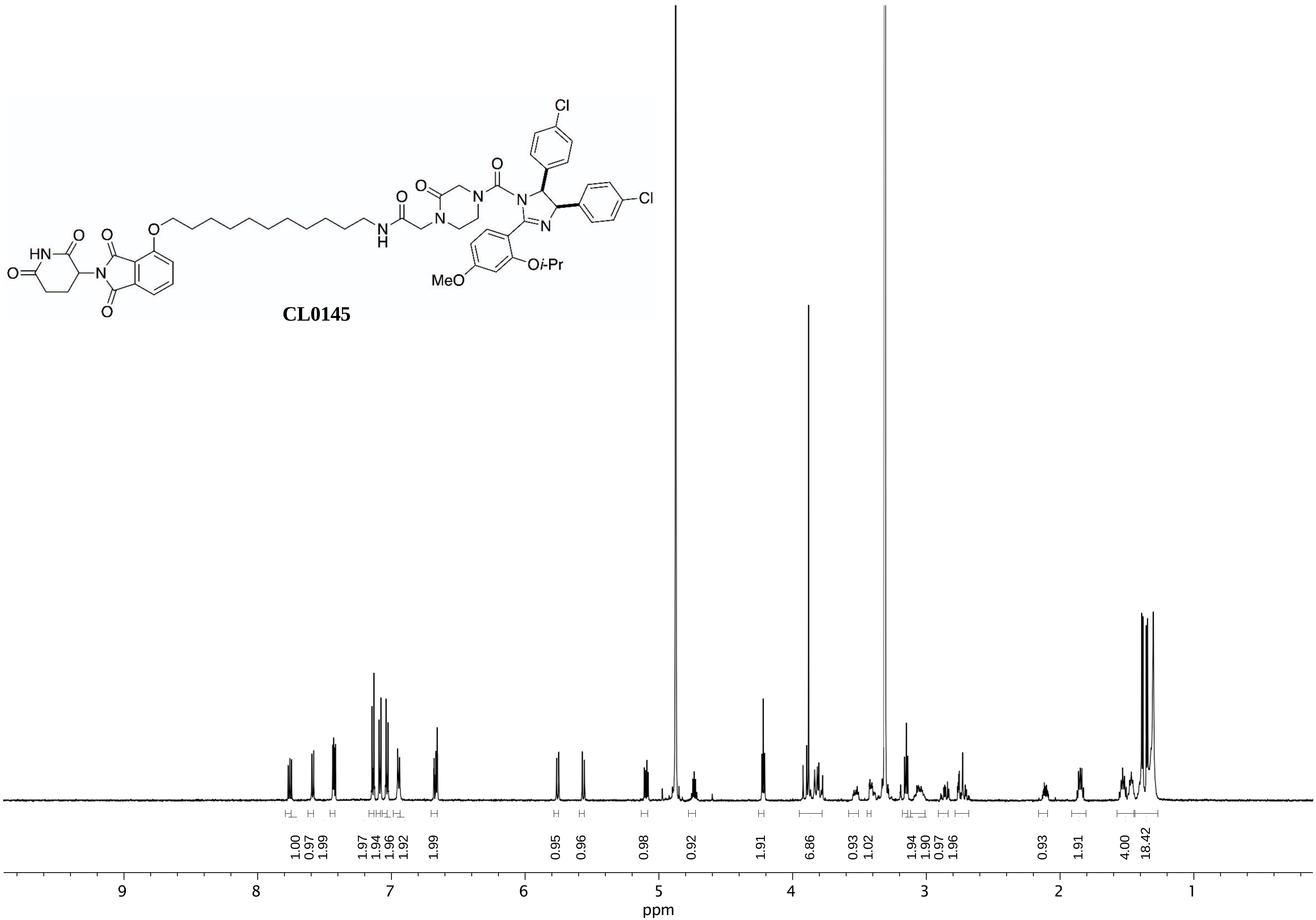

CL0145

1.00
0.97
1.99
1.97
1.94
1.96
1.92
1.99
0.95
0.96
0.98
0.92
1.91
6.86
0.93
1.02
1.94
1.90
0.97
1.96
0.93
1.91
4.00
18.42
9
8
7
6
5
4
3
2
1
ppm

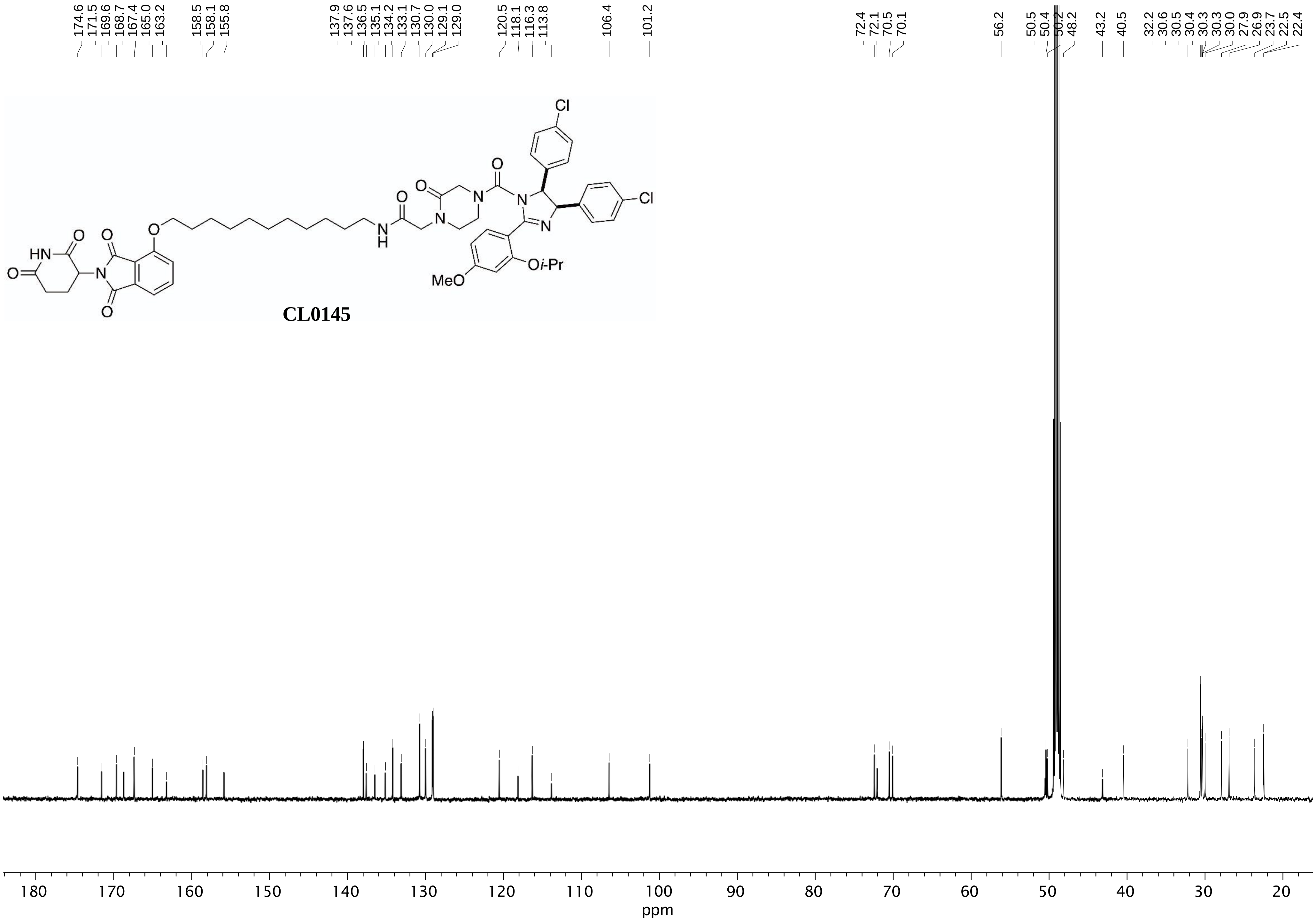
174.6
171.5
169.6
168.7
167.4
165.0
163.2
158.5
158.1
155.8
137.9
137.6
136.5
135.1
134.2
133.1
130.7
130.0
129.1
129.0
120.5
118.1
116.3
113.8
106.4
101.2
72.4
72.1
70.5
70.1
56.2
50.5
50.4
50.2
48.2
43.2
40.5
32.2
30.6
30.5
30.4
30.3
30.3
30.0
27.9
26.9
23.7
22.5
22.4
HN
O
N
Cl
MeO
Oi-Pr
CL0145
180
170
160
150
140
130
120
110
100
90
80
70
60
50
40
30
20
ppm

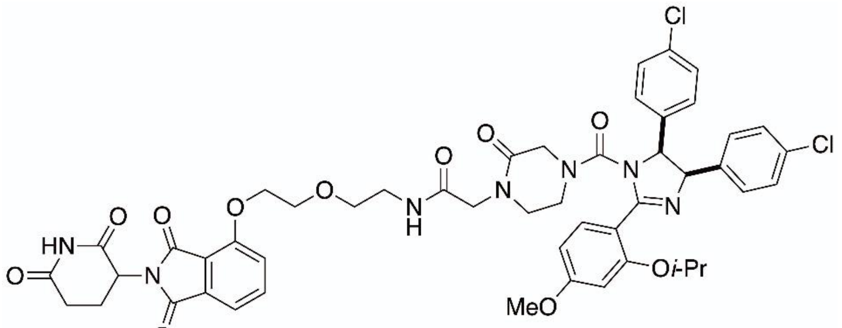

CL0136

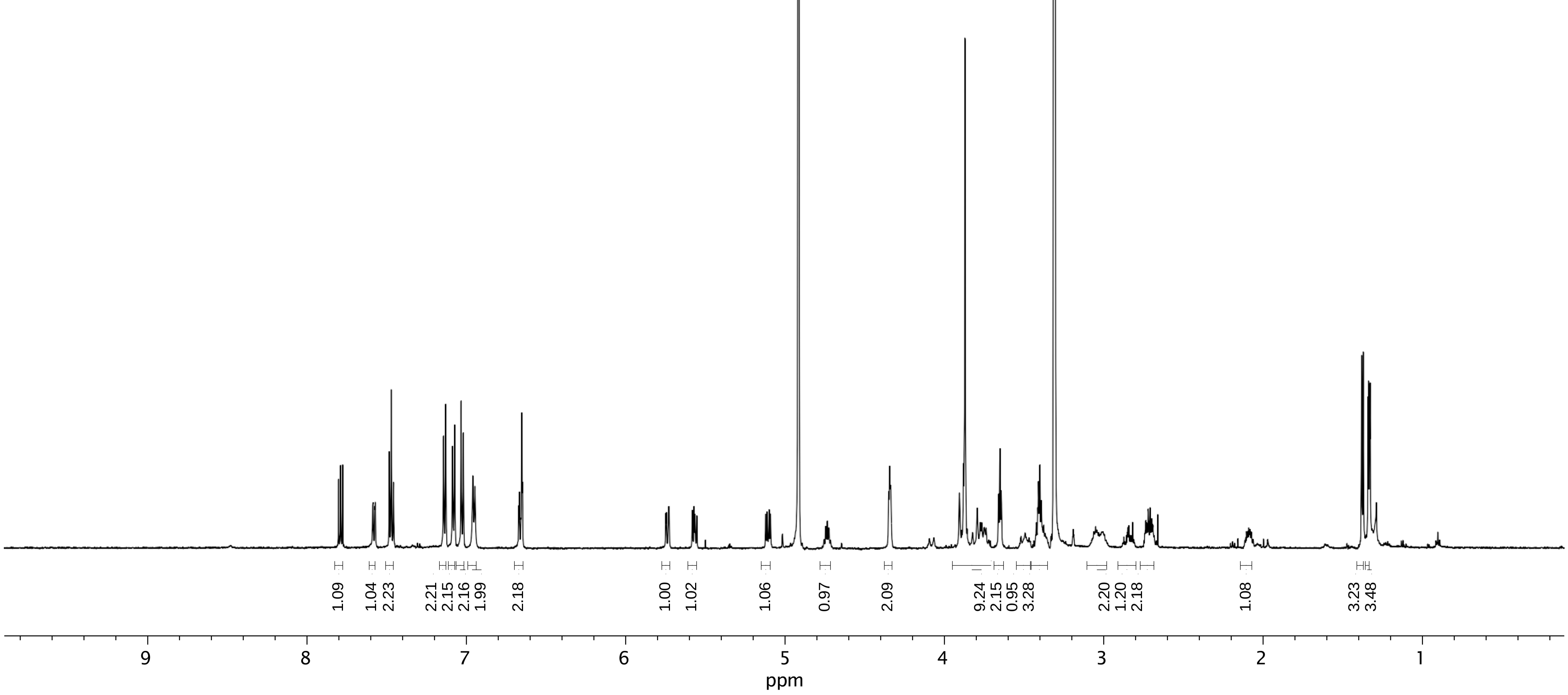

1.09
1.04
2.23
2.21
2.15
2.16
1.99
2.18
1.00
1.02
1.06
0.97
2.09
9.24
2.15
0.95
3.28
2.20
1.20
2.18
1.08
3.23
3.48
9
8
7
6
5
4
3
2
1
ppm

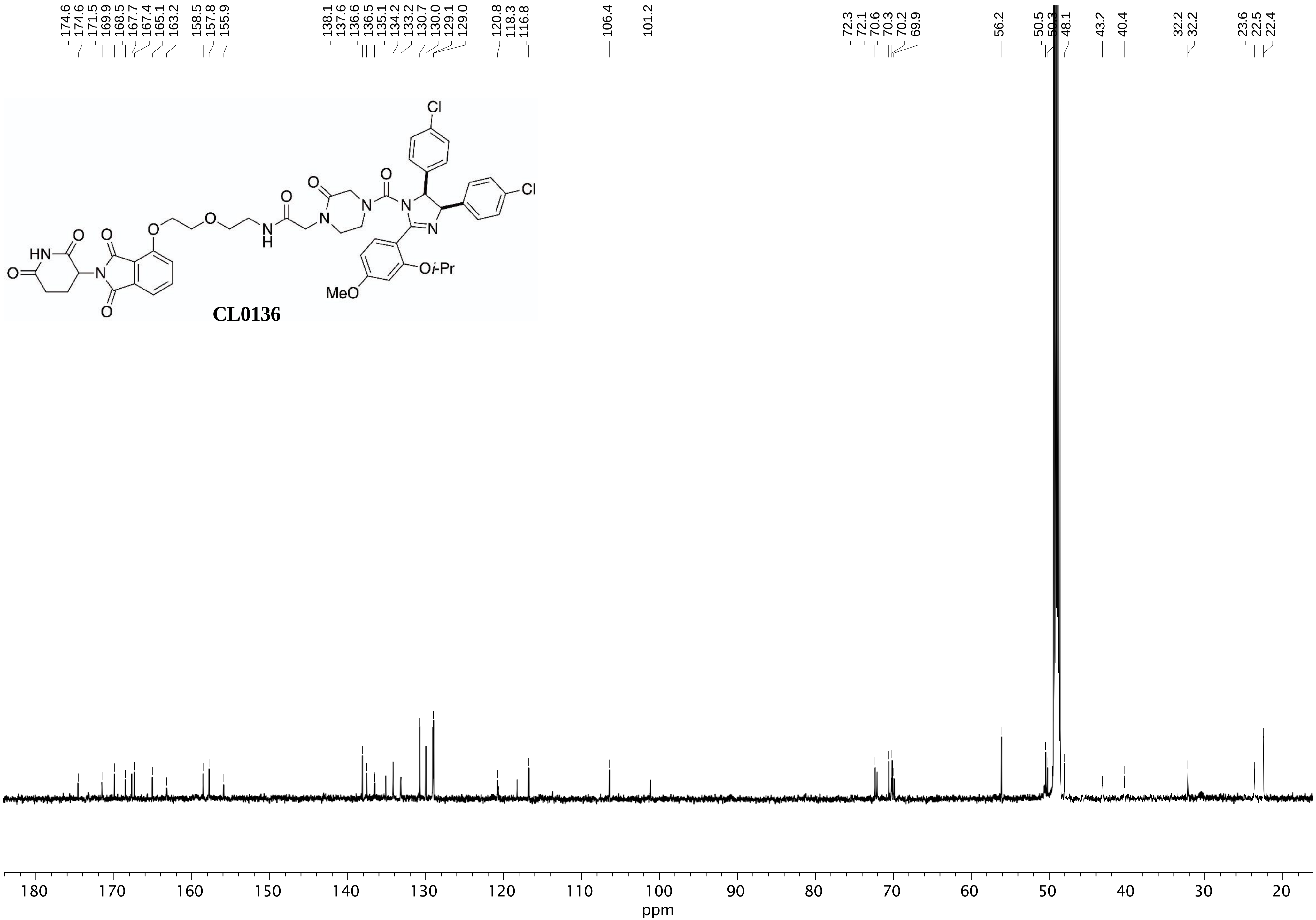

174.6
174.6
171.5
169.9
168.5
167.7
167.4
165.1
163.2
158.5
157.8
155.9
138.1
137.6
136.6
136.5
135.1
134.2
133.2
130.7
130.0
129.1
129.0
120.8
118.3
116.8
106.4
101.2
72.3
72.1
70.6
70.3
70.2
69.9
56.2
50.5
50.3
48.1
43.2
40.4
32.2
32.2
23.6
22.5
22.4
CL0136
180
170
160
150
140
130
120
110
100
90
80
70
60
50
40
30
20
ppm

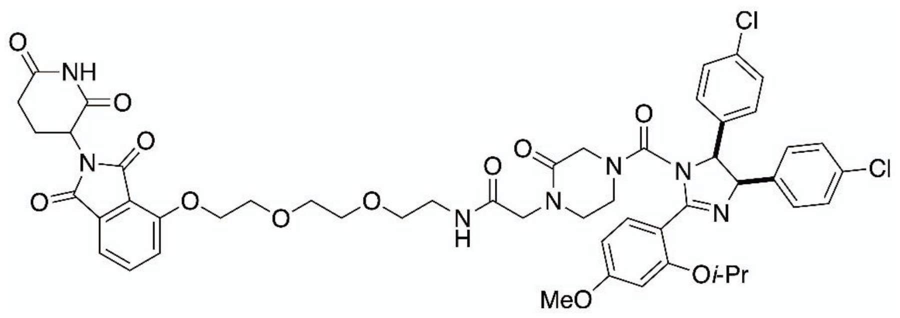

CL0138

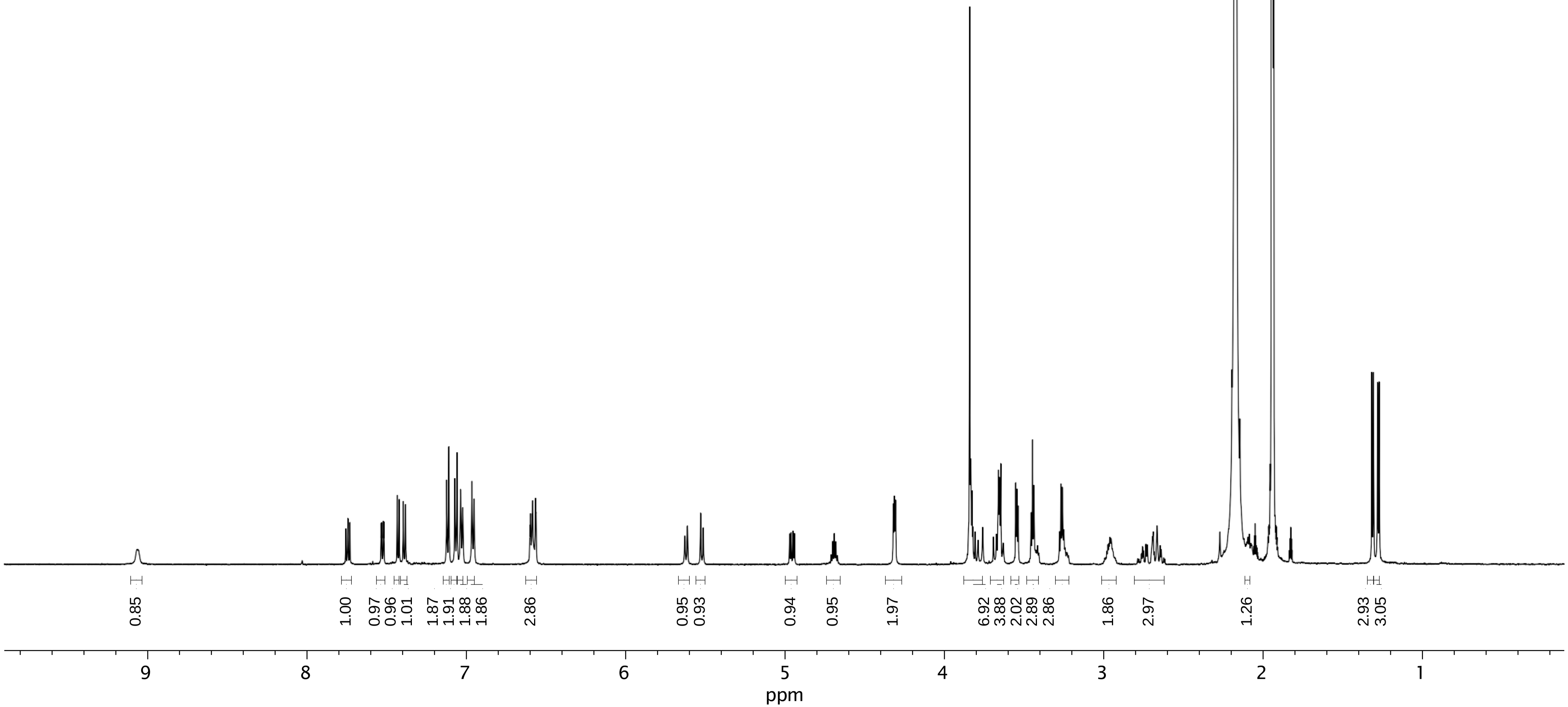
0.85
1.00
0.97
0.96
1.01
1.87
1.91
1.88
1.86
2.86
0.95
0.93
0.94
0.95
1.97
6.92
3.88
2.02
2.89
2.86
1.86
2.97
1.26
2.93
3.05
9
8
7
6
5
4
3
2
1
ppm

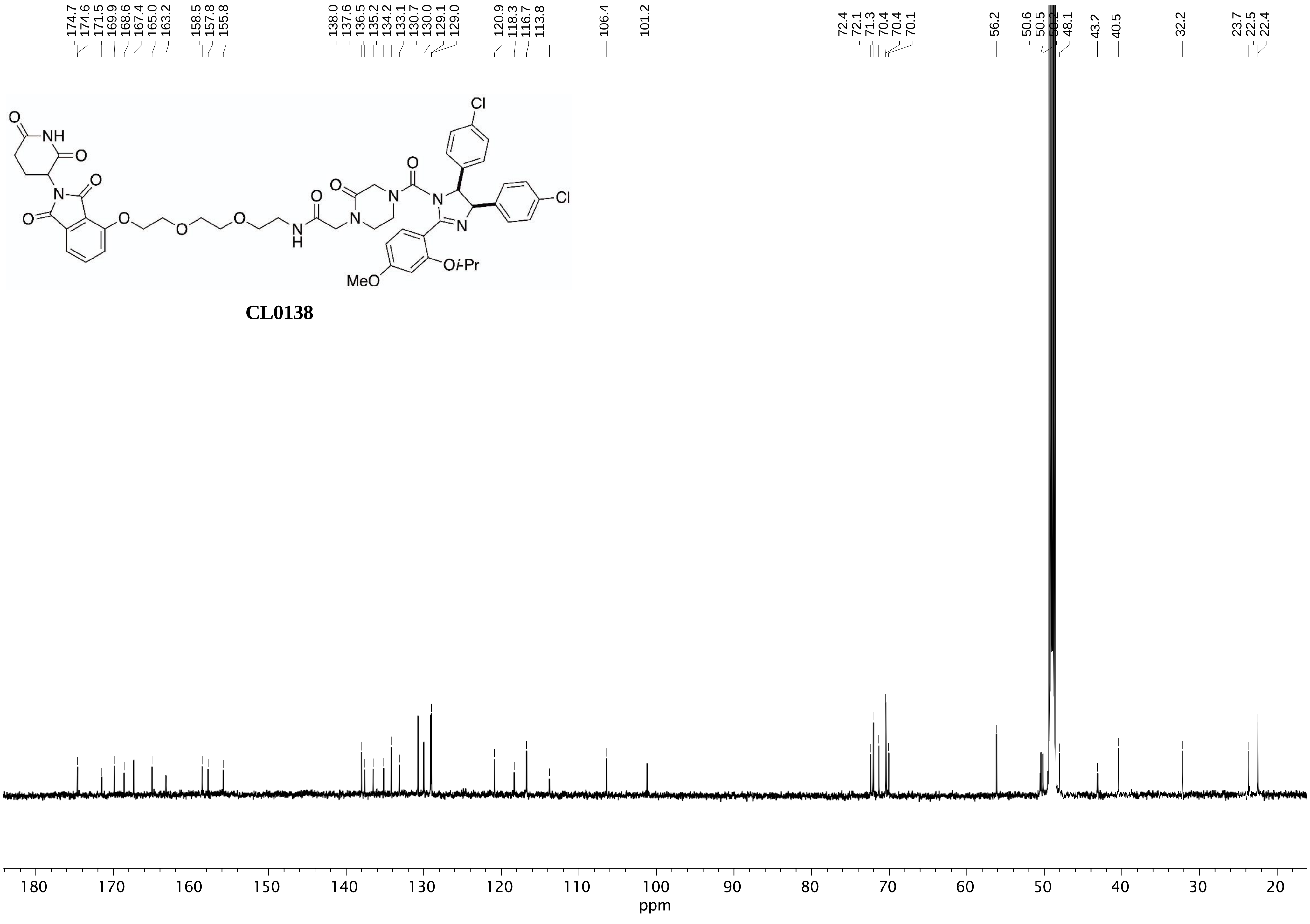

174.7
174.6
171.5
169.9
168.6
167.4
165.0
163.2
158.5
157.8
155.8
138.0
137.6
136.5
135.2
134.2
133.1
130.7
130.0
129.1
129.0
120.9
118.3
116.7
113.8
106.4
101.2
72.4
72.1
71.3
70.4
70.4
70.1
56.2
50.6
50.5
50.2
48.1
43.2
40.5
32.2
23.7
22.5
22.4
NH
O
N
Cl
MeO
O*i*-Pr
180 170 160 150 140 130 120 110 100 90 80 70 60 50 40 30 20
ppm

CL0138

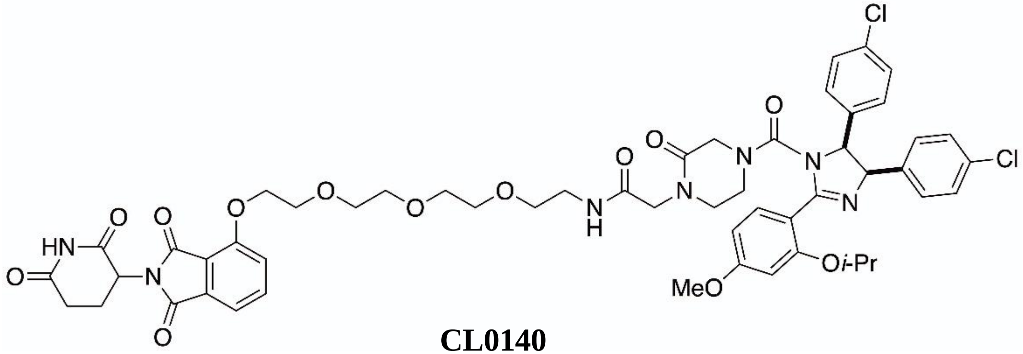
HN
O
N
O
O
O
O
O
N
H
O
O
N
N
O
N
N
Cl
Cl
MeO
O*i*-Pr

CL0140

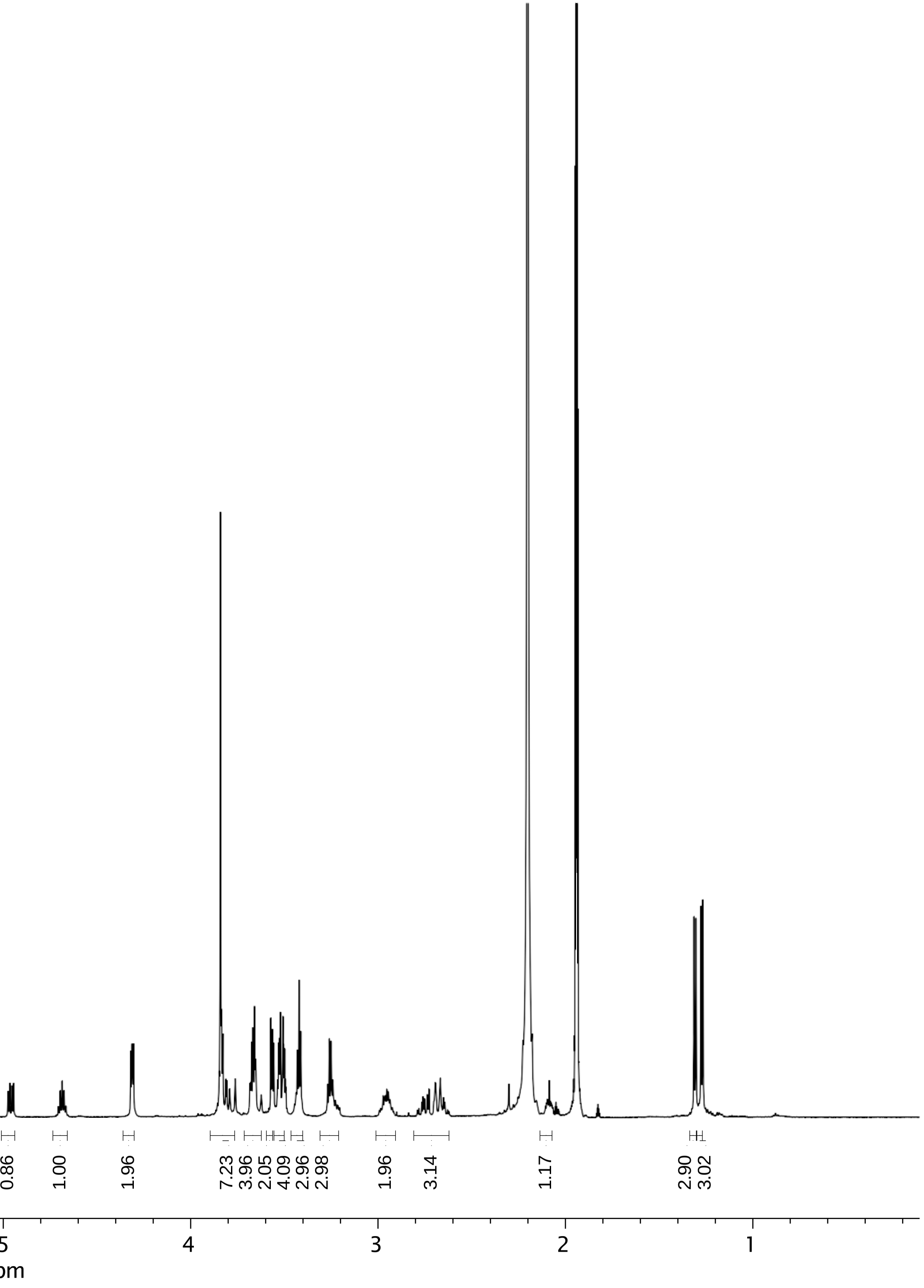
0.75
1.00
0.95
0.92
0.96
1.84
1.95
1.80
1.77
0.86
1.93
0.90
0.86
0.86
1.00
1.96
7.23
3.96
2.05
4.09
2.96
2.98
1.96
3.14
1.17
2.90
3.02
9
8
7
6
5
4
3
2
1
ppm

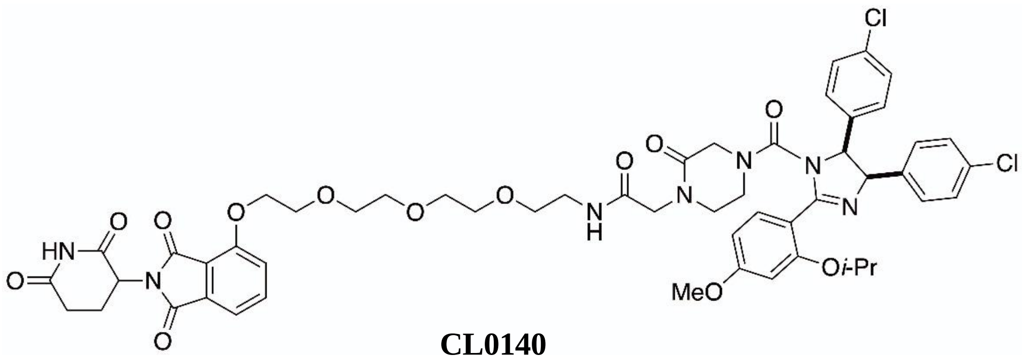

CL0140

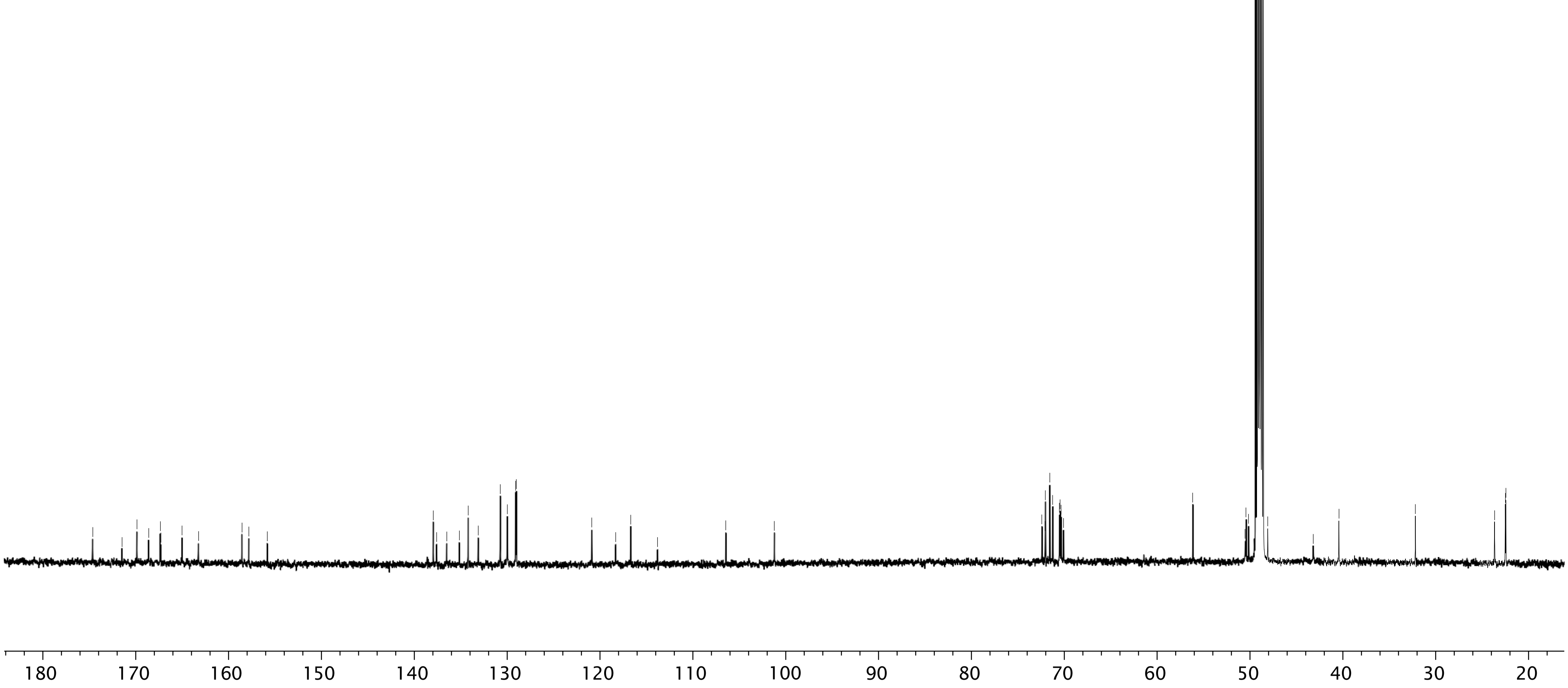

174.6
171.5
169.9
168.6
167.3
167.3
165.0
163.2
158.6
157.8
155.8
138.0
137.6
136.5
135.1
134.2
133.1
130.7
130.0
129.1
129.0
120.9
118.3
116.7
113.8
106.5
101.2
72.4
72.1
72.0
71.6
71.3
70.5
70.5
70.3
70.1
56.2
50.5
50.4
50.2
48.1
43.2
40.4
32.2
23.7
22.5
22.4
180
170
160
150
140
130
120
110
100
90
80
70
60
50
40
30
20
ppm

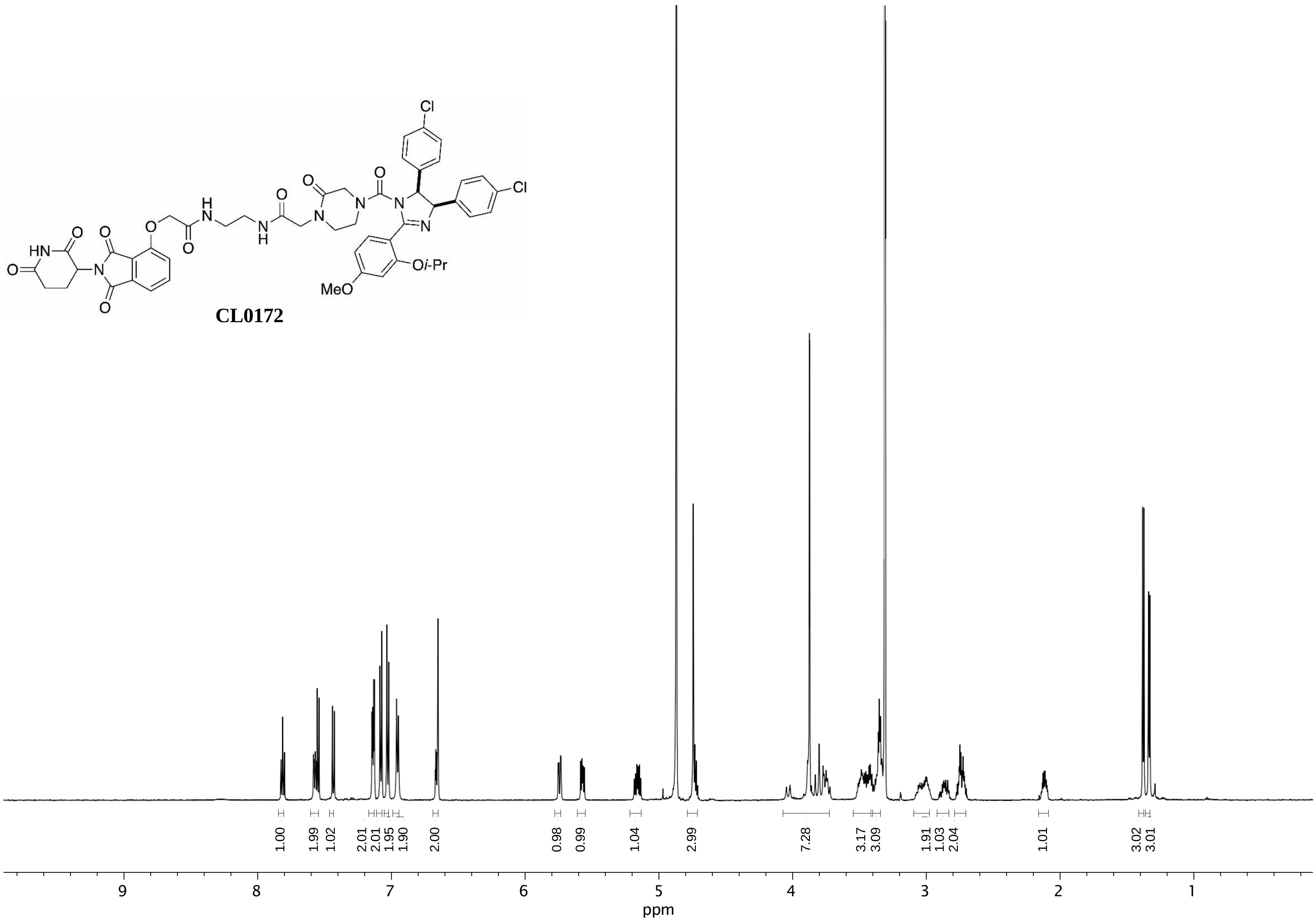
HN
O
O
O
N
O
O
O
N
H
N
H
O
N
O
N
O
N
N
Cl
Cl
Oi-Pr
MeO
CL0172
1.00
1.99
1.02
2.01
2.01
1.95
1.90
2.00
0.98
0.99
1.04
2.99
7.28
3.17
3.09
1.91
1.03
2.04
1.01
3.02
3.01
9
8
7
6
5
4
3
2
1
ppm

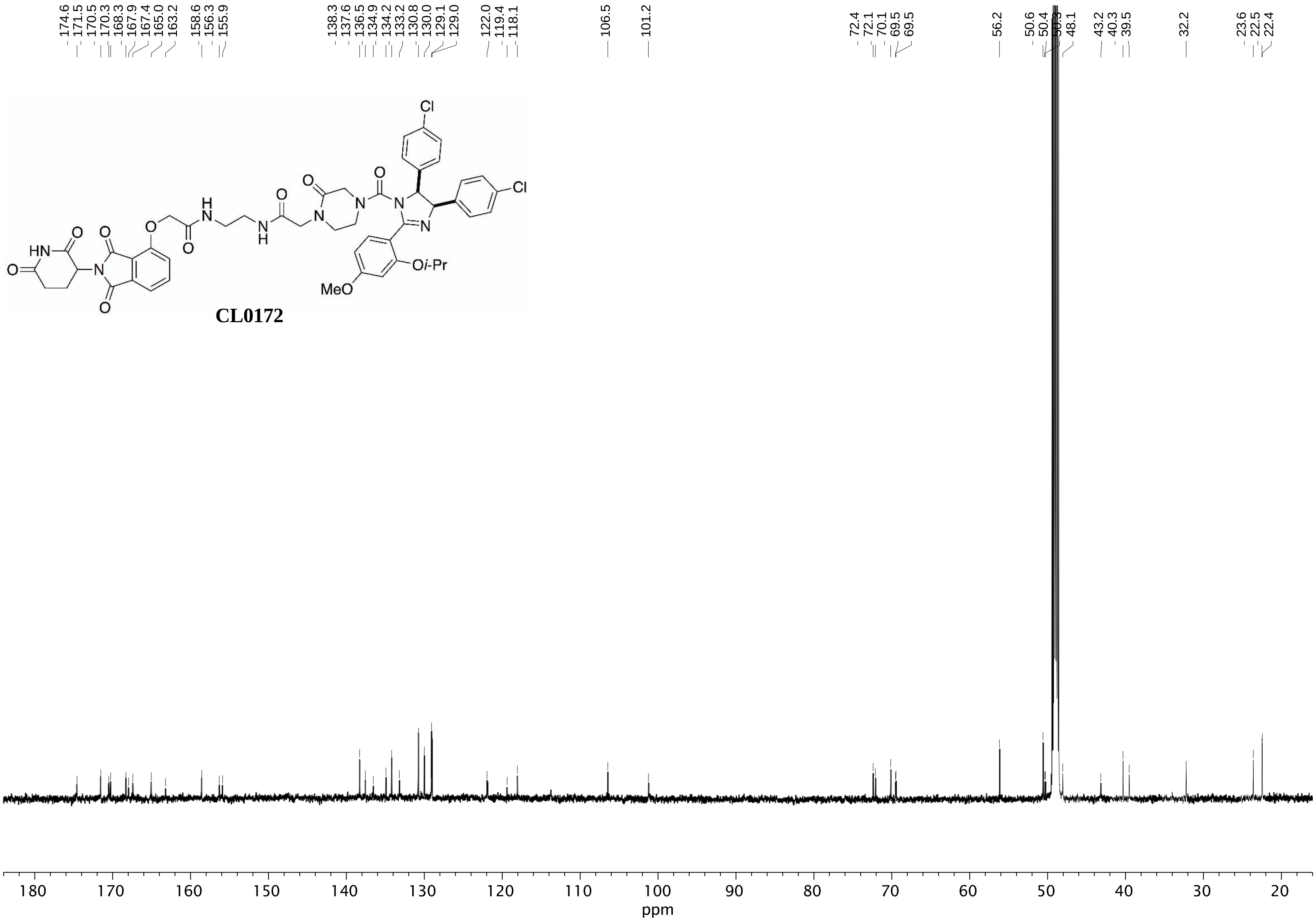
174.6
171.5
170.5
170.3
168.3
167.9
167.4
165.0
163.2
158.6
156.3
155.9
138.3
137.6
136.5
134.9
134.2
133.2
130.8
130.0
129.1
129.0
122.0
119.4
118.1
106.5
101.2
72.4
72.1
70.1
69.5
69.5
56.2
50.6
50.4
50.3
48.1
43.2
40.3
39.5
32.2
23.6
22.5
22.4
CL0172
180 170 160 150 140 130 120 110 100 90 80 70 60 50 40 30 20
ppm

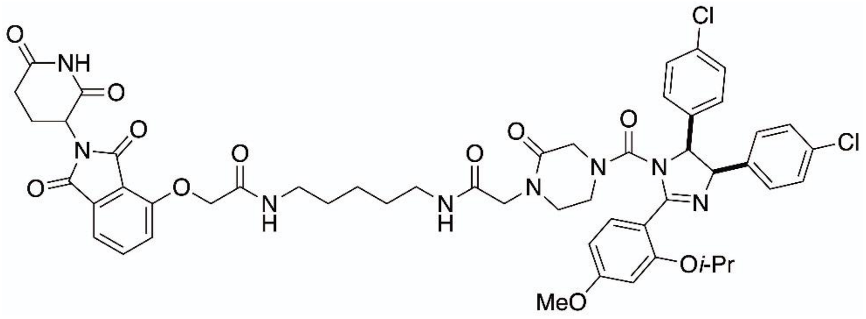

O
NH
O
N
O
O
O
O
N
H
N
H
O
O
N
N
O
N
N
Cl
Cl
Oi-Pr
MeO

CL0171

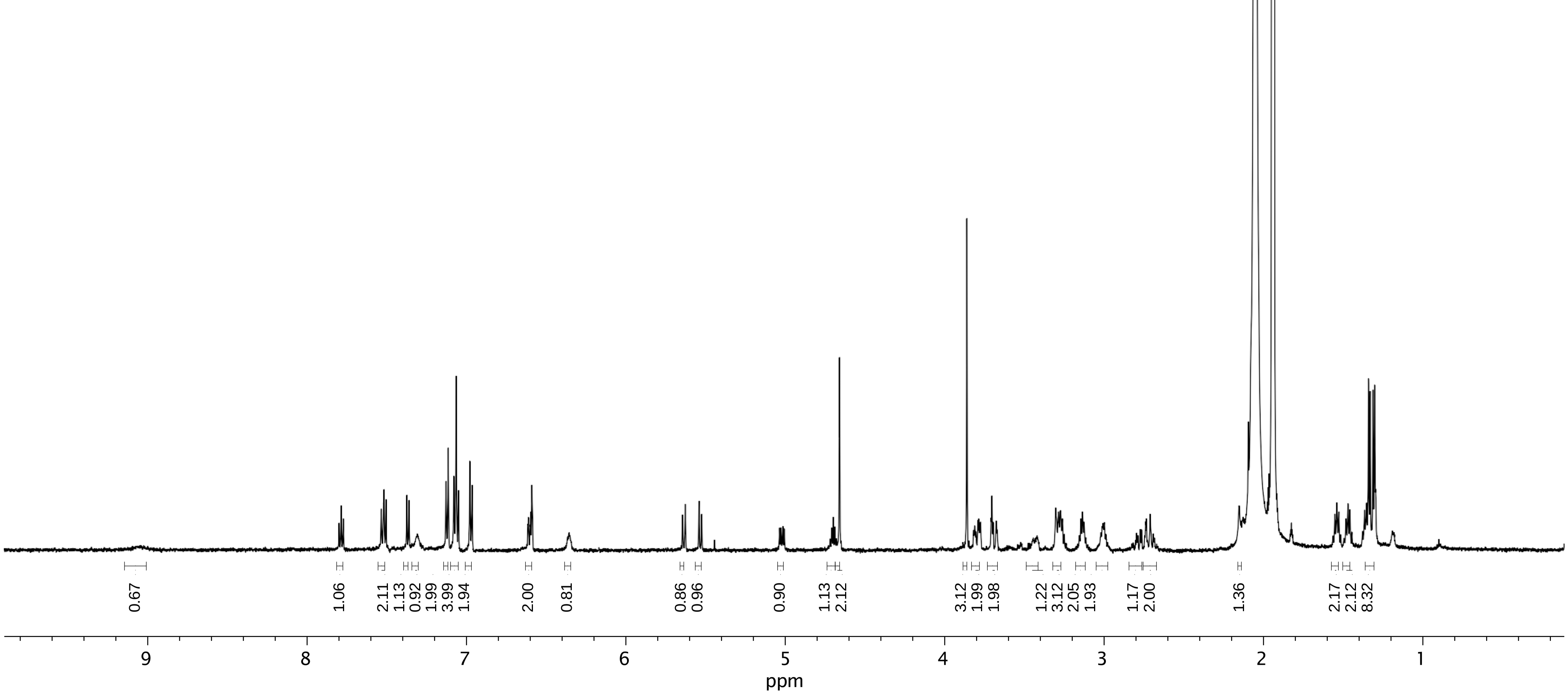

0.67
1.06
2.11
1.13
0.92
1.99
3.99
1.94
2.00
0.81
0.86
0.96
0.90
1.13
2.12
3.12
1.99
1.98
1.22
3.12
2.05
1.93
1.17
2.00
1.36
2.17
2.12
8.32
9
8
7
6
5
4
3
2
1
ppm

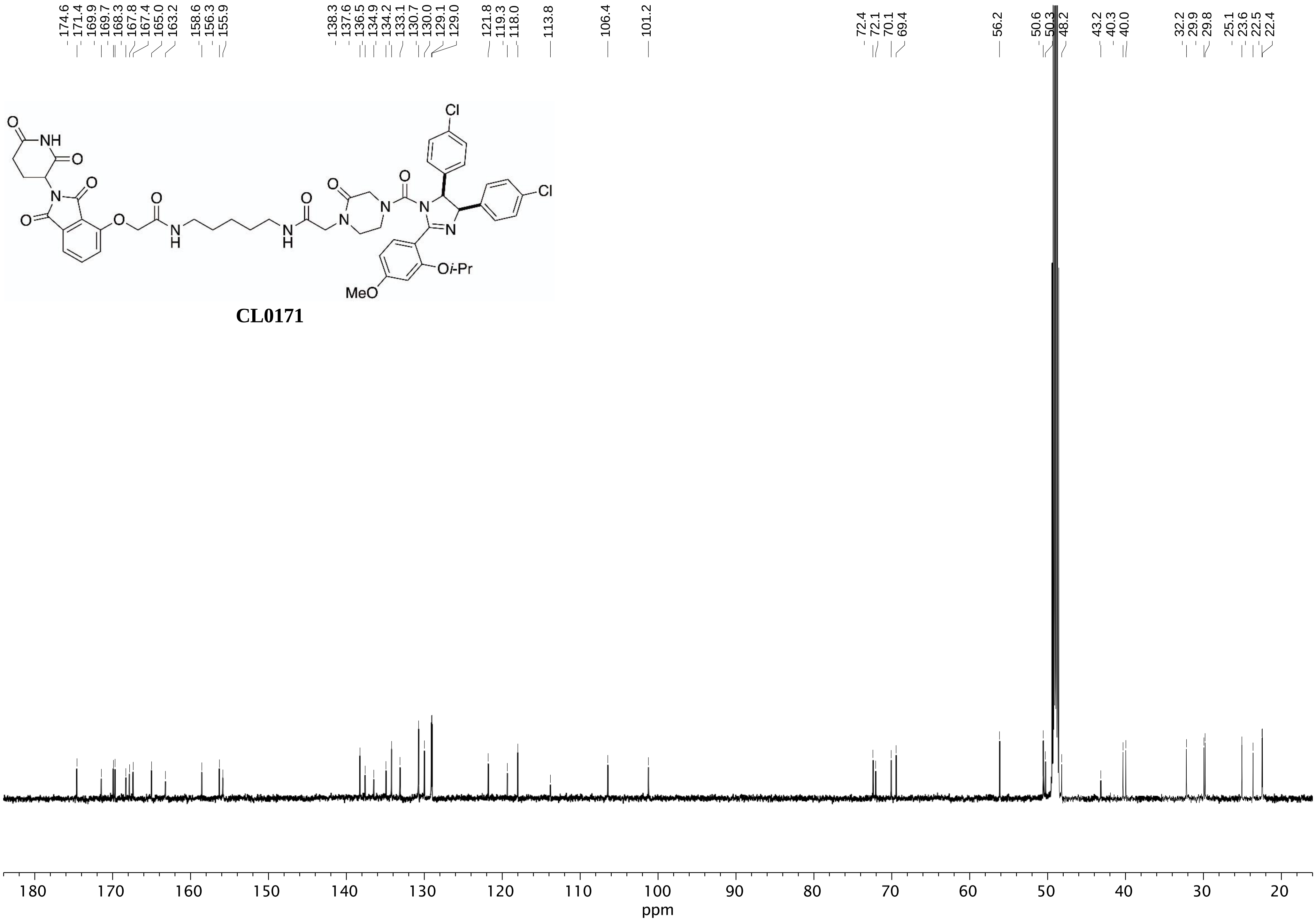
174.6
171.4
169.9
169.7
168.3
167.8
167.4
165.0
163.2
158.6
156.3
155.9
138.3
137.6
136.5
134.9
134.2
133.1
130.7
130.0
129.1
129.0
121.8
119.3
118.0
113.8
106.4
101.2
72.4
72.1
70.1
69.4
56.2
50.6
50.3
48.2
43.2
40.3
40.0
32.2
29.9
29.8
25.1
23.6
22.5
22.4
CL0171
180 170 160 150 140 130 120 110 100 90 80 70 60 50 40 30 20
ppm

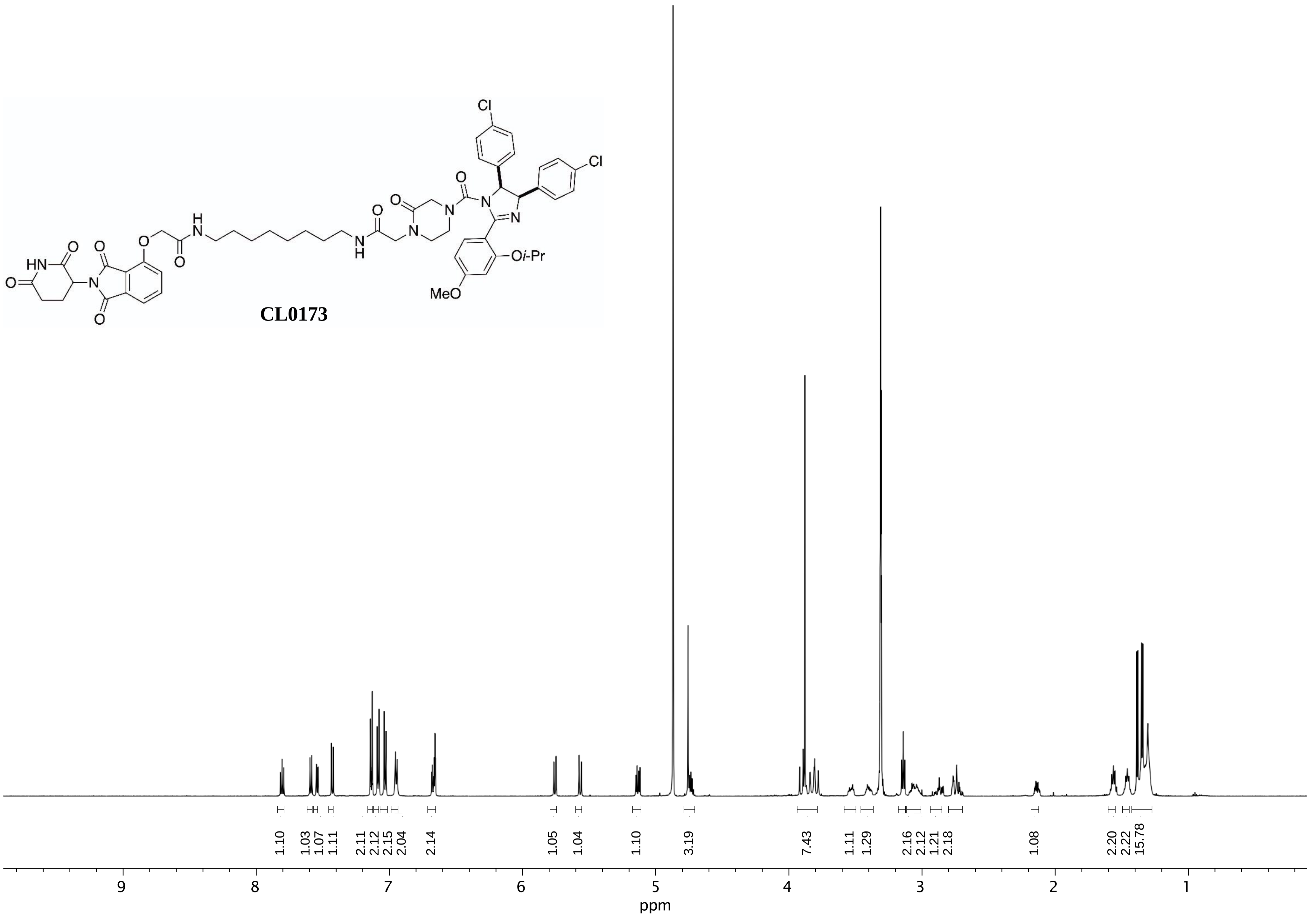

CL0173
HN
O
N
Cl
MeO
Oi-Pr
1.10
1.03
1.07
1.11
2.11
2.12
2.15
2.04
2.14
1.05
1.04
1.10
3.19
7.43
1.11
1.29
2.16
2.12
1.21
2.18
1.08
2.20
2.22
15.78
9
8
7
6
5
4
3
2
1
ppm

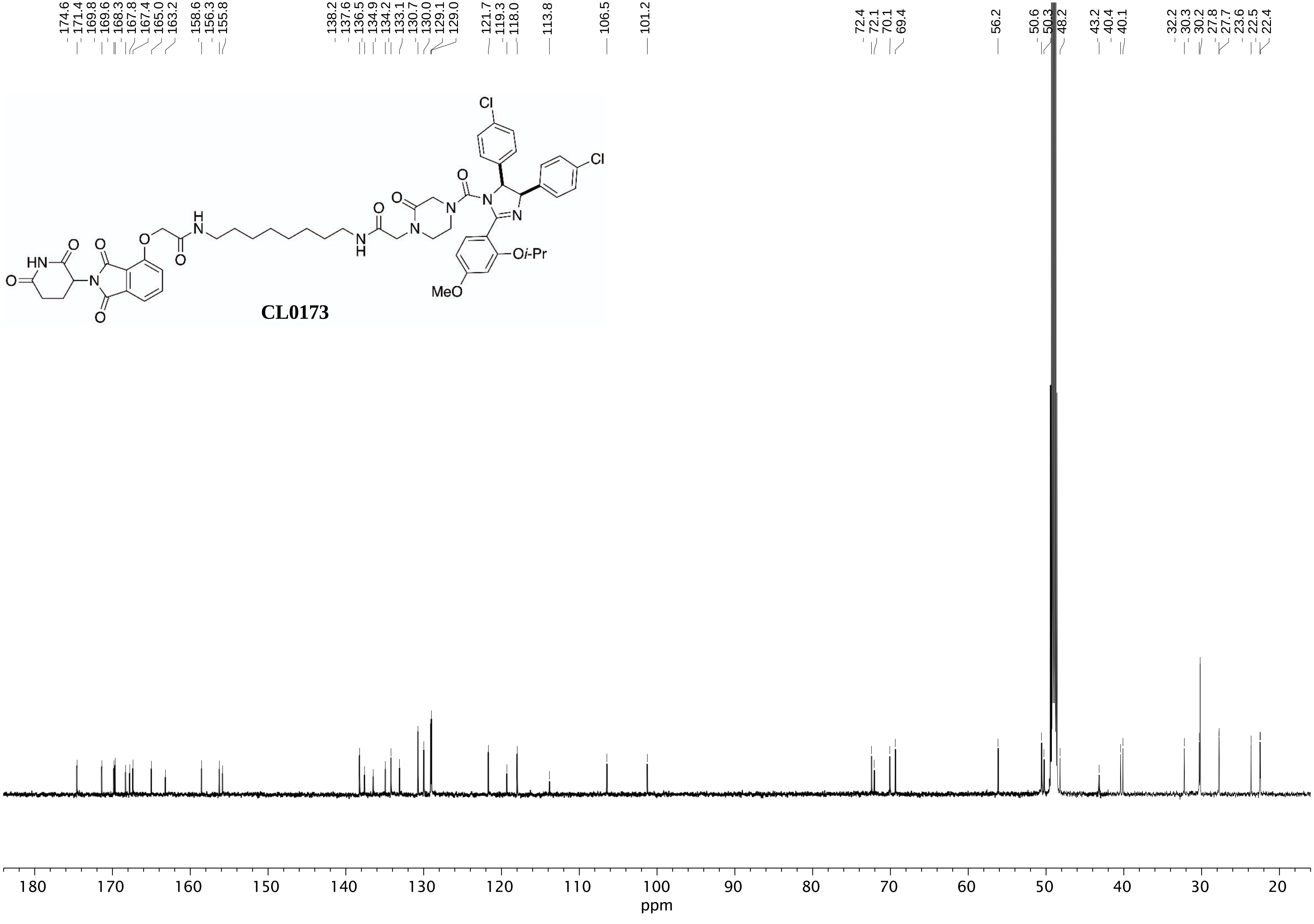
174.6
171.4
169.8
169.6
168.3
167.8
167.4
165.0
163.2
158.6
156.3
155.8
138.2
137.6
136.5
134.9
134.2
133.1
130.7
130.0
129.1
129.0
121.7
119.3
118.0
113.8
106.5
101.2
72.4
72.1
70.1
69.4
56.2
50.6
50.3
48.2
43.2
40.4
40.1
32.2
30.3
30.2
27.8
27.7
23.6
22.5
22.4
CL0173
HN
O
N
H
Cl
MeO
Oi-Pr
180
170
160
150
140
130
120
110
100
90
80
70
60
50
40
30
20
ppm

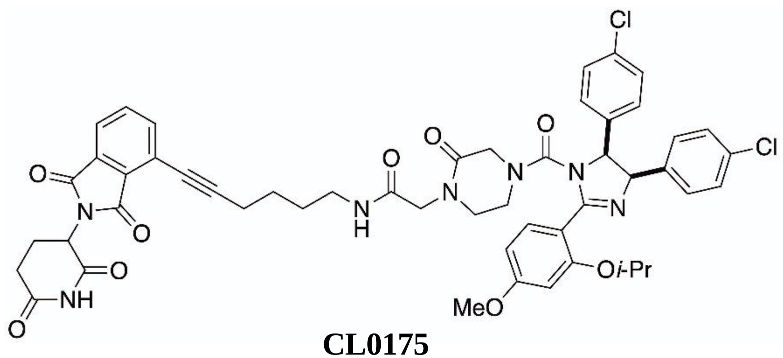
Cl
Cl
O
O
O
O
O
N
N
N
N
N
H
NH
O
O
Oi-Pr
MeO

CL0175

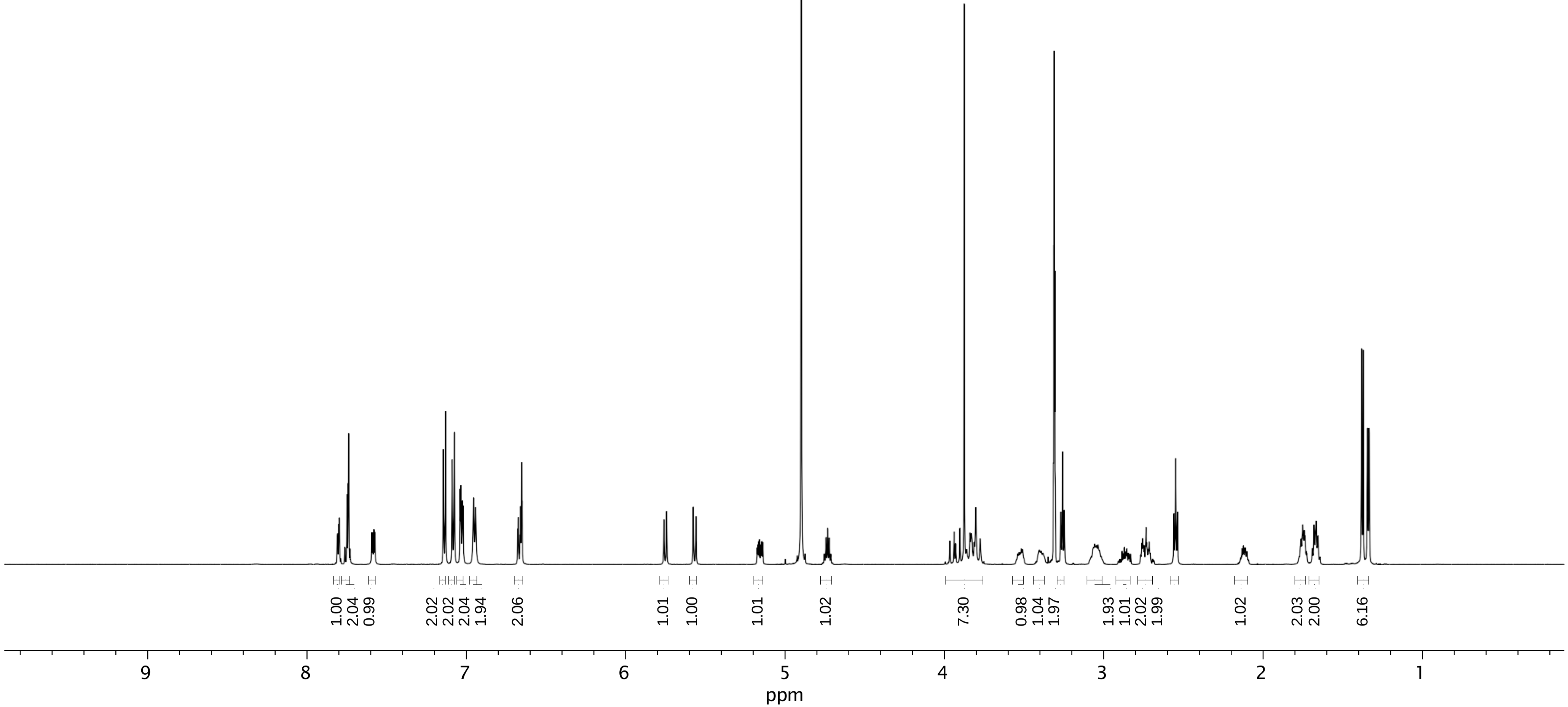
1.00
2.04
0.99
2.02
2.02
2.04
1.94
2.06
1.01
1.00
1.01
1.02
7.30
0.98
1.04
1.97
1.93
1.01
2.02
1.99
1.02
2.03
2.00
6.16
9
8
7
6
5
4
3
2
1
ppm

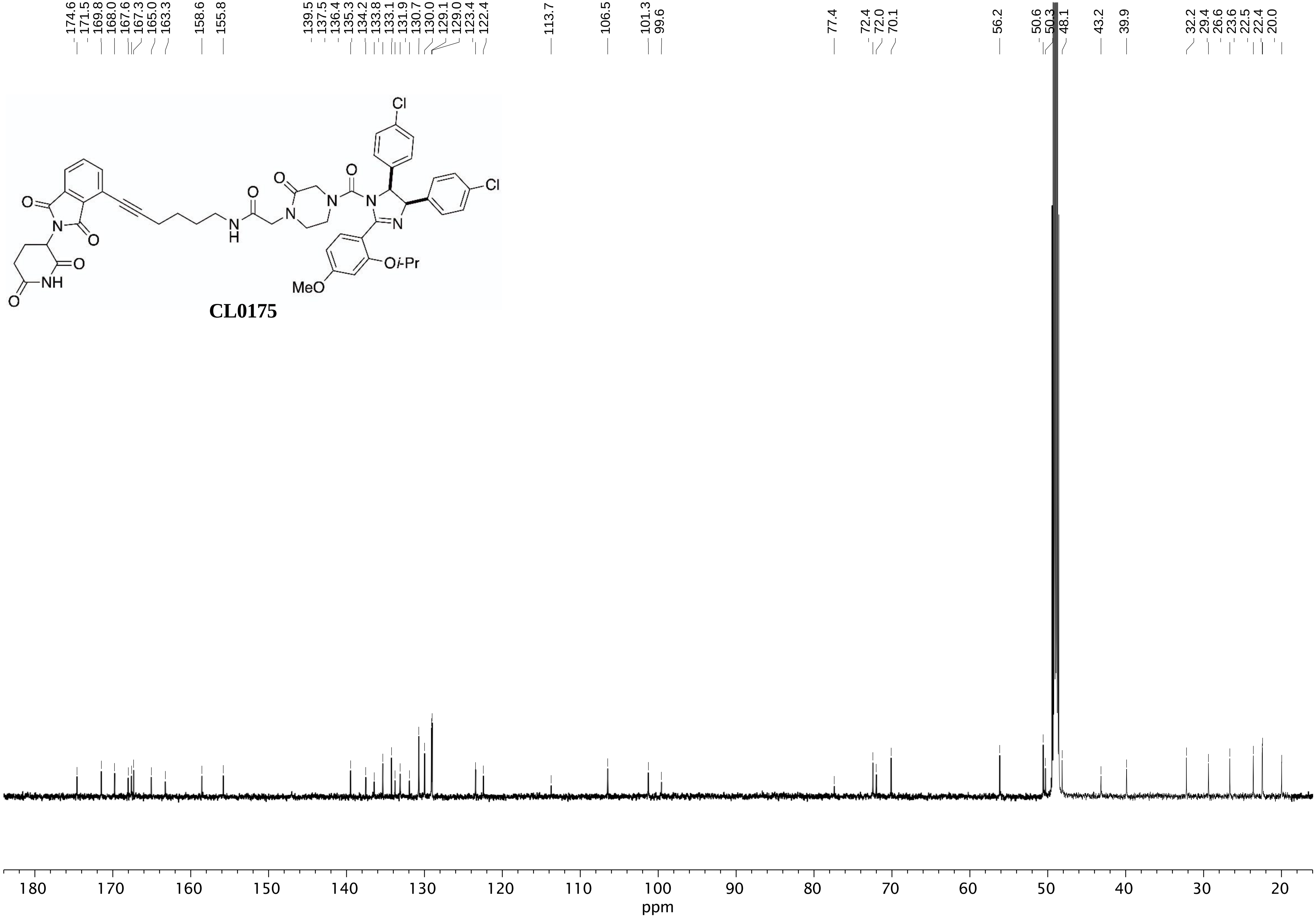
174.6
171.5
169.8
168.0
167.6
167.3
165.0
163.3
158.6
155.8
139.5
137.5
136.4
135.3
134.2
133.8
133.1
131.9
130.7
130.0
129.1
129.0
123.4
122.4
113.7
106.5
101.3
99.6
77.4
72.4
72.0
70.1
56.2
50.6
50.3
48.1
43.2
39.9
32.2
29.4
26.6
23.6
22.5
22.4
20.0
CL0175
180 170 160 150 140 130 120 110 100 90 80 70 60 50 40 30 20
ppm

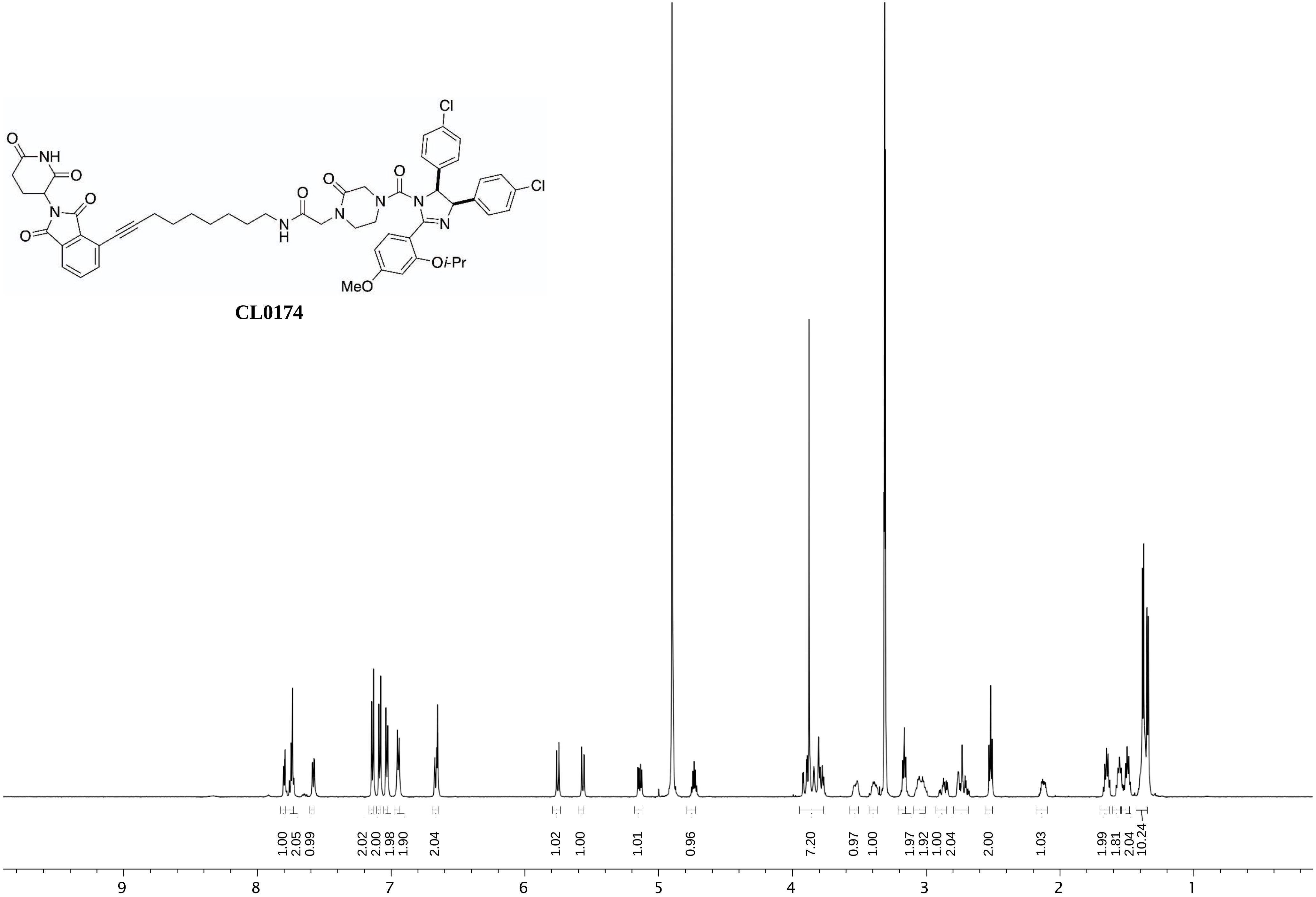
NH
O
N
Cl
MeO
Oi-Pr
CL0174
1.00
2.05
0.99
2.02
2.00
1.98
1.90
2.04
1.02
1.00
1.01
0.96
7.20
0.97
1.00
1.97
1.92
1.00
2.04
2.00
1.03
1.99
1.81
2.04
10.24
9
8
7
6
5
4
3
2
1
ppm

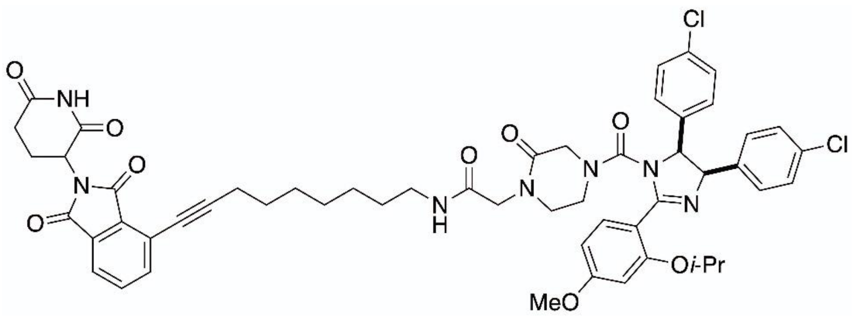

CL0174

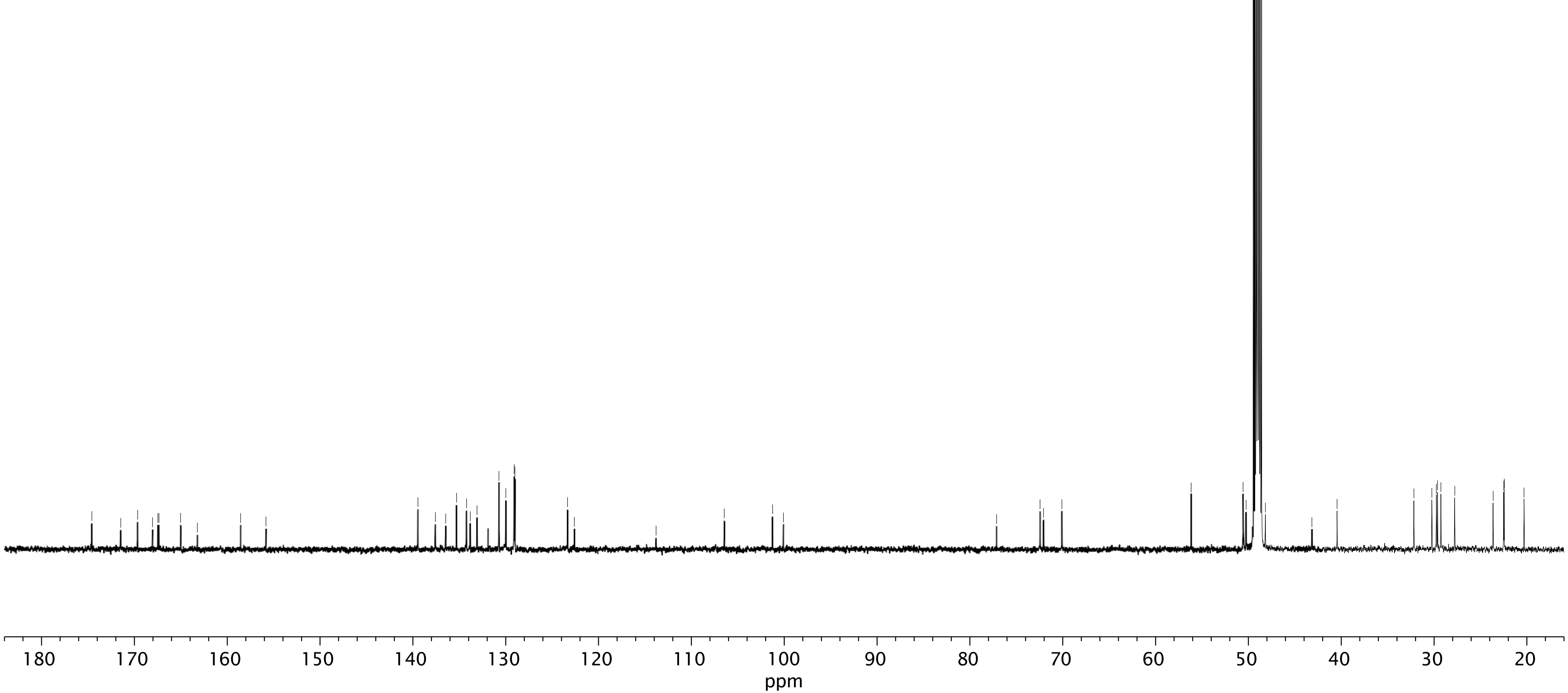
174.6
171.5
169.6
168.1
167.5
167.4
165.0
163.2
158.6
155.8
139.5
137.6
136.5
135.3
134.2
133.8
133.1
130.7
130.0
129.1
129.0
123.3
122.6
113.8
106.5
101.3
100.1
77.1
72.4
72.1
70.1
56.2
50.6
50.2
48.2
43.2
40.4
32.2
30.2
29.8
29.6
29.3
27.8
23.6
22.5
22.4
20.3
180
170
160
150
140
130
120
110
100
90
80
70
60
50
40
30
20
ppm

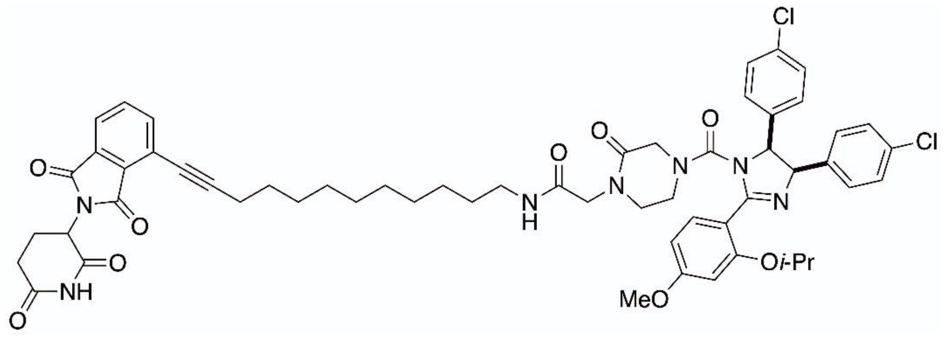

CL0176

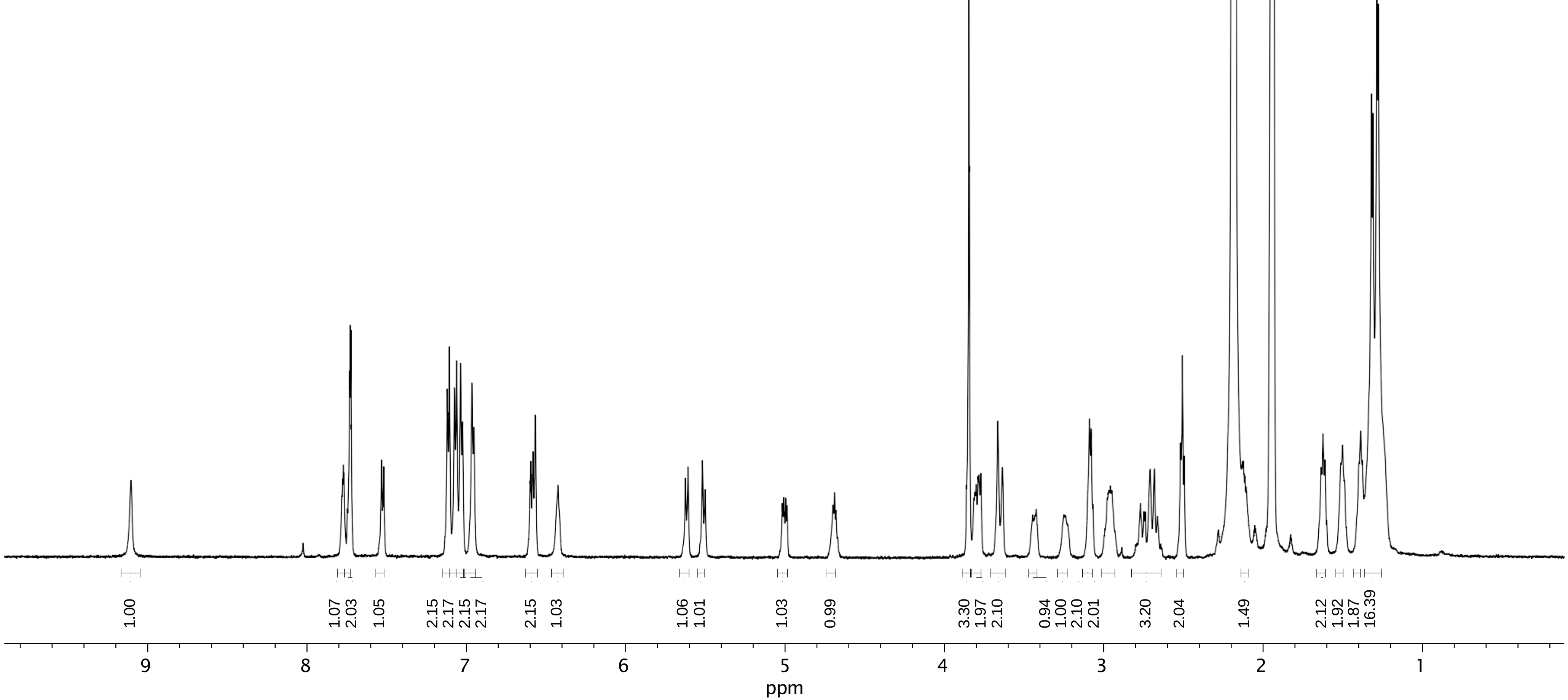
1.00
1.07
2.03
1.05
2.15
2.17
2.15
2.17
2.15
1.03
1.06
1.01
1.03
0.99
3.30
1.97
2.10
0.94
1.00
2.10
2.01
3.20
2.04
1.49
2.12
1.92
1.87
16.39
9
8
7
6
5
4
3
2
1
ppm

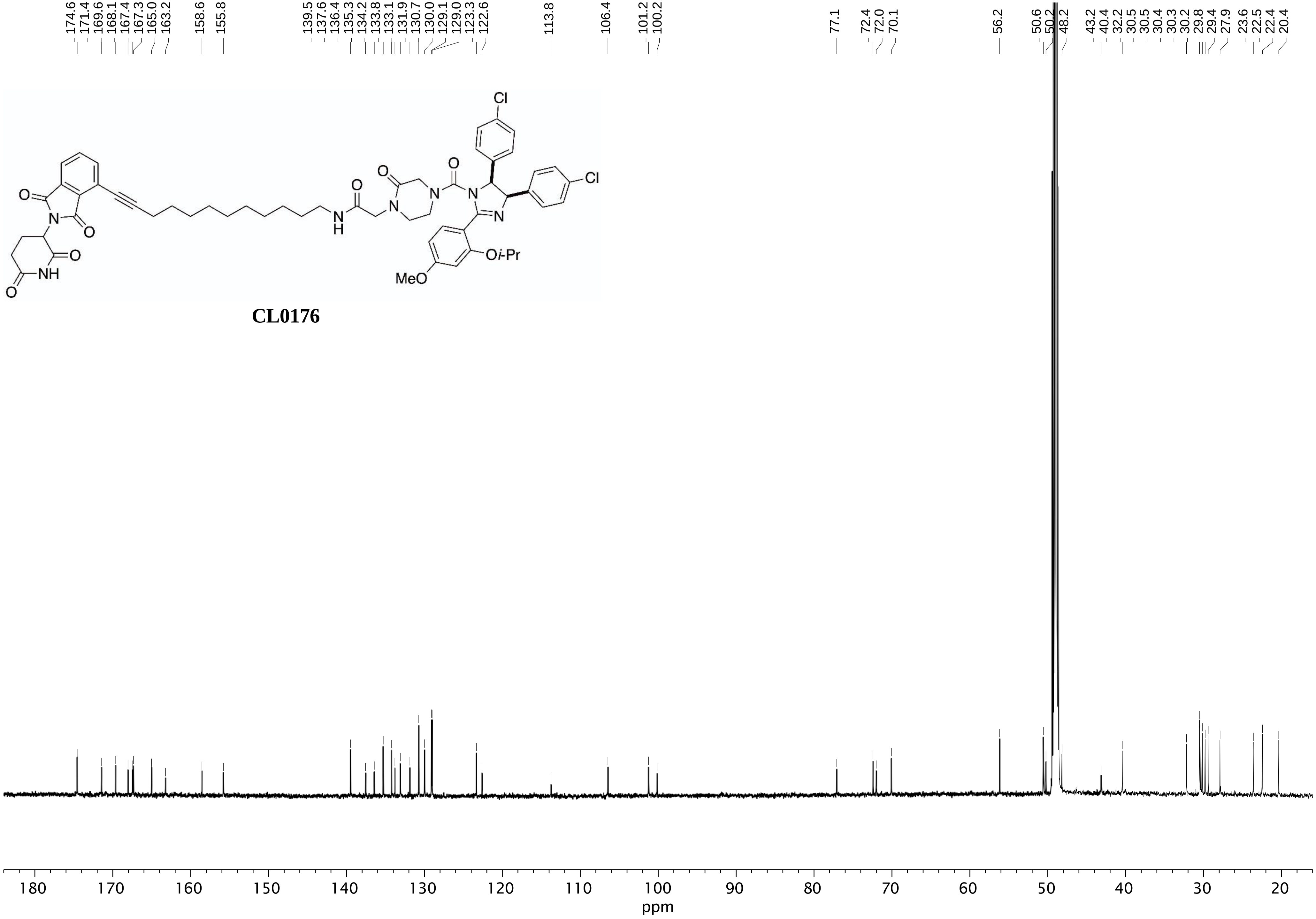

174.6
171.4
169.6
168.1
167.4
167.3
165.0
163.2
158.6
155.8
139.5
137.6
136.4
135.3
134.2
133.8
133.1
131.9
130.7
130.0
129.1
129.0
123.3
122.6
113.8
106.4
101.2
100.2
77.1
72.4
72.0
70.1
56.2
50.6
50.2
48.2
43.2
40.4
32.2
30.5
30.5
30.4
30.3
30.2
29.8
29.4
27.9
23.6
22.5
22.4
20.4
Cl
Cl
O
N
NH
Oi-Pr
MeO
CL0176
180
170
160
150
140
130
120
110
100
90
80
70
60
50
40
30
20
ppm

Supporting Information 2

Title: Development of MDM2-targeting PROTAC for advancing bone regeneration

Sol Jeong[1†], Jae-Kook Cha[2,3†], Wasim Ahmed[4†], Jaewan Kim[4], Minsup Kim[5], Kyung Tae Hong[6], Wonji Choi[4], Sunjoo Choi[4], Tae Hyeon Yoo[4], Hyun-Ju An[7], Seung Chan An[7], Jaemin Lee[7], Jimin Choi[2], Sun-Young Kim[8], Jun-Seok Lee[6], Soonchul Lee[7]*, Junwon Choi[4,9]*, Jin Man Kim[1,10,11]*

This file includes:

Figs. S1 to S9

Tables S1 to S3

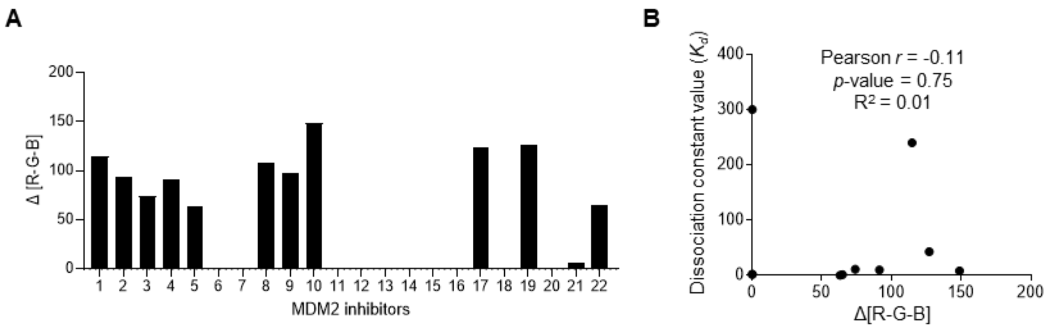

Figure S1. Quantification of osteogenic potency of MDM2 inhibitors and its correlation with binding affinity **(A)** Quantification of ARS staining result with MDM2 inhibitors treated human bone marrow-derived stem cells (hBMSCs). **(B)** Pearson correlation analysis between dissociation constant (K_d) values and Quantified ARS Staining; Dissociation constant lists of compounds is given Table S3.

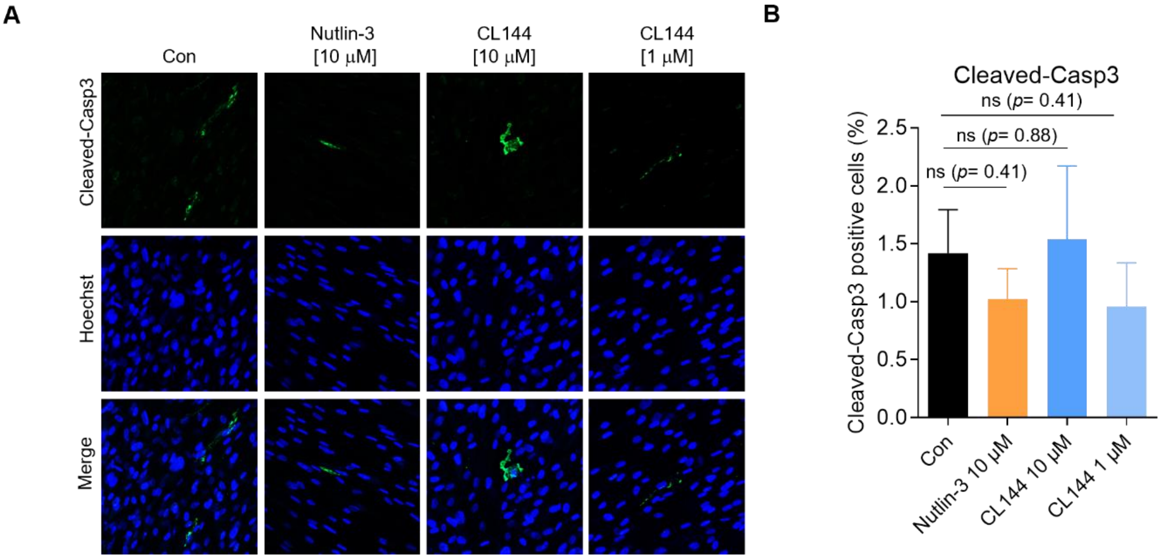

Figure S2. Apoptosis test of MDM2-targeting compounds.
(**A**) Immunofluorescence images of cleaved-caspase-3 in MDM2 inhibitor (Nutlin-3) and our MDM2-PROTAC (CL144) treated BMSCs. **(B)** Proportion of the cleaved-caspase-3 positive cells; Student's t-test: ns = non-significant; mean with SEM; n=5.

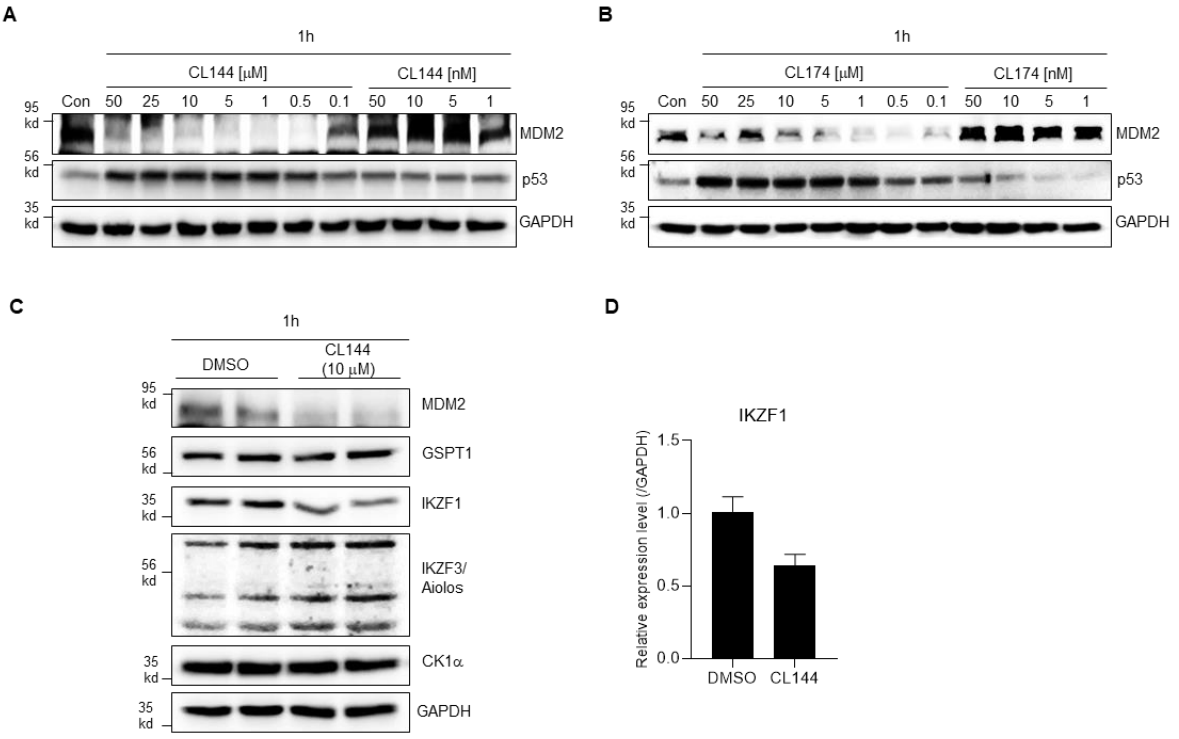

Figure S3. Evaluating the degradation window and Off-target profiles of developed MDM2 targeting PROTACs **(A, B)** Immunoblot image of MDM2 and p53 protein in the concentration range of 1 nM-50 mM for CL144 and CL174 in hBMSCs; GAPDH was used as loading control. **(C)** Neo-substrate immunoblot assay under the condition of 10 μM CL144 (MDM2-PROTAC treated human bone marrow-derived stem cells (hBMSCs). **(D)** Quantitative analysis of IKZF1 from the immunoblot in (C).

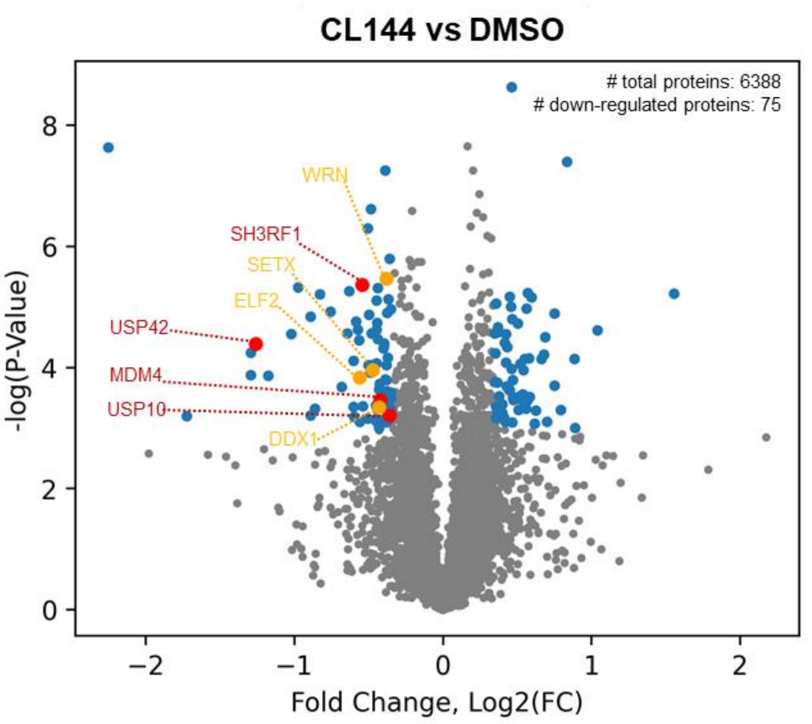

Figure S4. Proteomics analysis of MDM2-PROTAC (CL144) in hBMSCs.
Volcano plot showing protein abundance (Log2(Fold Change)) as a function of *p*-values [−log10(*p*-value)]. Blue: differentially expressed proteins; Red: MDM2–p53 network and ubiquitin processing-related proteins; Yellow: transcription-related proteins.

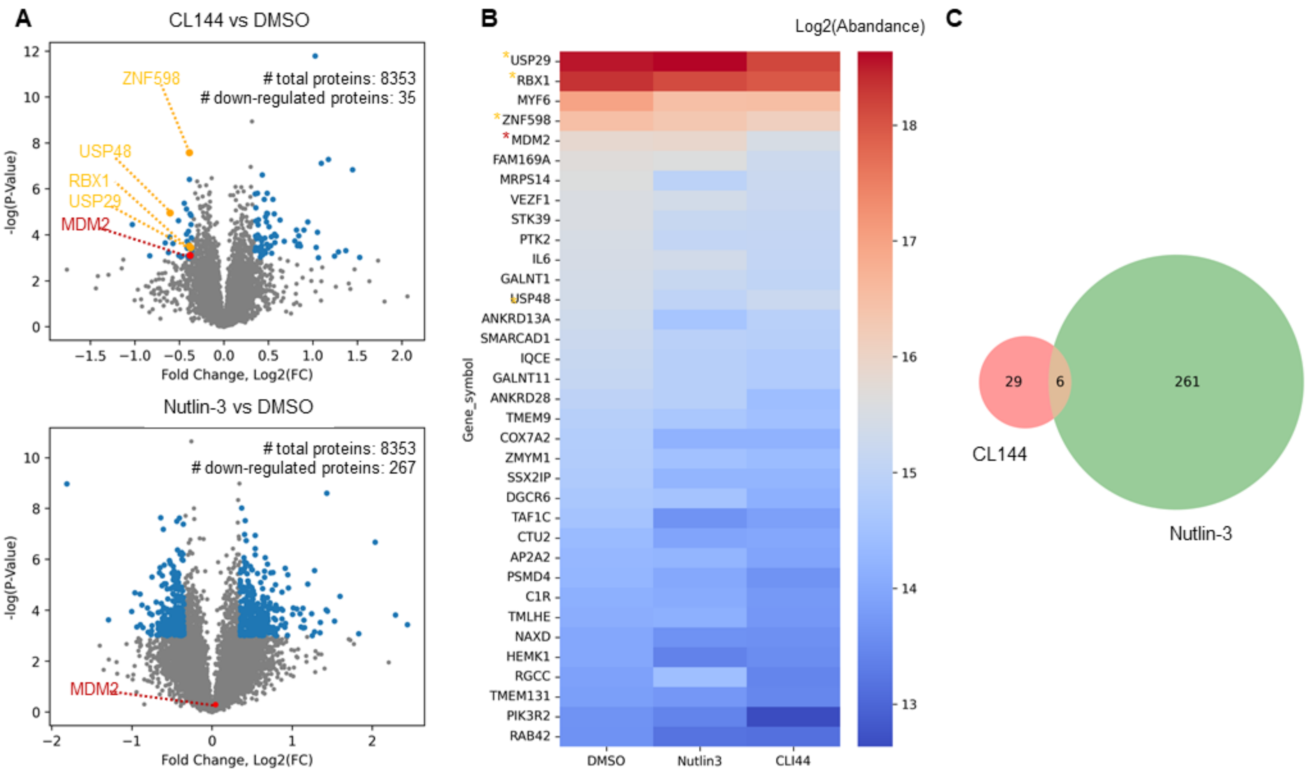

Figure S5. Proteomics analysis of MDM2-PROTAC (CL144) and Nutlin-3 in MDM2-overexpressing HeLa Cells.

(**A**) Volcano plots showing protein abundance changes [Log2(Fold Change)] as a function of *p*-values [−log10(*p*-value)] of the proteomic analysis of cells treated with CL144 or Nutlin-3. Red: MDM2−p53 network and ubiquitin processing-related proteins; Yellow: transcription-related proteins. (**B**) Heat map showing protein abundance changes in each group, DMSO, Nutlin-3, and CL144. Red indicates downregulated proteins. (**C**) Venn diagram illustrating the overlap of downregulated proteins between two comparison groups: CL144 vs. DMSO and Nutlin-3 vs. DMSO.

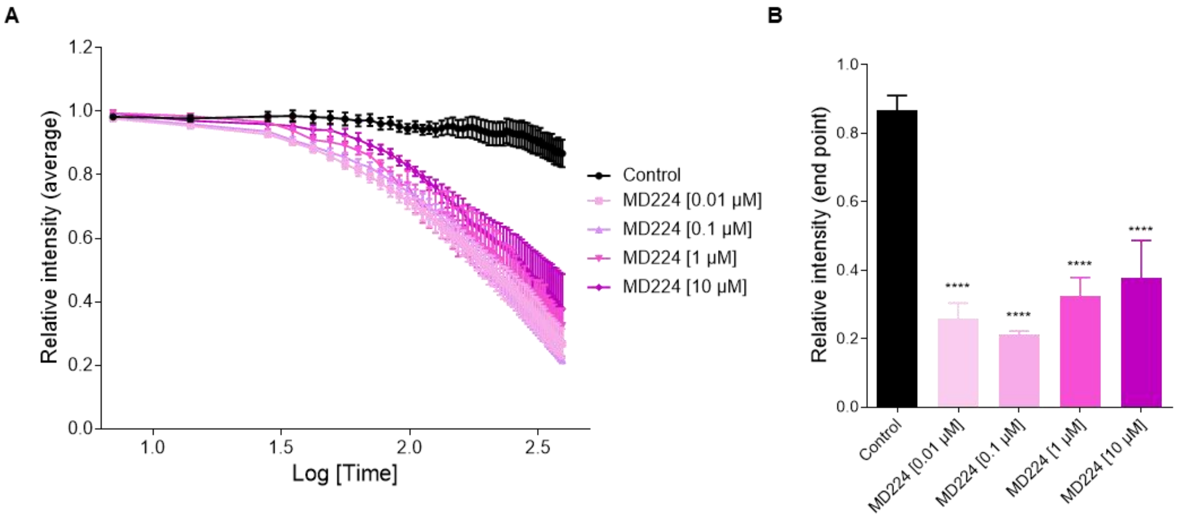

Figure S6. Validation of EGFP-MDM2 degradation kinetics and maximum efficiency of MD224 at various concentration

(A) Live imaging quantification of cells expressing EGFP-MDM2 after treatment with previously characterized MDM2-PROTACs (MD224). Quantification values were calculated from the EGFP signals; mean with SEM; n=3 to 8. **(B)** Evaluation of the maximum degradation efficiency of MD224 at 0.01 to 10 µM concentrations; ANOVA Bonferroni test: ****p<0.0001; mean with SEM; n=3 to 8

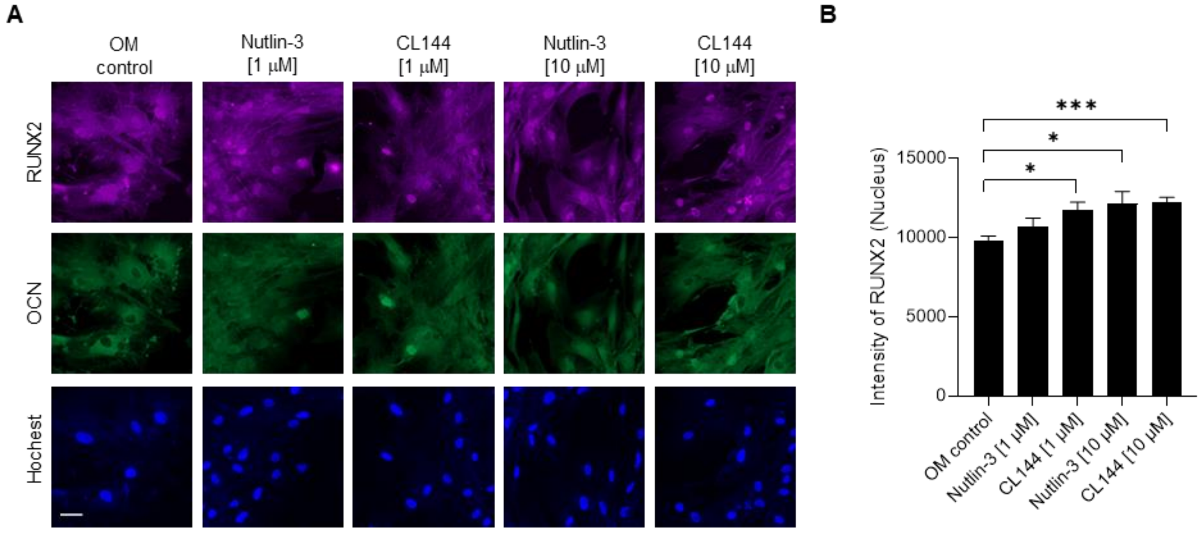

Figure S7. Immunostaining of key factors inducing osteogenic differentiation: RUNX2 and Osteocalcin (OCN) (A) Immunofluorescence images of RUNX2 and Osteocalcin in MDM2 inhibitor (Nutlin-3) and MDM2-PROTAC (CL144) treated human bone marrow-derived stem cells (hBMSCs). Scale bar = 50 µm; mean with SEM; n=5 to 9. **(B)** Quantitative analysis of RUNX2 from the images in (A); Student's t-test *p<0.05, ***p<0.005; mean with SEM; n=5 to 9

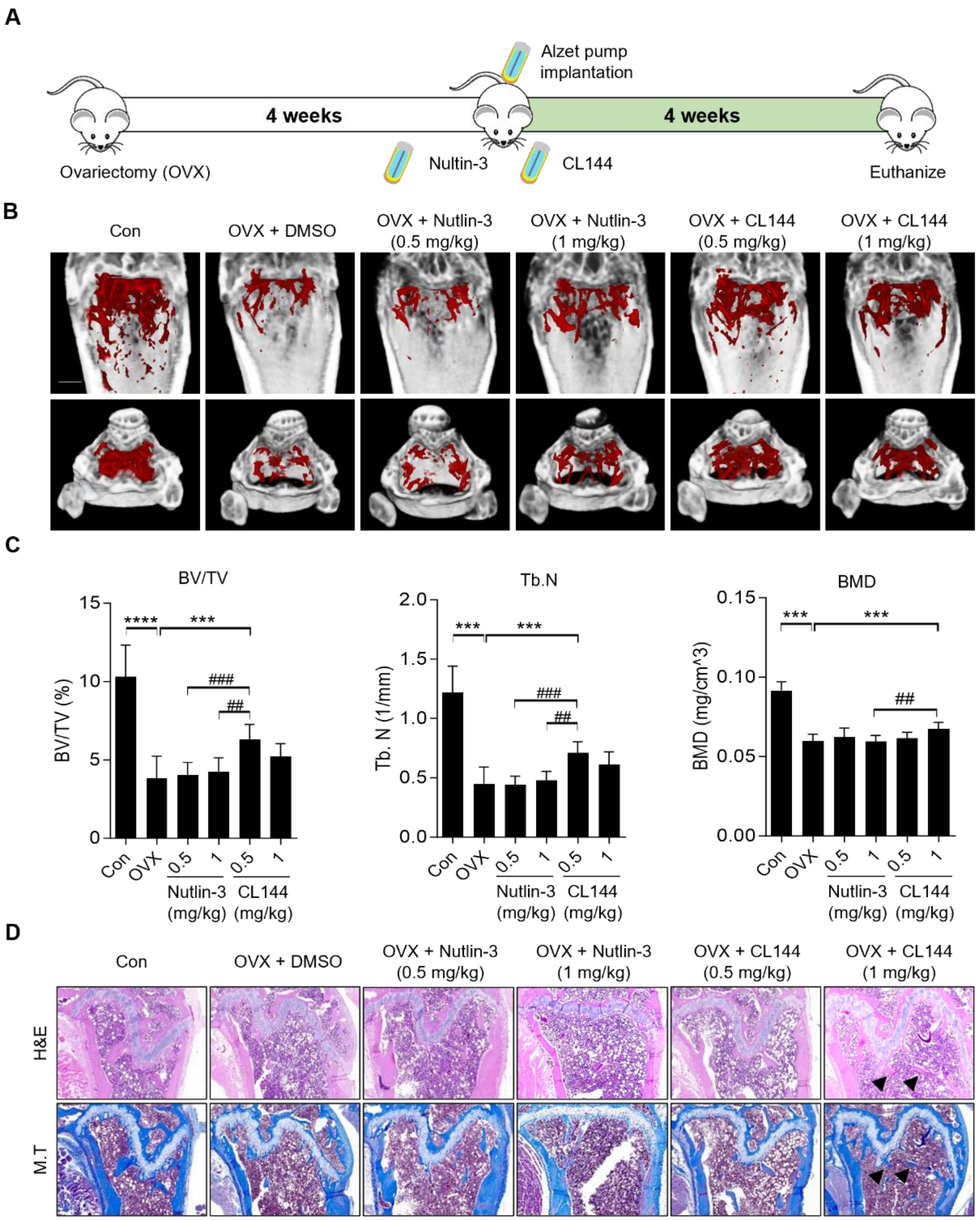

Figure S8. Comparative evaluation of MDM2-PROTAC and Nutlin-3 in bone regeneration.
(A) Schematic diagram of OVX-induced osteoporosis model; 4 weeks administration of CL144 or Nutlin-3 using implanted Alzet pumps in mice induced with osteoporotic conditions through ovariectomy (OVX) surgery. **(B)** The micro-CT 3D analysis of femur tissues in an OVX-induced osteoporosis model, following administration of Nultin-3 or CL144 at various concentrations; white structure: cortical bone, red structure: trabecular bone, Scale bar = 200 µm. **(C)** The bone formation parameters analysis of bone volume/tissue volume (BV/TV), trabecular number (Tb.N), and bone mineral density (BMD) in OVX model; ANOVA Bonferroni test: ***p*<0.005, ****p*<0.001, *****p*<0.0001, ##*p*<0.01, ###*p*<0.001; mean with SEM; n=8 to 10. **(D)** Histological analysis of the model femur tissue using H&E and Masson's trichrome staining. Black arrow heads indicate newly formed bone.

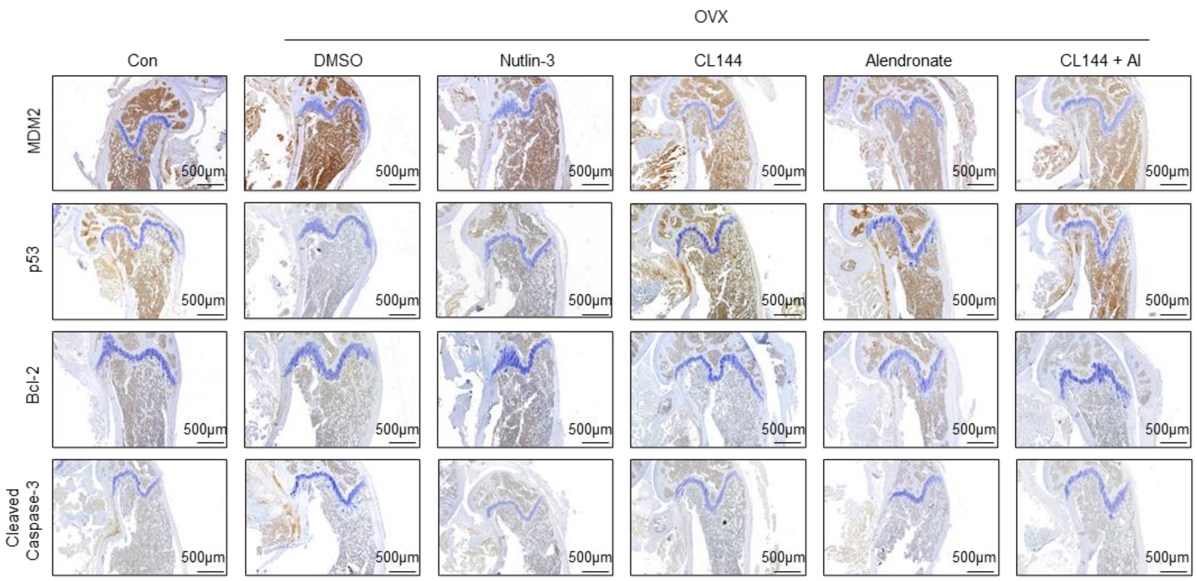

Figure S9. Assessment of bone marrow apoptosis in osteoporotic mice model treated with MDM2-PROTAC.

Immunohistochemistry analysis of the model femur tissue in preclinical models. Bcl-2 is used anti-apoptotic marker and Cleaved-caspase3 is used pro-apoptotic marker; Scale bar = 500 μm

Table S1. The list of MDM2 inhibitors used in this study

No.	Company	Molecule name	Cat. No.	Solvent	Final conc. (μM)
1	Sigma	Nutlin-3	N6287	DMSO	10
2	MCE	Nutlin-3a	HY-10029	DMSO	10
3	MCE	RG7112 (RO5045337)	HY-15676	DMSO	10
4	MCE	Idasanutlin (RG7388)	HY-15676	DMSO	10
5	MCE	Navtemadlin (AMG-232)	HY-12296	DMSO	10
6	MCE	Alrizomadlin (APG-115)	HY-101518	DMSO	10
7	MCE	NVP-CGM097 (CGM097)	HY-15954	DMSO	10
8	MCE	Siremadlin (NVP-HDM201)	HY-18658	DMSO	10
9	MCE	Milademetan (DS-3032)	HY-101266	DMSO	10
10	MCE	MI-773	HY-17493	DMSO	10
11	Adooq	YH239-EE	A14118	DMSO	10
12	MCE	NSC 66811	HY-14967	DMSO	10
13	MCE	Lithocholic acid	HY-B0172	DMSO	10
14	Sigma	α-Mangostin	M3824	MeOH	10
15	Sigma	*R*,*S*-Gambogic acid	PHL80455	DMSO	10
16	MCE	SJ-172550	HY-16664	DMSO	10
17	MCE	Serdemetan (JNJ-26854165)	HY-12025	DMSO	10
18	Tocris	RITA	2443	DMSO	10
19	MCE	SP-141	HY-110182	DMSO	10
20	MedKoo	Cytarabine hydrochloride (MK-8242)	100200	DMSO	10
21	Calbiochem	RO-5963	444153	DMSO	10
22	MCE	MI-1061	HY-125858	DMSO	10

Table S2. The list of antibodies and primers used in this study

Antibodies	Source	Identifier
anti -MDM2	Santa Cruz	sc-965
anti -p53	Santa Cruz	sc-126
anti -GAPDH	Santa Cruz	sc-47724
anti-HA-tag	CST	#3724
Mouse anti-Rabbit IgG-HRP	Santa Cruz	sc-2357
Goat anti-mouse IgG(H+L)-HRP	GenDEPOT	SA001-500
**qRT-PCR Primer sequences**	**Gene name**	**forward/reverse**
ACGGATTTGGCCGTATT	GAPDH	forward
TTGACTGTGCCGTGGAATTTG	GAPDH	reverse
AACCCTTAATTTGCACTGGGTCA	RUNX2	forward
CAAATTCCAGCAATGTTTGTGCTAC	RUNX2	reverse
CCCAGGCGCTACCTGTATCAA	OCN	forward
GGTCAGCCAACTCGTCACAGTC	OCN	reverse
ACACATATGATGGCCGAGGTGA	OPN	forward
TGTGAGGTGATGTCCTCGTCTGTAG	OPN	reverse
**Cloning insert PCR sequences**	**Gene name**	**forward/reverse**
GTAGAATTCGGCCACCATGTGCAATACCAACA	EcoR1-MDM2	forward
GTAGGATCCCCGGGGAAATAAGTTAGCACAATCA	BamH1-MDM2	reverse

Table S3. The list of MDM2 inhibitors used in this study and binding affinity

No.	Molecule name	Dissociation constant (K_d) value (nM)	Reference (K_d)	Reported drugs Conc. (μM)	Reference (drug conc.)
1	Nutlin-3	240	BindingDB (BDBM31197)	1-10	[35]
2	Nutlin-3a	-		10	[36]
3	RG7112 (RO5045337)	11	[37]	5	[35]
4	Idasanutlin (RG7388)	9.8	[38]	~1.8	[39]
5	Navtemadlin (AMG-232)	0.045	[40]	10	[41]
6	Alrizomadlin (APG-115)	-		~1	[42]
7	NVP-CGM097 (CGM097)	2.3	[43]	~1	[43]
8	Siremadlin (NVP-HDM201)	-		10	[44]
9	Milademetan (DS-3032)	-		~2	[45]
10	MI-773	8.2	[46]	~20	[47]
11	YH239-EE	300	[48]	20	[48]
12	NSC 66811	-		20	[49]
13	Lithocholic acid	660	[50]	400	[50]
14	α-Mangostin	-		10	[51]
15	*R*,*S*-Gambogic acid	-		10	[51]
16	SJ-172550	-		~15	[52]
17	Serdemetan (JNJ-26854165)	-		~10	[53]
18	RITA	1.5	[54]	10	[54]
19	SP-141	43	[55]	~1	[55]
20	Cytarabine hydrochloride (MK-8242)	-		~10	[56]
21	RO-5963	-		~20	[57]
22	MI-1061	1.4	[58]	~0.3	[18]
